# Genetic variation in recalcitrant repetitive regions of the *Drosophila melanogaster* genome

**DOI:** 10.1101/gr.280728.125

**Published:** 2025-09

**Authors:** Harsh G. Shukla, Mahul Chakraborty, J.J. Emerson

**Affiliations:** 1 Department of Ecology and Evolutionary Biology, University of California Irvine, Irvine, California 92697, USA;; 2 Graduate Program in Mathematical, Computational and Systems Biology, University of California Irvine, Irvine, California 92697, USA;; 3 Department of Biology, Texas A&M University, College Station, Texas 77843, USA;; 4 Center for Complex Biological Systems, University of California Irvine, Irvine, California 92697, USA

## Abstract

Many essential functions of organisms are encoded in highly repetitive genomic regions, including histones involved in DNA packaging, centromeres that are core components of chromosome segregation, ribosomal RNA comprising the protein translation machinery, telomeres that ensure chromosome integrity, piRNA clusters encoding host defenses against selfish elements, and virtually the entire Y Chromosome. These regions, formed by highly similar tandem arrays, pose significant challenges for experimental and computational studies, impeding sequence-level descriptions essential for understanding genetic variation. Here, we report the assembly and variation analysis of such repetitive regions in *Drosophila melanogaster*, offering significant improvements to the existing community reference assembly. Our work successfully recovers previously elusive segments, including complete reconstructions of the histone locus and the pericentric heterochromatin of the X Chromosome, spanning the *Stellate* locus to the distal flank of the rDNA cluster. To infer structural changes in these regions where alignments are often not practicable, we introduce landmark anchors based on unique variants that are putatively orthologous. These regions display considerable structural variation between different *D. melanogaster* strains, exhibiting differences in copy number and organization of homologous repeat units between haplotypes. In the histone cluster, although we observe minimal genetic exchange indicative of meiotic crossing over, the variation patterns suggest mechanisms such as unequal sister chromatid exchange. We also examine the prevalence and scale of concerted evolution in the histone and *Stellate* clusters and discuss the mechanisms underlying these observed patterns.

Tandem repetitive sequences are major components of many eukaryotic genomes (Richard et al. 2008) and play vital roles in both biology (Gemayel et al. 2010; Hannan 2012) and disease (Hannan 2018). Loci encoding components of important cellular processes are sometimes present in multiple copies and are often clustered in tandem, including examples like histone genes (Maxson et al. 1983), rDNA arrays (Hall et al. 2022), and immunoglobulin genes (Watson and Breden 2012). Tandem repeats are prone to rapid copy number changes mediated through mechanisms such as unequal crossing over, replication slippage, gene conversion, and intrachromatid homologous recombination (Smith 1976; Charlesworth et al. 1994; Johnson and Jasin 2000; Cohen et al. 2003). They also evolve rapidly, as evidenced by the rapid turnover of satellites both in abundance (copy number) as well as specific repeat types between species (Wei et al. 2018; Cechova et al. 2019). Elucidating the mutational mechanisms and evolutionary forces acting on the tandem arrays requires reliably discovering and distinguishing alleles within and between individuals (the “fundamental datum” in Hubby and Lewontin 1966; Lewontin and Hubby 1966; Charlesworth et al. 2016). However, most contemporary reference genome assemblies, including those resulting from consortium-led efforts, contain gaps in such repetitive regions (Altemose et al. 2014; Hoskins et al. 2015; Nurk et al. 2022). This limitation constrains routine comparisons between tandem repeat arrays necessary to elucidate the structure, function, and evolution of such arrays.

The rapid development of long-read sequencing technologies (Marx 2023) and informatics approaches (Chin et al. 2013, 2016; Berlin et al. 2015; Koren et al. 2017) led to a proliferation of genome assemblies that approached the quality of consortium-led reference sequencing projects. In *Drosophila*, these advances led to enormous improvements in the ability of single research groups to rapidly and inexpensively construct high-quality genome assemblies (Chakraborty et al. 2016, 2018, 2019, 2021; Mahajan et al. 2018; Miller et al. 2018; Solares et al. 2018; Hill et al. 2019; Kim et al. 2021; Liao et al. 2021).However, owing to its inherently noisy nature, some repetitive parts of the genome were still recalcitrant to assembly approaches (Nurk et al. 2020; Rhie et al. 2021). Whereas sophisticated application of long-read data to assembly problems has unarguably led to several examples of advancing the state of the art in previously recalcitrant regions of the genomes (Khost et al. 2017; Bongartz and Schloissnig 2019; Chang and Larracuente 2019), these approaches were not scalable to routine application by typical research groups, requiring extensive novel work on assembly informatics before downstream analysis was possible. Indeed, despite the promise of long-read approaches, cytogenetic approaches remain indispensable to studying genetic variation in such regions (Courret and Larracuente 2023).

Consequently, studies of variation in many parts of the genome harboring tandem repeat arrays remain challenging. These challenges become especially pronounced when appraising the relationships between genes copies within and between species. When a gene family originates before two species diverge, orthologs are expected to be more similar than paralogs. However, paralogs are often found to be more similar than orthologs, suggesting a homogenizing force within species acts after speciation (Hood et al. 1975; Dover 1982; Liao 2008). This homogenization of repeat units, termed concerted evolution (Elder and Turner 1995), is mediated through DNA recombination, repair, and replication mechanisms such as unequal crossing over and gene conversion (Liao 1999). However, the extent of homogenization and variation in observed copy numbers may vary across tandem arrays and may be shaped by various factors (such as natural selection, drift, mutation, drive, age, size, and relative rates of different kinds of recombination) acting on the array (Dover et al. 1993; Elder and Turner 1995; Lower et al. 2018). Untangling patterns of concerted evolution on tandem arrays experiencing different forces requires a comprehensive map of genetic variation spanning such clusters.

The cluster of highly conserved histone genes on Chromosome arm 2L and the species-specific X-linked cluster of *Stellate* (*Ste*) genes are examples of two recalcitrant tandem arrays in *D. melanogaster* shaped by different evolutionary forces. The histone locus has a tandem organization with the quintet of five histone genes (*H1, H2B, H2A, H4, H3*, from distal to proximal) arrayed in tandem for ∼100–110 times (Lifton et al. 1978; Strausbaugh and Weinberg 1982). Each unit is ∼5 kb in length, and this tandem organization is present in distantly related *Drosophila* species, indicating old evolutionary origins (Kakita et al. 2003). In contrast, *Stellate* is a *D. melanogaster*-specific, evolutionary young tandem cluster thought to have arisen through the intragenomic conflict between X and Y Chromosomes (Hurst 1992; Malone et al. 2015; Martí and Larracuente 2023; but also see Belloni et al. 2002). It is present at two distinct locations on the X Chromosome: one in euchromatin and the other in heterochromatin, and each unit is 1.26 kb and 1.15 kb in size, respectively (Palumbo et al. 1994; Adashev et al. 2021). In wild-type male flies, the *Stellate* locus is normally suppressed by piRNAs originating from the complementary *Su(Ste)* loci on the Y Chromosome. If left unsuppressed, *Stellate* RNA produces star/needle-like crystal protein aggregates in primary spermatocytes, leading to meiotic abnormalities and fertility defects (Adashev et al. 2021). Studying genetic variation within these two tandem arrays can help us understand the general and unique properties of the mutational mechanism and concerted evolution driving the evolution of these complex regions of the genome. However, we lack a detailed map of genetic variation within tandem arrays like histone or *Stellate* clusters, limiting inferences of molecular mechanisms or patterns of evolution in such clusters.

Here, we present analyses of genome structure for three strains of *D. melanogaster* that address such challenging regions of the *D. melanogaster* genome, including the histone and *Stellate* clusters. To develop a scalable approach for studying genetic variation within tandem arrays, we applied widely used software to easily collectable highly accurate long-read data (Wenger et al. 2019) to assemble the recalcitrant repetitive regions of the genome and developed a new framework to reveal the detailed pattern of structural variation in repeat clusters. Using a comprehensive map of variation within the histone and *Stellate* clusters, we explore patterns of variation that empower us to comment on mutational processes shaping two functionally important tandem arrays in the *Drosophila* genome.

## Results

### Genome assemblies

**Figure 1.**
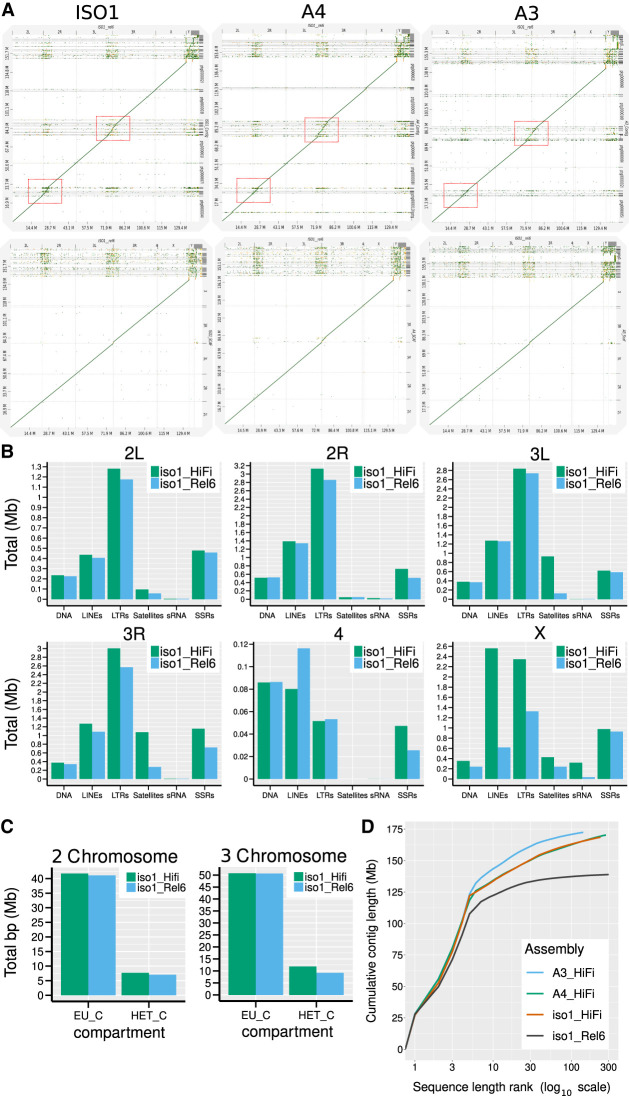
Comparison of assemblies here to that of Release 6. (*A*) D-Genies dot plots of the Release 6 reference genome assembly scaffolds for *Drosophila melanogaster* (*x*-axis) versus our contig- (*top*) and scaffold-level (*bottom*) assemblies of iso-1, A4, and A3. (*B*) Repeat content comparison of iso-1 Release 6 assembly versus our iso-1 HiFi assembly for each Muller element (i.e., autosomal chromosome arms and the X Chromosome). (*C*) Comparison of total sequence assigned to euchromatic and heterochromatic compartments in iso-1 Rel6 versus iso-1 HiFi for 2 and 3 Chromosomes. (*D*) Cumulative length plot for assembly contigs of strains used in this study and iso-1 Release 6. The *x*-axis is on a log_10_ scale.

The strains selected include the *Drosophila* community's genome reference strain (iso-1) (Adams et al. 2000) and two strains from the *Drosophila* Synthetic Population Resource (A3 and A4) (King et al. 2012), all of which are near isogenic. We performed de novo assembly with deep coverage (iso-1: 97×, A4: 163×, A3: 156×) Pacific Biosciences (PacBio) HiFi reads (Wenger et al. 2019) for each strain. The assemblies are very contiguous and complete, exhibiting contig N50s (L50s) of 22.96 Mb (4), 21.53 Mb (4), and 21.50 Mb (4) for the iso-1, A4, and A3 assemblies, respectively, as well as recovering universal single-copy orthologs (i.e., BUSCOs [Manni et al. 2021]) at very high rates of 99.42%, 99.27%, and 99.42%. A subsampling analysis of the iso-1 data indicates that greater depth would not have significantly improved our assemblies, and, whereas lower read depth could have been sufficient for euchromatin, it may have compromised heterochromatin assembly (Supplemental Fig. 1). The assemblies exhibit very few base-level errors, exhibiting Phred (QV) scores of 49.9, 56.1, and 55.3 with estimates of heterozygosity for these isogenic strains of 0.076%, 0.071%, and 0.081% (Supplemental Figs. 2–4) for iso-1, A4, and A3, respectively. The chromosome arms are mostly represented by single contigs spanning the entire euchromatin (Fig. 1A). Gaps appear in highly repetitive heterochromatic regions of the genome (Fig. 1A; red boxes occurring in pericentromeric regions of Chromosomes 2 and 3). The median read length (15–20 kb) of HiFi sequences is not sufficient to span many satellites and other repetitive sequences (Porubsky et al. 2023) enriched in the pericentromeric and centromeric heterochromatin (Jagannathan et al. 2017; Shatskikh et al. 2020). The iso-1 HiFi assembly was scaffolded using both Hi-C data and reference-assisted scaffolding (Supplemental Fig. 5). In autosomes (Muller elements B-F), ∼4.17 Mb of new sequence was added relative to Release 6, most of it (∼3.29 Mb) in heterochromatic regions (Fig. 1C). For the X Chromosome (Muller element A), our assembly also introduced ∼3.9 Mb of new sequence, including ∼2.76 Mb of heterochromatin sequence adjacent to the rDNA array. For every major repeat category across all chromosomes except the dot, the HiFi assembly of iso-1 recovered more than previous assemblies (Fig. 1B). We also identified a total of 15.7, 16.48, and 17.03 Mb of contigs putatively belonging to the Y Chromosome in iso-1, A4, and A3, respectively. The iso-1 Y assembly corresponds well in structure and repeat content to a recently reported assembly (Supplemental Fig. 6; Chang and Larracuente 2019). The assemblies of A3 and A4 were scaffolded using comparative scaffolding with our iso-1 HiFi scaffolded assembly as the reference. The assemblies exhibited scaffold N50s (L50s) of 27.44 Mb (3), 27.72 Mb (3), and 28.05 (3) for iso-1, A4, and A3, respectively. In total, the chromosome scaffold for all three strains spans ∼142 Mb. These assemblies are superior to existing high-quality reference-grade assemblies like iso-1 Release 6 (Fig. 1D). The statistics of the data and assembly generated can be found in Supplemental Tables 1–5.

### Newly assembled X-linked heterochromatic sequence

**Figure 2.**
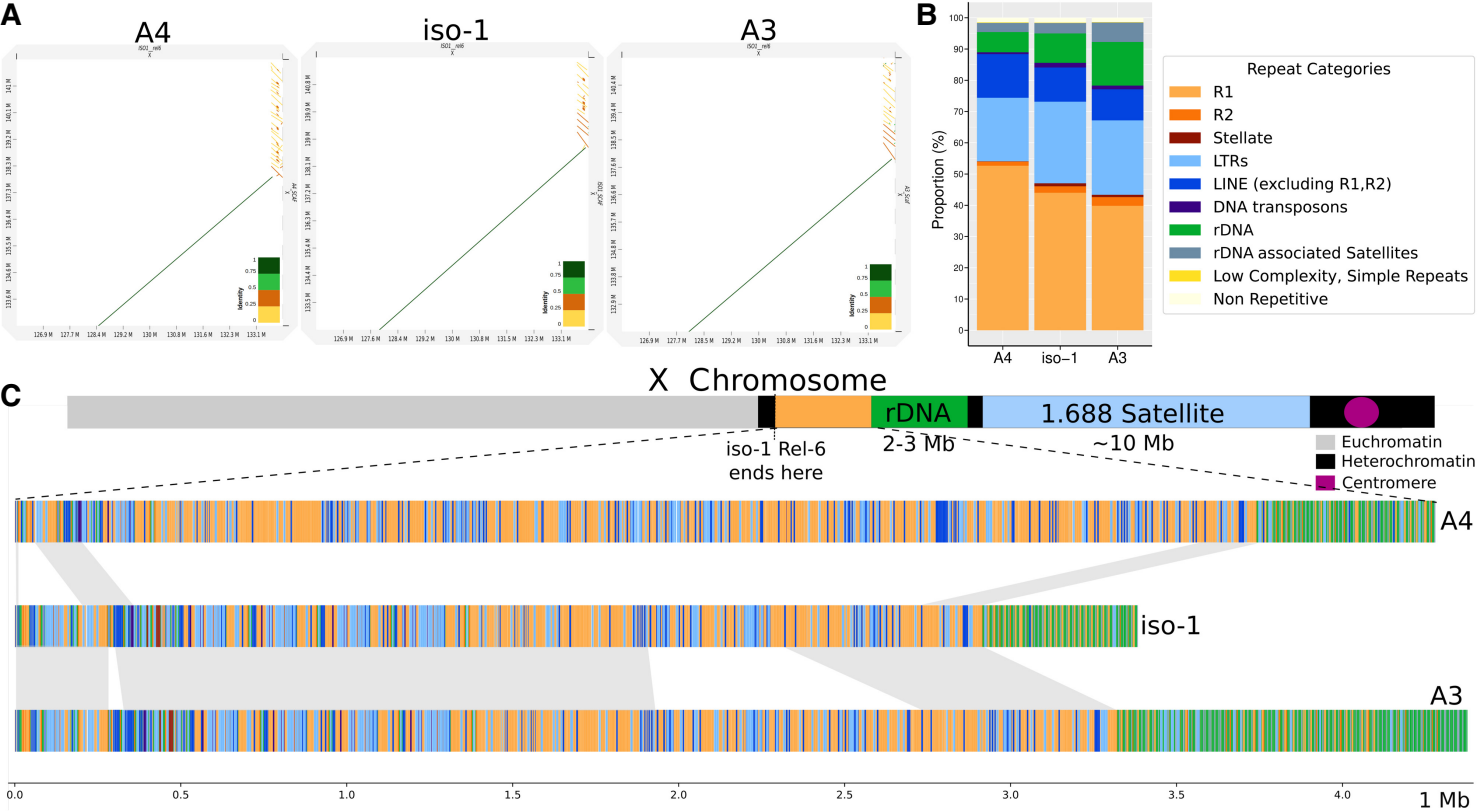
Characterization of newly assembled X-linked heterochromatic sequence. (*A*) D-Genies dot plots of our HiFi assemblies (*y*-axis) versus the Release 6 assembly of iso-1 (*x*-axis) covering the proximal end of X Chromosome scaffold for both assemblies. (*B*) Repeat catalog of the newly assembled proximal regions on the X Chromosome for different strains. (*C*) Map of sequence from the newly assembled proximal end of the contigs assembled here. *Top*: Schematic map of X Chromosome structure. *Bottom*: Structure of the region in the HiFi assemblies of the X Chromosome for the haplotypes in A4, iso-1, and A3. The haplotypes are painted with colors representing different repeat categories (following panel *B*).

The heterochromatic portions of the genome have been subdivided into their own bands based on cytological features (Gatti and Pimpinelli 1983, 1992; Kaufman 2017). The heterochromatin of the X Chromosome spans bands h26-h34, out of which only h26 and a small part of h27 are represented in the chromosome-scale scaffolds belonging to iso-1 assembly on FlyBase. Our iso-1 assembly recovers a single contig spanning the entire X euchromatin and heterochromatic bands h26-h28 and 454 kb of h29, the band containing the rDNA gene cluster. Alignments of the X Chromosome between HiFi assemblies from iso-1, A4, and A3 to Release 6 (Fig. 2A) show that the newly assembled segments h27-h28 comprise arrays of segmental duplications with varying copy numbers in the three strains. The entire region (∼98.5%) is repetitive, with two repeat elements—LINE *R1/R2*—dominating the landscape, although *R1* is by far the more abundant of the two (Fig. 2B). Further analysis revealed these flanking *R1* elements are predominantly fragmented, differing from the intact, full-length *R1* sequences observed within the main rDNA array (see Supplemental Note 1, Section 1). Figure 2C compares the whole region across the three assemblies. The iso-1 and A3 strains exhibit similar haplotype structures, except A3 harbors one additional large multi-kb segment. The A4 haplotype varies considerably from the other two, with only part of the proximal and distal ends of the array aligning. The intrastrain self-dot plots illustrate this, with iso-1 and A3 (Supplemental Fig. 7) exhibiting similar patterns, whereas A4 is quite different. To verify the integrity of the newly assembled region, we mapped HiFi data back to the assembly and inspected the depth in this region. Dramatic variation in depth plots may indicate errors in the assemblies, such as putative misassemblies, collapsed or expanded duplications, etc. The depth plot (Supplemental Fig. 8) for iso-1 indicates that a region of ∼250 kb immediately distal to the rDNA exhibits twice the coverage, indicating possible collapse in our assembly. The plots for A4 and A3 (Supplemental Fig. 8) show relatively uniform coverage, indicating no analogous large-scale misassemblies. A schematic figure comparing the end of X heterochromatin assembly in Release 6 with our iso-1 assembly is presented in Supplemental Figure 9 to facilitate a more detailed comparison.

### Structural variation in the histone cluster

#### Assembly of D. melanogaster histone cluster for iso-1, A4, and A3

The histone locus in *Drosophila melanogaster* is present at the 39DE region of Chromosome 2L, bordering the pericentromeric heterochromatin. The highly homogeneous nature of this locus has impeded its resolution to the base pair level. The current reference iso-1 Rel6 has only ∼69 kb (12 units) assembled on the distal flank and ∼56 kb (11 units) assembled on the proximal flank. Our default assembly model of the histone locus in iso-1 spans the entire locus without gaps. The array is ∼577 kb, comprising a total of 111 units. This is consistent with expectations from the literature. We mapped the raw HiFi read data back to the assembly to quantify the read coverage across the locus and validate this result. In a well-assembled region, the read coverage should be uniform, lacking sudden and dramatic changes in coverage in a short span. The sudden drop and rise in coverage and doubled coverage (at ∼200 kb and ∼50 kb) suggest the presence of a misassembly and a small collapse at the beginning of the cluster, respectively (Supplemental Fig. 10). To address these features, we employed a targeted assembly approach where we identified reads mapping to the histone locus. We then assembled them with parameters more appropriate for repeats (see Methods). The targeted assembly exhibited a locus size of ∼587 kb and 113 copies. The coverage plot of the targeted assembly is comparatively uniform, with no sudden rise and drop in coverage, suggesting that the misassembly is no longer present (Supplemental Fig. 10). It also no longer possesses the doubled coverage indicative of a collapsed duplication. The putative collapsed tandem duplication spanned two units (units 7–8 and 9–10 in the targeted assembly), increasing the total unit copy number by two in the targeted assembly. The putative misassembly affected unit 39 (unit 37 in the original assembly). Other than these differences, the structure of both assemblies remains the same between the default assembly and the targeted assembly. The assembly graph around the histone locus in the original assembly had only three unitigs (Supplemental Fig. 11A). They overlap with each other at the same junction, with smaller unit 842l having an overhang. Thus, there was only one possible path through it. This junction is near the collapsed duplication, suggesting a cause for the collapse. Although the unitig graph of the targeted assembly had more unitigs, the path through it was relatively clear (Supplemental Fig. 11B).

We compared our assembly of iso-1 to that of Bongartz and Schloissnig (2019), who employed machine learning to assemble the histone cluster using published long-read data (Kim et al. 2014). The structure of this assembly (henceforth referred to as iso-1-BS19) was mostly consistent with ours, with units 2–107 of iso-1-BS19 corresponding to units 6–111 of ours. All discrepancies between the assemblies were located at the edges of the cluster (Supplemental Fig. 12). Segregating variation and/or minor misassemblies may explain these observations (see Discussion). Units 1–4 of our assembly were missing in iso-1-BS19. The iso-1-BS19 starts with a partially assembled unit containing *LTR/roo* corresponding to the fifth copy in our assembly. On the other side of the cluster, the DNA/*Pogo*-containing unit is the final unit (113) in our HiFi assembly, whereas it is the penultimate copy (unit 108) in the iso-1-BS19 version. Instead of the final copy (corresponding to unit 113 in our assembly), iso-1-BS19 has a partially complete unit (∼3.3 kb). By aligning the two assemblies across ∼105 units (corresponding to units 6–111 in our assembly and alignment block length of ∼540 kbp), we discovered only two SNPs and two 1-bp indels associated with homopolymer tracts of length 8 and 13.

The default assembly for A4 constructed the histone cluster into 119 copies. The read depth plot shows a drop in coverage (about half) for around ∼40–50 kb towards the end of the cluster (Supplemental Fig. 13). This indicates that a region present once in the genome is represented twice in the assembly. The unitig graph (Supplemental Fig. 14A) in that region shows two unitigs (963l and 738l) that overlap on both ends. It appears that hifiasm visited the 963l node twice, leading to the duplication. The targeted assembly of the histone locus has 111 copies, totaling ∼571 kb. The read depth plot shows relatively uniform coverage and no apparent misassembly or collapse/expansion (Supplemental Fig. 13). The previous drop in coverage also disappears. The targeted unitig graph has a slightly complex region in the beginning, but hifiasm resolves it (Supplemental Fig. 14B).

The default assembly for A3 constructed the histone cluster into 114 copies. Depth plots identified a putative misassembly at ∼200 kb immediately followed by several large collapsed regions (Supplemental Fig. 15). The default unitig graph around the histone locus is more complex than either iso-1 or A4 (Supplemental Fig. 16A). Moreover, a targeted assembly with modified parameters failed to assemble the cluster, exhibiting breaks, suggesting that the structure of A3's histone cluster possessed features more recalcitrant to assembly than either iso-1 or A4, like large and homogeneous duplications. This also hinted that the histone unit copy number was significantly higher than the typical expectation of ∼110. To validate our findings, we sequenced PCR-free Illumina libraries derived from the three strains (the same ones in our lab that were used to generate HiFi data). We adapted the idea developed by McKay et al. (2015) for estimating copy numbers. Briefly, we masked the entire histone locus in the iso-1 HiFi assembly, introduced only a single copy of the histone unit as a separate contig, and mapped Illumina data to it. The relative ratio of the depth of the histone contig (one unit) versus the depth of any single copy region should approximate the total number of histone units present in the genome. Such an estimate would be a lower bound because only one unit is present, and any variation between units could lead to reduced mapping. We measured the ratio of the median depth of the histone contig to the median depth of the 2L Chromosome. Both iso-1 and A4 showed results consistent with, but slightly lower than, observed in the assemblies (102 for iso-1 and 102 for A4). However, the A3 coverage showed an elevated copy number of 197, almost double the typical copy number (Supplemental File 1). Because the standard targeted assembly approach for A3 did not yield a contiguous assembly, we subsampled the longest 38 Mb of reads, which would be 40× if the locus were 950 kb (∼190 units) in length. These reads were then used to construct a final targeted assembly based on longer reads, which led to three contigs spanning ∼134 units, which is still shy of our conservative prediction of 197. As a result of this inability to reconstruct the entire histone cluster in A3 without gaps, downstream analyses focus on only the well-resolved parts of the locus (Supplemental Fig. 16B). The unitig graph from the longest 40× data shows a very complex region in the middle exhibiting possibly circular paths (Supplemental Fig. 16B). This likely corresponds to large duplications indicated by the read depth plots. The correct path through it cannot be unambiguously determined. For our final assembly, we considered the well-resolved parts on the left (∼193 kb) and right (∼307 kb) flanks and joined them with a gap containing Ns. The total size of our gap-containing model of the locus was ∼500 kb and had 97 units (36 on the left flank and 61 on the right).

Whereas the results presented are from our targeted hifiasm assemblies, we also evaluated other HiFi assemblers (Bankevich et al. 2022; Rautiainen et al. 2023). The hifiasm (Cheng et al. 2021) approach produced the most contiguous models of the histone locus. A detailed comparison shows that our conclusions are not sensitive to the assembler used (Supplemental Note 1, Section 2).

#### Inferring histone cluster copy numbers in the global diversity lines

To estimate the copy number in the histone cluster in stocks when no high-quality assembly exists, we used the depth of Illumina data. We focused on a reference collection of 85 *Drosophila* stocks representing genetic diversity across the globe (i.e., the Global Diversity Lines or GDL) (Grenier et al. 2015). The mean and median histone copy numbers in GDL were 101.27 and 104, respectively. The lowest copy number we observed was 29, and the highest was 146 (Supplemental File 1). The standard deviation was 20.56. Supplemental Figure 17 shows the distribution of inferred copy numbers in GDL.

We took advantage of replication and deep sequencing in the reference collection to estimate the error in copy number estimated from our approach. One stock (ZW155-ST/ST) was sequenced twice, once to 12.5× (the average depth for all samples) and once to a depth of ∼100×. Our pipeline estimated 81 copies for the low-depth sample and 75 copies for the high-depth sample. To evaluate whether depth was responsible for this discrepancy, we randomly subsampled the high-depth sample to ∼12.5× and estimated the copy number 100 times. The results ranged from 74 to 77 copies, indicating that the variation between the two replicates was likely not due to the sequencing depth, suggesting that experimental steps preceding the informatics are likely the source of the modest discrepancy exhibited by our estimates.

In the GDL, half of the stocks showed copy numbers between 90 (Q1) and 112 (Q3), roughly consistent with reports from the literature (100–111) (Lifton et al. 1978; Strausbaugh and Weinberg 1982). Notably, half of the estimates were outside those boundaries, suggesting substantial natural variation in histone copy number. The lowest copy number estimate we observed (29) exceeds the experimentally derived floor for copy number (20) required for normal development (Zhang et al. 2019), and the highest (146) exceeds the typical copy numbers of 100–110 by more than 40%. Although the numbers we report are only estimates of the true number, the error is unlikely to explain most of the variation in our observations. The discrepancy between estimates based on assembly and Illumina read depth for iso-1 and A4 was about 10%, whereas the range of the estimates of the two replicates for ZW155-ST/ST was also about 10% (74–81). To validate the long read assembly and Illumina read mapping approaches above, we employed two additional orthogonal approaches. First, a dPCR assay performed on our strains and several GDL outliers confirmed the large (three- to fourfold) magnitude of copy number variation (see Supplemental File 6). Additionally, a mapping-free *k*-mer analysis of our HiFi data also corroborated our findings. Together, these results bolster our conclusion of extensive copy number heterogeneity, and a detailed comparison of all four approaches is presented in Supplemental Note 1, Section 3.

#### Comparative genomics of the histone cluster

**Figure 3.**
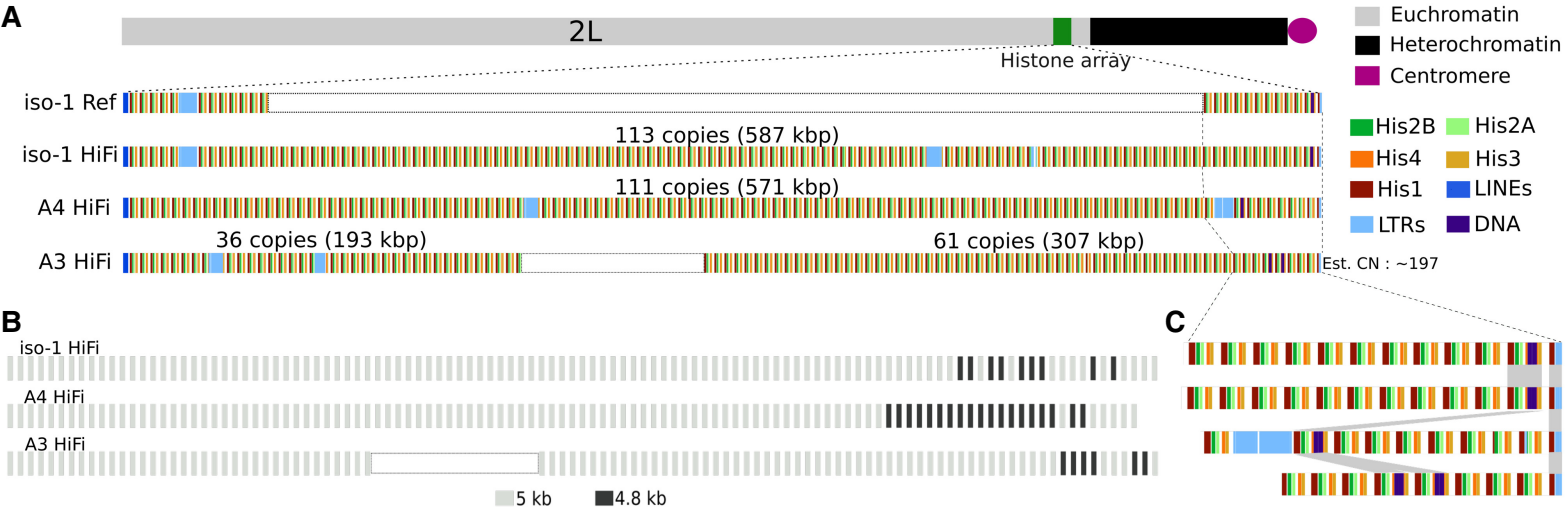
Maps of the newly assembled histone locus in *Drosophila melanogaster*. (*A*) Overview of the structure of the histone locus. *Top*: Schematic illustration of the location of the histone locus relative to the rest of Chromosome 2L. *Bottom*: Map of location of elements in the histone cluster, including the five individual histone genes and various transposable elements. “iso-1 Ref” refers to the Release 6 community assembly and “iso-1 HiFi” indicates the assembly presented here. White rectangles for iso-1 Ref and A3 HiFi indicate gaps resized to facilitate comparison with the other two assemblies. (*B*) Location distribution of the major histone unit types (i.e., 5-kb and 4.8-kb). (*C*) Expanded map of the proximal end of the array. Order of strains follows panel *A*.

Figure 3A shows a schematic view of the histone locus across four different assemblies: iso-1 (FlyBase), iso-1 (HiFi), A4, and A3. The iso-1 FlyBase assembly contains a gap represented by empty space and is scaled to match our assembly's 113 copies. The black box in the A3 assembly separates the left part (∼36 units) and the right part (∼61 units) of the assembled flanks of the cluster. The light blue boxes represent different LTR TE insertions in the locus. Two main length variants of histone cluster units in the *Drosophila melanogaster* are 4.8 kb and 5 kb (Colby and Williams 1993). The 5-kb repeat unit has a 242-bp insert between *H1* and *H3* genes. These variants are present in a number of *Drosophila* species, indicating that their origin predates the speciation event (Strausbaugh and Weinberg 1982). Figure 3B shows the distribution of 5- and 4.8-kb types in the assemblies. In the three strains, the 5-kb repeat significantly outnumbers the 4.8-kb one, and they are generally clustered separately, with 4.8-kb found exclusively towards the proximal flank of the array.

Figure 3C shows an expanded version of the proximal end of the array. Given how the relative location of the *DNA/POGO*-containing unit varies within the array, the array has clearly expanded and/or contracted since the haplotypes in our sample last shared a common ancestor. To better understand the array dynamics, we sought to align the arrays and possibly comment on the mutational mechanism active in the array.

#### Anchored comparisons between highly repetitive arrays

**Figure 4.**
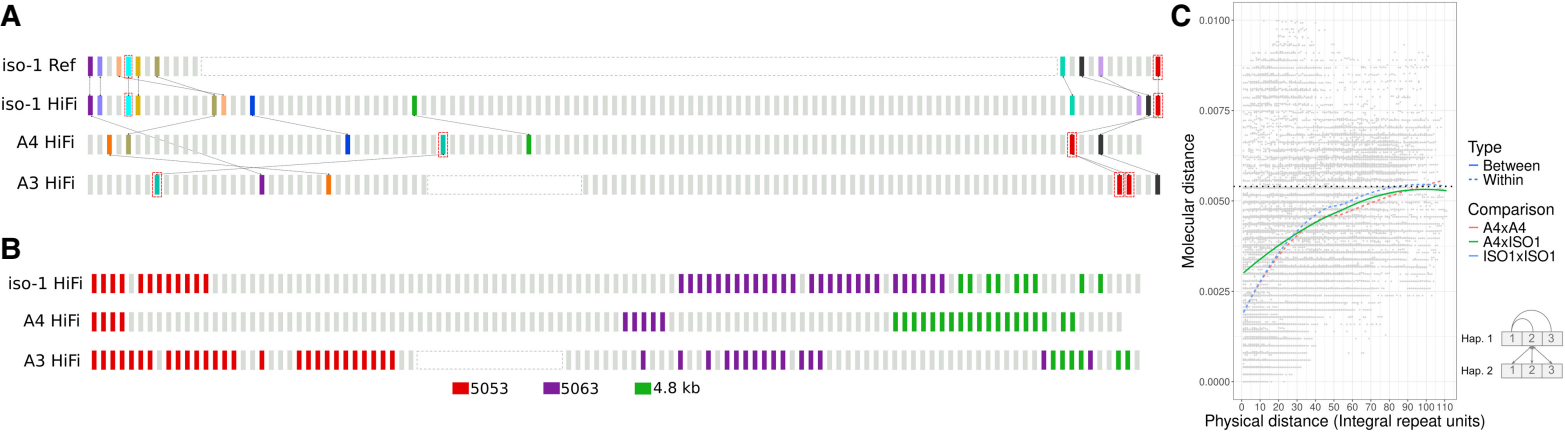
The histone locus is a dynamic array characterized by extensive rearrangements across different *D. melanogaster* haplotypes. (*A*) Distribution of landmark anchors used in this study. Anchors enclosed in red boxes indicate units harboring TE insertions. Other anchors are identified by phylogenetic grouping (see Methods). Anchors sharing the same color are putatively homologous. Lines between strains highlight the positions of these putative homologs in their respective haplotypes. (*B*) Distribution of unit types along the histone cluster in three haplotypes, including two additional subtypes of 5-kb: 5053 and 5063. (*C*) Graphical representation of physical distance between histone units on the chromosome (*x*-axis) and molecular distance (*y*-axis). Molecular distances represent all unique pairwise comparisons either between or within strains. *Bottom right*: Schematic diagram how molecular distance comparisons between and within strain comparisons were conducted.

To assess variation in the structure of arrays between different strains, we compared segments flanked by putatively unique anchor sequences. Even though the repeat units are too similar to each other to construct reliable alignments that are biologically meaningful, comparing haplotype segments flanked by the same anchors in different strains permits us to establish a conservative floor for estimates of structural variation. By comparing various metrics (e.g., the number, types, and arrangements of units) in these segments, we can determine whether single duplication or deletion events explain these changes or if the changes are more complex. Units with unique features that occur only once in each haplotype in a given comparison can serve as unique landmarks we call anchors. This is the same approach we used above for *DNA/POGO* TE-containing units. For example, when a TE is inserted into the array, the combination of the specific insertion location and TE type establishes a landmark that can be used as an anchor, provided it has not spread widely throughout the array through duplication and/or gene conversion. From this type of anchor landmark alone, we can already determine that the histone cluster experiences rapid expansions and/or contractions. The red boxes in Figure 4A indicate such landmarks and occupy substantially different positions in the cluster between strains. Here, the TE insertion serves to mark the unit. However, any other reliably identifiable mutation unique in the array (e.g., deletions, insertions, or even unique SNPs) can serve as candidates for landmark anchors.

To systematically identify more putative anchors, we extracted individual units in the arrays for iso-1-Rel6, iso-1, A4, and A3. For every pairwise comparison, we drew a neighbor-joining tree for all units in both strains (500 bootstrap replicates, 50% cutoff for consensus tree). When two units from two different strains are clustered together, we mark them as unique anchor points. TE-containing units were analyzed separately, as described above. In addition to the unit containing *DNA/POGO*, one unit harboring a *HMSBEAGLE_I* insertion was present in A4 and A3 but not iso-1. Figure 4A summarizes the structure of the histone units in relation to the anchors. Such putatively orthologous copies are marked using the same color. The TE-containing anchors are marked by red boxes. The sequence flanked by the same adjacent anchors in different strains is often dramatically different in size and/or composition, indicating a rapid turnover of repeat units in the cluster with units being added and deleted. This is true even for alternate assemblies of the same strain. (N.B.: These alternate assemblies are based on data collected in different years in different laboratories.) In the relatively small fraction of the histone cluster present in the iso-1 Rel6 reference, the structure is not identical to the corresponding segments in our iso-1 HiFi assembly. The periphery of the histone locus was first added to the assembly in Release 4 (https://www.fruitfly.org/annot/release4.html). If the assembly of these regions in FlyBase is correct, it suggests that rapid evolution has occurred since iso-1 DNA was collected for the FlyBase reference and when it was collected for this study. This observation mirrors similar observations regarding structural variation in a previous study (Solares et al. 2018). In that study, several TE insertions and copy number changes affecting a 207-bp tandem array in *Muc26B* distinguished the two assemblies, indicating fast evolution.

#### Variation between histone units within and between strains

Histone repeat units are very similar to one another. To quantify the structure of this homogeneity, we annotated the distribution of various unit types across the cluster. To identify closely related repeat units, we focused on clades in pairwise phylogenetic trees composed of units from both strains. As expected, the 4.8-kb units are distinct from the other units, clustering together. We also identified two more unit types, 5053 and 5063, based on the median length of the identified units in the clades. Figure 4B shows the spatial distribution of these three broad unit types in the three strains. Two observations stand out: (1) the different unit types are present in different copy numbers in different strains; (2) the unit types tend to cluster together.

Next, we compared the similarity between histone units and their positions along the array, both within and between strains. Figure 4C shows the relationship between physical distance (*x*-axis) and molecular (i.e., evolutionary) distance (*y*-axis) between units in the array. For adjacent units in the same strain, the average molecular distance averaged within both strains is 0.12% (range: [0.112, 0.131]), more than tripling to 0.43% (range: [0.41, 0.46]) after about 30 units. However, when two units occupy adjacent positions in different strains, their average divergence is about 2.5-fold higher, or ∼0.31%, increasing to 0.41% after about 30 units. For longer separations, comparisons both within and between strains slowly asymptote from ∼0.41% when 30 units apart to ∼0.55% when 90 units apart. We can also examine individual differences between units instead of the average for a given physical distance. In this context, the maximum single difference we observed between any two units within the species is about 1.2%, which is between a 4.8-kb and a 5-kb unit. Within 5-kb units, all comparisons differ by <0.9%. When we compare a randomly chosen unit from iso-1 (Unit 18) in *Drosophila melanogaster* to a 4.6-kb partial histone unit cloned from *D. simulans* (accession AB055959 [Tsunemoto and Matsuo 2001]), the aligned portion of the sequences differ by ∼6.7% without indels or by about 9.2% when indels are included.

#### Linkage disequilibrium (LD) in the histone locus

To measure LD across the histone locus, we used data from the Zambian lines from the *Drosophila* Genome Nexus (DGN) (Lack et al. 2015). We employed this sample population because it is derived from haploid embryos, thereby mitigating issues related to genotyping. Moreover, the Zambian strains show high levels of interstrain variation. Ignoring the stocks with the *In(2L)t* inversion, 147 of 197 haplotypes were analyzed from the Zambian population.

**Figure 5.**
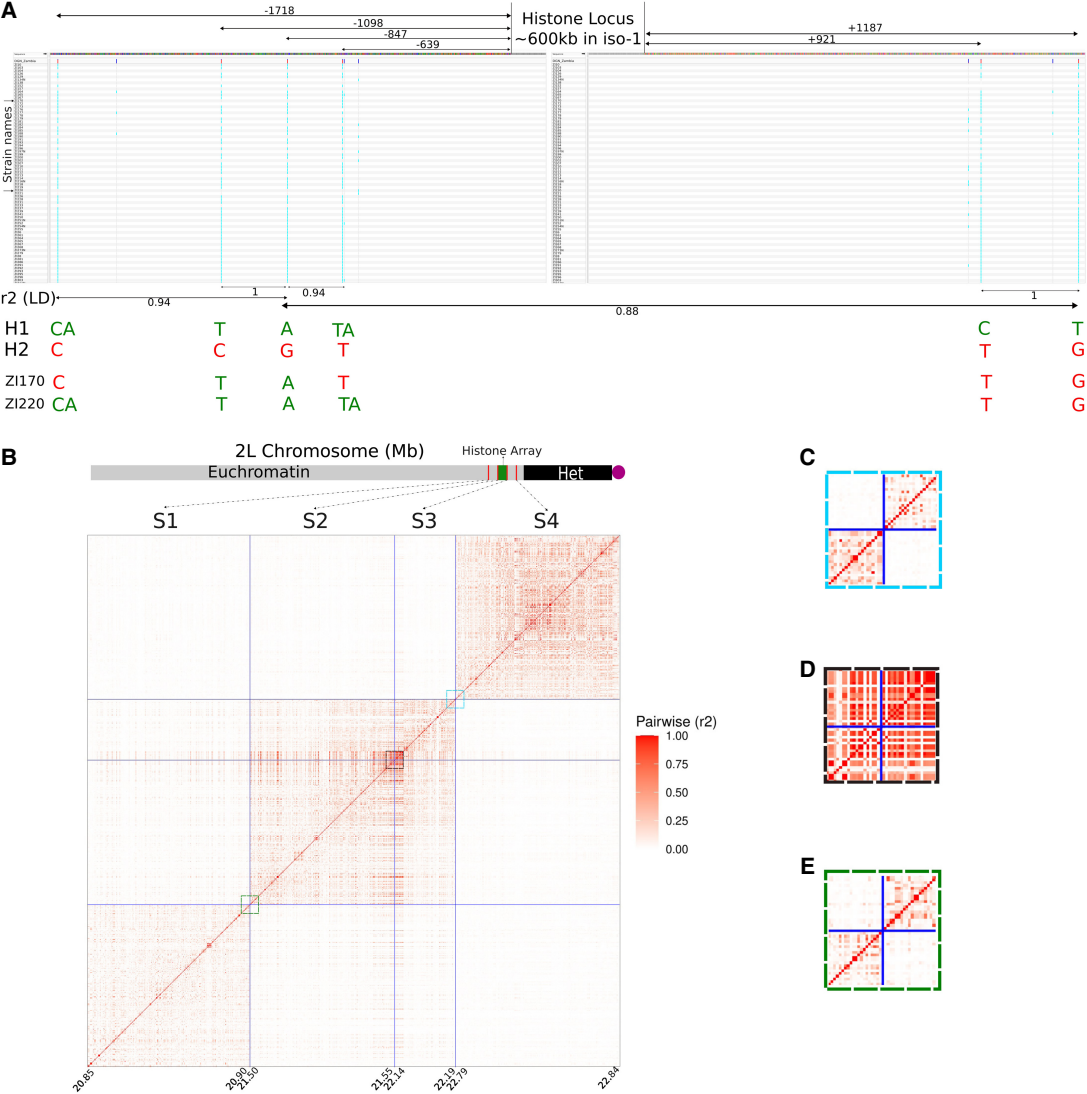
Recombination (crossing-over) in and around the histone locus. (*A*) Integrative Genomics Viewer plot of the regions flanking the histone locus. Values between double-headed arrows under the *x*-axis indicate the *r*^2^ measure of linkage disequilibrium between the variants spanned by the arrows. Coordinates are with respect to the histone locus, with negative values indicating the distal flank and positive values indicating the proximal flank. H1 and H2 each represent haplotypes present in all but two strains. ZI170 and ZI220 possess their own private combinations of variants from H1 and H2 haplotypes and are the only representatives of their respective haplotypes. (*B*) Matrix of *r*^2^ between 4- and 50-kb segments (S1–S4) around the histone locus. Each segment is fixed in physical size. Because only variants are plotted, the plotted size scales with the number of variants in the segment. Each segment is separated by a size determined by the length of the histone cluster in iso-1 (∼600 kb). Colored boxes correspond to expansions in panels *C*–*E*. *Bottom*: Schematic representation of Chromosome 2L and the location of the histone locus with respect to the segments and to the annotated boundaries of the heterochromatin. (*C*) Expanded view of pairwise LD for 50-kb regions flanking a control locus spanning 22.19–22.79 Mb (i.e., S3 and S4). (*D*) Expanded view of pairwise LD for 50-kb regions flanking the histone locus spanning 21.55–22.14 Mb (i.e., S2 and S2). (*E*) Expanded view of pairwise LD for 50-kb regions flanking a control locus spanning 20.9–21.5 Mb (i.e., S1 and S2).

Figure 5A shows the Integrative Genomics Viewer (IGV) plots of two windows flanking the histone locus. We label the flanking SNPs in relation to the position of the histone cluster. Distal positions start with −1 and become more negative the more distal they are. Proximal positions start with 0 and become more positive the more proximal they are. Two major haplotypes span the region, which we call H1 and H2. For the sake of simplicity, we have neglected SNPs and indels with MAF < 5%. However, this does not impact our observations because they are in complete LD (*D*′ = 1) with their respective haplotypes. Out of 147 stocks, 145 are either H1 or H2. Only two samples are exceptions: ZI170 and ZI220.

The first variant on the distal flank is at position −639 bp. The proximal end of the histone cluster terminates in a partial unit of about ∼1.5 kb in all three strains for which we have the assembly. The first ∼1 kb of the proximal flanking region is repetitive and exhibits sparse read coverage. Some SNPs called in this region exhibited elevated rates of missing read data for some strains and had lower-quality mapping scores. For example, SNP +921 had 3.4% missing data and an average MAPQ of 43 (compared to almost 60 elsewhere). The *r*^2^ between the first high-quality SNPs in each flanking region (positions −847 and +1187) is 0.88. In this comparison, although we neglect SNP +921, it is in perfect LD (*r*^2^ = 1) with SNP +1187. Except for only two of the strains mentioned above (i.e., ZI170 and ZI220), the entire collection is in perfect LD (*r*^2^ = 1) between −847 and +1187.

Figure 5B shows pairwise LD on a larger scale. The blue lines demarcate four 50-kb segments. Because the plot depicts pairwise *r*^2^ between sites, it is only defined between two variable sites. As a result, the different sizes of identically sized segments in the plots reflect different SNP densities in those segments. Each segment is separated by ∼600 kb (the size of the histone cluster in iso-1) in positions indicated by the blue lines. The actual histone cluster separates segment pair 2:3, whereas pairs 1:2 and 3:4 are separated by other sequences downstream and upstream of the histone locus, respectively. LD typically decays rapidly in *D. melanogaster*, so two variants separated by ∼600 kb should not be in LD. That holds true for segment 1 versus 2 and segment 3 versus 4 (Fig. 5C,E). However, segments 2 and 3 exhibit extremely high LD even across 600 kb (Fig. 5D). The three HiFi strains are most similar to the H1 haplotype. Although A4 shares this exact haplotype, both iso-1 and A3 possess two private alleles on the H1 background. This is consistent with their origins—A4 was collected from Zimbabwe which is geographically very close to Zambia, whereas A3 originates from Spain, and the origin of iso-1 is uncertain.

### Structural variation in the *Stellate* clusters

#### Euchromatic Stellate cluster

**Figure 6.**
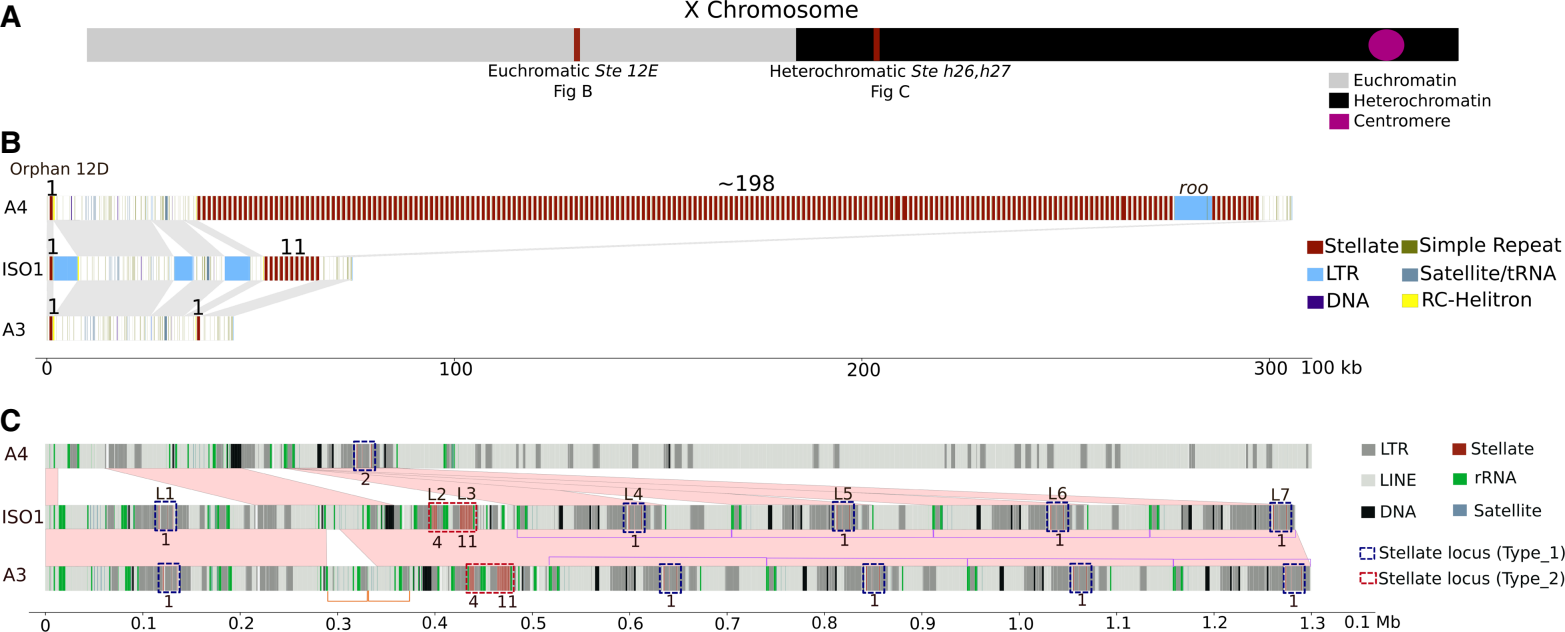
Maps and structural comparison of the euchromatic and heterochromatic *Stellate* gene clusters. (*A*) Schematic illustration of the location of the *Stellate* clusters relative to the rest of the X Chromosome. (*B*) Overview of the structure of the euchromatic *Stellate* locus. The numbers on the top represent full-length *Stellate* sequences in the tandem array. (*C*) Overview of the structure of the heterochromatic *Stellate* locus. Type_1 and Type_2 loci are marked by labels on the top in iso-1. The numbers on the bottom represent full-length *Stellate* sequences in the particular locus. The light pink color parallelograms represent syntenic sequences (drawn by eye). Purple half-rectangles represent the ∼200-kb tandem segmental duplicates harboring some of Type_1 loci.

Our assemblies permitted us to analyze the copy number of both X-linked *Stellate* clusters (Fig. 6A). In the euchromatic *Stellate* cluster spanning bands 12E1–12E2, the three strains iso-1, A4, and A3 exhibited three very different configurations, with the most notable variation on the proximal end of the cluster (Fig. 6B). Except for three LTR insertions in iso-1, the sequence flanking the distal side of the cluster is not structurally variable between strains, with all strains carrying a single copy of *Stellate* (called *Stellate 12D orphan*) preceding either ∼35 kb (in A3 and A4) or ∼52 kb (in iso-1) before the beginning of the *Stellate* cluster proper. The proximal end of the locus, on the other hand, shows significant variation. Our HiFi assemblies carry 11, 198, and one copy for iso-1, A4, and A3, respectively. Whereas our assembly of iso-1 bears 11 tandem copies in this region, the rel6 assembly of iso-1 shows 12 copies. The plot of HiFi read depth supports the locus structure of *Stellate* in our HiFi assembly, showing no hallmarks of collapse or misassembly in the read depth plot (Supplemental Fig. 18A). Additionally, the arrangements of repeat units in the cluster differ between iso-1 HiFi and iso-1 rel6, similar to our observation in the histone cluster (Supplemental Fig. 19). Although this may indicate an error in the public assembly based on an independent/older data set, it is also possible that this reflects the rapid evolution of copy number between the parental strain and its derivatives sequenced years later (Solares et al. 2018). Finally, in addition to carrying 198 tandem copies spanning ∼260 kb, the proximal portion in A4 carries a ∼9-kb *ROO* insertion. Like iso-1, the read depth plots for A4 and A3 show relatively uniform coverage (Supplemental Fig. 18B,C).

#### Heterochromatic Stellate cluster

The heterochromatic *Stellate* cluster (*hetSte*) spans the h26 and h27 regions of the X Chromosome (Tulin et al. 1997). This cluster is part of the newly assembled X-linked sequence (see Fig. 2B,C). We distinguish between two major cluster subtypes containing copies of *Stellate*. The first (labeled Type_1) contains a single full-length copy of *Stellate*. It appears to be derived from a tandem array of four 1150-type repeat copies that acquired various insertions and deletions, leaving only one *Stellate* copy intact. The second (Type_2) contributes the most copies to the X and comes in two forms, one with four and one with 11 intact tandem copies (Fig. 6C).

The iso-1 strain carries five loci of the Type_1 subtype, including the most distal locus (L1) and the four most′ proximal loci (L4–L7) (Fig. 6C). L1 contains four copies of *Stellate* (three partial and one complete). In addition to *Stellate*, this sequence contains insertions of *LINE/R1*, *MDG1-LTR*, and *NINJA-LTR* (Supplemental Fig. 20). In this L1 variant of the Type_1 subtype, the full-length *Stellate* copy is present in the first unit. The L4, L5, L6, and L7 loci are also Type_1 derivatives, but the first unit is missing, and the second element lacks the small *LINE/R1* sequence (Supplemental Fig. 20). In contrast to L1, for L4–L7, the full copy of *Stellate* is present in the third unit. All Type_1 sequences possess a *NINJA* insertion at the 90th position of the third unit, although the L1 locus also carries a partial deletion of *Stellate* (Supplemental Fig. 20). Thus, all Type_1 loci in iso-1 possess only one full-length *Stellate* repeat. The L4–L7 loci are within a series of tandem segmental duplicates, each ∼200 kb in length, and are present on the opposite strand relative to L1 (purple rectangles in Fig. 6C).

The L2 and L3 loci are Type_2 and comprise the main tandem *hetSte* clusters. They consist of five (four complete, one partial) and 12 (11 complete, one partial) *Stellate* repeat units, respectively (Fig. 6C). Each locus comprises a tandem *Stellate* cluster flanked on one side by *MICROPIA* and on the other by *BATUMI* TEs and a mix of *R1/R2* transposons and rDNA sequences, which we abbreviate as mSbr. The L2 and L3 loci are adjacent to each other in an inverted tandem orientation (i.e., [mSbr][rb′S′m′], where the ′ indicates the opposite orientation) (Supplemental Fig. 21). As a consequence, the *Stellate* clusters in these loci are separated by ∼24 kb of sequences made up of *BATUMI*, rDNA, and *R1/R2* and are on opposite strands (Supplemental Fig. 21). Given that the first unit of both clusters contains the same partial deletion and that the clusters are on opposite strands, they are likely the result of tandem inversion duplicates (TIDs) (Reams and Roth 2015). Similarly, Type_1 loci L1 and L4, L5, L6, and L7 are also flanked by *BATUMI* and *R1* sequences and are on opposite strands, again suggesting that these are TIDs. A3 bears the same haplotype as iso-1 (except for a small duplication [orange rectangles in Fig. 6C]) in this region and hence has the same copy number. A4 exhibits a dramatically different structure in this region (as previously described in Fig. 2C) and has only one Type_1 locus similar to L4, L5, L6, and L7 (Fig. 6C). However, in this case, the first element is not deleted, so it has two full-length copies of the 1150-type repeat.

#### Comparative genomics of Stellate

To compare tandem units of *Stellate* clusters, we followed a similar approach to our comparisons of the histone cluster. Briefly, our approach relies on using anchors of homology based on diagnostic differences that make a repeat unit unique within a strain. In the euchromatic cluster, we identified only three landmark anchors in iso-1 and A4, two of which are the most distal and proximal units in both strains (Supplemental Fig. 22). The most proximal unit is missing the final 302 bp of the repeat. In A3, this is the only copy present. In the heterochromatic cluster, A3 and iso-1 exhibit the same number of repeats in the L2 and L3 loci. In fact, the L2 locus (five copies) exhibits the same structure in both strains (Supplemental Fig. 23A). The structure of the L3 locus (12 copies) is relatively stable. When identifying landmark anchors, we find that most units are reciprocally most related to another unit in the same position in the array, with one exception: a one-unit insertion/deletion in both homologous clusters and a single mismatched unit (Supplemental Fig. 23B).

A heterochromatin-enriched *Drosophila* assembly (Chang and Larracuente 2019) (now referred to as iso-1 HetEnr) also reconstructed heterochromatic *Stellate* sequences. Our L1 locus is found in Contig_135 (440 kb) of iso-1 HetEnr. What we call L2 and L3 bearing the five and 12 copy tandem arrays of *Stellate* are found in their Contig_5 (535 kb). The structure of L2 and L3 in iso-1 HetEnr matches exactly (to the base pair) with our HiFi assembly, with variants in both L2 and L3 having a one-to-one correspondence with *hetSte* variants in our HiFi assembly. In a small 35-kb contig of iso-1 HetEnr called Contig X_9, we also located sequences corresponding to our L4–L7 *Stellate* loci. The sequence in ContigX_9 spans the *Stellate* sequences and some flanking sequences on both sides of *Stellate* that correspond to a subset of the ∼200-kb unit of the four-unit tandem array in our assembly (Fig. 6C). We also inspected the tandem euchromatic cluster in iso-1 HetEnr and found a total of 12 copies. Ours represents a third assembly of the euchromatic cluster from the iso-1 strain, exhibiting a different structure compared to those in the Rel6 and our HiFi assemblies (Supplemental Fig. 19).

## Discussion

### Routine assembly leads to improved reference genomes

The assemblies of three near-isogenic *D. melanogaster* strains generated here are both more complete and contiguous than, and as accurate as, the community reference sequence iso-1. Notably, our assembly of a descendant of the reference strain iso-1 added ∼8.0 Mb of sequence to the autosomal and X Chromosome arms compared to the latest release (R6) of the reference genome assembly. This is almost half (∼47%) of ∼17 Mb added to those arms between the initial sequencing of the *D. melanogaster* genome (R1) (Adams et al. 2000) and the most recent version (R6) released in 2014 (Hoskins et al. 2015). R1 recovered ∼66% (116.2/175 Mb) of the estimated female genome (Bennett et al. 2003; Ellis et al. 2014) in chromosome arm scaffolds in the year 2000 (Adams et al. 2000). By 2014, Release 6 improved this by ∼10% to 76% (∼133.88 Mb) assembled in primary chromosome scaffolds. In comparison, we independently recovered ∼81% of the genome (141.9 Mb) in primary chromosome scaffolds, representing a ∼5% improvement. Importantly, these assemblies reliably render previously recalcitrant regions tractable for study.

The most substantial improvements to our assemblies have been in the repetitive regions of the genome, particularly the heterochromatin. For example, in iso-1, we extended the proximal end (i.e., the end nearest the centromere) of the X Chromosome, including capturing the initial ∼450 kb of the ∼2.8-Mb rDNA array consisting of 200–250 rDNA units (Bianciardi et al. 2012). In contrast, Release 6 (Hoskins et al. 2015) reports only ∼75 kb of auxiliary scaffolds for rDNA. We also identified 15.7 Mb of putative Y-linked sequence using routine assembly procedures. This exceeds the most complete published *D. melanogaster* Y assembly to date (Chang and Larracuente 2019) by ∼1 Mb, even though the latter employed a clever assembly approach based on enriching sequencing reads for heterochromatin followed by labor-intensive manual curation. However, even such advances will need to be integrated with existing knowledge to provide assemblies of the most challenging regions like the Y Chromosome (Carvalho et al. 2025).

### Newly assembled X-linked heterochromatic sequence

The rDNA locus and the surrounding sequences in X pericentric heterochromatin are highly repetitive and have resisted reconstruction at the sequence level. In arthropods, in addition to rRNA genes, the rDNA region harbors two TEs unique to the rDNA locus called LINE *R1* and *R2*. *R1* and *R2* possess specific insertion sites only in 28s rDNA of arthropods (Pérez-González and Eickbush 2002). *R1* is especially abundant in the distal region flanking the rDNA, occupying almost ∼50%–60% (Fig. 2C). Given its target site specificity, this many *R1* TEs in the region are unlikely to represent solely de novo insertion events. Instead, TEs can also form tandem repetitive arrays driven by unequal exchanges (McGurk and Barbash 2018). However, the newly assembled sequence does not resemble a standard homogenous tandem array, exhibiting a high degree of heterogeneity. In repetitive regions such as these, higher-order repeats (HORs) sometimes arise, driven by unequal exchanges (Suzuki et al. 2020). HORs may exhibit different organization either because they originated independently or because they acquired various deletions and insertions independently over time (such as other TEs). Our observations support a model where a region primarily containing *R1* tandem elements arose in the rDNA locus. Subsequent cycles of amplifications (and deletions) and the emergence of new HORs, driven by unequal exchanges, could result in the heterogeneous structure we observe today. The dot plots of the region (Supplemental Fig. 7) confirm the nature of tandem repetitive content reminiscent of dot plot patterns encountered for centromeric repeats containing HORs. In addition, the variable copy number segmental duplicates (Fig. 2A) likely represent larger-scale HOR structures. Using short-read data, McGurk and Barbash (2018) estimated copy number variation within the X-linked *R1* tandem array and used unique junctions to infer the heterogeneity of the array. However, with our fully resolved map of genetic variation within the locus, not only can we infer that these arrays are quite different, we can propose models to explain the mutational origins of the entire region. However, due to the absence of any apparent functional sequences, we are less confident about whether or not adaptation plays a role in the evolution of this region.

Due to the highly mutable nature of the structure of such loci (Nurminsky et al. 1994; Campbell and Eichler 2013) and the large effective population size of *D. melanogaster*, we expect a lot of genetic variation. Consequently, individual haplotypes are likely to exhibit large structural differences from one another, including sometimes dramatic variation in copy number. Thus, it is possible that the mutation underlying the ∼250-kb segment collapsed in our iso-1 assembly (Supplemental Fig. 8) is a very recent expansion. Such recent expansions would create two virtually identical segments, making them hard to resolve even with long, accurate reads (Porubsky et al. 2023).

### Structural variation in the histone cluster

#### Assembly of D. melanogaster histone cluster for iso-1, A4, and A3

Until recently, the precise primary sequence of the histone cluster was virtually impossible to reconstruct. Although the histone cluster is largely homogeneous, some variation does exist between the units (Figs. 3–5). Despite this variation, the long stretches of virtually identical repeats within the cluster have rendered the histone locus a substantial challenge to assemble. Until now, the most reliable DNA sequencing-based model of the histone cluster was painstakingly reconstructed via a bespoke assembly approach that takes advantage of machine learning to assemble older and noisy long-read data (Bongartz and Schloissnig 2019). With recent improvements to error rates in long-read sequencing (e.g., from one error in 10 bp to one error in 1000 bp per sequencing read [Marx 2023]), we can now accurately reconstruct the histone cluster with routine application of standard assembly approaches (Supplemental Figs. 10, 11, 13, 14). Our results were virtually identical to those of Bongartz and Schloissnig (2019) except for an apparent disagreement in the most proximal two units. Because we identified individual reads supporting our assembly model, this difference is due to either assembly error in the iso-1-BS genome or a polymorphism segregating in the strain, an issue also raised by Bongartz and Schloissnig. Such remarkable similarity between independent sequencing data sets and alternate assembly approaches gives us confidence in our assembly and any analyses we do further downstream. Even though we did not have to resort to novel assembly software to solve the locus, our approach did require targeted assembly of the histone locus with parameters appropriate for extremely repetitive sequences (see Methods) and was not successful for every data set (Supplemental Figs. 15, 16). These improvements in the reconstruction of the histone cluster enable an unprecedented view into the structural variability of this functionally important locus.

#### Dramatic variation in copy number and organization of histone repeat units

Our de novo assemblies indicated copy numbers ranging from as few as 111 copies in A4 to between 134 and 197 units in A3. For A3, both estimates are substantially higher than expected from previous surveys of the histone locus, which typically converges on a copy number of roughly 110 copies (Lifton et al. 1978; Strausbaugh and Weinberg 1982). These results suggest that dramatic copy number variation in the histone cluster may be common. To independently validate this, we used Illumina read coverage in the *Drosophila* Global Diversity Lines (Grenier et al. 2015), which exhibits a median of 104 copies, with half between 90 and 112 copies, in line with other estimates. However, in the extremes, natural copy number varied over fivefold, ranging from 29 to 146. Conservatively, this is strong evidence for severalfold natural copy number variation in the histone locus. Despite this wide variation (particularly the low copy number of 29), our observations are consistent with published experimental evidence in histone knockout lines demonstrating that transgene copy numbers as low as eight are viable and as low as 20 restores near wild-type fitness (Zhang et al. 2019). Additionally, the RNA or protein levels between flies with either 12 or 20 copies show no significant difference (Zhang et al. 2019), indicating dosage compensation mechanisms are active in histone protein production, possibly leading to relaxed selection on copy number.

Among our assemblies, the organization of the histone locus is also quite variable (Fig. 4A,B). To characterize this dramatic variation and elucidate the dynamics of the array, we surveyed the organization of the locus using several perspectives. These relied on the relative position and ordering of reference points marked by unique TE insertions, repeat unit types (i.e., 5-kb and 4.8-kb), repeat unit subtypes (5053 and 5053 subtypes of the 5-kb type), and “landmark anchors” (i.e., unique units within strains shared between strains) (Fig. 4). Whereas, at a high level, unit types and subtypes are distributed similarly across the locus, their specific copy number, pattern of interleaving with each other, and spacing features are variable, requiring many mutations to explain. Even more organizational diversity is evident when we consider the structure of the locus from the perspective of unique TE insertions and landmark anchors (Fig. 4A). Notably, we observe that certain pairs of anchors can swap their relative orders (see the crossing of dotted lines in Fig. 4A). Such variation is incompatible with simple expansion or contraction, requiring multiple mutational steps. Given that gene conversion and repeat expansion and contraction are ubiquitous in these regions, this opens up several possibilities. One scenario is the gene conversion of one anchor followed by the loss of the first copy (either through another gene conversion event or through deletion). Another possibility involves a duplication followed by at least two deletions (Supplemental Fig. 24). The common thread among these and other possibilities is that the differences between haplotypes require many events and could employ many mechanisms.

#### Concerted evolution in the histone cluster is primarily driven by intrachromosomal recombination

We show that the histone cluster is extremely homogeneous, exhibiting at most a median difference between any two units of the array of 0.378%–0.417% or 0.416%–0.457%, within and between stains, respectively. However, comparisons between *D. melanogaster* and *D. simulans* show a 6.7% divergence for aligned nucleotides or about 9.2% when indels are counted. This is 5.6- to 7.7-fold higher than the most divergent comparison (1.2%) within *D. melanogaster*, a classic signal of strong concerted evolution (Matsuo and Yamazaki 1989; Hickey et al. 1991). These results generalize previous conclusions concerning *H3* variation between these species (Matsuo and Yamazaki 1989), which showed an 8.5-fold difference. The repeat units in the histone locus exhibit stronger homogenization between units that are closer to one another, regardless of whether they inhabit the same strain (Fig. 4C). Homogenization within strains is higher than between, although only within about 30 units (i.e., ∼150 kb), after which the genetic variation between repeat units is determined primarily by their relative position in the array rather than whether they are found in the same strain (Fig. 4C).

One surprising observation is the near absence of evidence of meiotic crossing over in the segments surrounding the histone cluster, although not in flanking regions. For 145 of the 147 DGN strains, SNPs immediately flanking either side of the histone cluster are in complete LD (Fig. 5A). Although both exceptions are compatible with crossover events, they are also compatible with gene conversion events. In both cases, the haplotype switch is embedded in a region of otherwise extremely elevated LD extending over hundreds of kilobases (Fig. 5D). This makes it very plausible that the level of recombination we observe is a result of gene conversion rather than crossing over. Whereas the “centromere effect” (reduced crossing over near centromeres) has been known since 1932 (Beadle 1932), and recent studies observed decreased crossing over near the histone cluster (Comeron et al. 2012; Hartmann et al. 2019), our study is the first to use such high-resolution variation data to document a complete absence of crossing over specifically within the histone cluster that does not extend to more proximal regions.

Population samples of fully resolved tandem arrays reveal patterns of variation we can use to infer the mechanisms responsible for this concerted evolution in such tandem clusters. Our discussion turns on three major observations: (1) there is limited evidence for crossing over across the histone cluster; (2) similar repeat types cluster together within strains, but different strains have different arrangements of such clusters; and (3) homogenization tracts are locally concentrated. Collectively, this suggests that the rate of intrachromosomal recombination is significantly higher than the rate of recombination between homologous chromosomes (interchromosomal recombination) (Supplemental Fig. 25). To support this suggestion, we surveyed previously reported mechanisms thought to be responsible for inter- and intrachromosomal recombination below.

Interchromosomal recombination may occur as either gene conversion or crossing over during meiosis. Although gene conversion in tandem arrays is hard to assay, the rate of meiotic crossovers can be estimated using SNP markers flanking the arrays. Figure 6 shows very little decay of linkage disequilibrium across the span of the histone cluster. This indicates little or no meiotic crossing over happening in the vicinity of the histone locus. This is consistent with previous observations in tandemly repeated loci like the human rDNA array (Seperack et al. 1988) and U2 snRNA array (Liao et al. 1997), where nearly complete linkage disequilibrium was observed across these arrays. Nevertheless, we demonstrated that substantial variation in the structure of tandem repeats exists within the histone locus itself. Our interpretation of these observations is that concerted evolution of this tandem cluster is primarily driven by intrachromosomal rather than interchromosomal recombination. Our conclusions are consistent with those of Schlötterer and Tautz (1994), who analyzed the distribution of polymorphic sites in ITS of the rDNA in *Drosophila* populations. Analogous conclusions based on human variation data have also been made for the rDNA (Seperack et al. 1988; Hori et al. 2021) and U2 snRNA (Liao et al. 1997). Because the histone cluster is not considered heterochromatin, another feature of the locus must be responsible for our observations, like perhaps its tandem repetitive nature.

Concerted evolution is generally thought to be the result of DNA recombination mechanisms, primarily gene conversion and/or crossing over. Conversion is a byproduct of recombinational repair mechanisms. DNA damage, especially double-stranded breaks (DSBs) are deleterious to the cell and can lead to genomic instability if not repaired. DSBs can be repaired by both homologous recombination (HR) and nonhomologous (NHEJ) mechanisms. HR results in a unidirectional transfer of information from one DNA molecule to another (i.e., it is a type of gene conversion) and/or reciprocal exchange of information between two DNA molecules (e.g., what occurs in crossing over). In HR repair, the homologous template can be either the homologous chromosome (interchromosomal), sister chromatid (intrachromosomal), or, in case of repetitive sequence, nonhomologous repeats on the same molecule (also intrachromosomal).

When DSBs occur during the G2/S in mitotically dividing cells, the sister chromatid is the preferred molecule for repair in yeast (Kadyk and Hartwell 1992) and mammalian cells (Johnson and Jasin 2000). Sister chromatid repair might involve both crossover events and/or gene conversion. Unequal sister chromatid exchange (USCE) (Tartof 1974; Axelrod et al. 1994) is a type of crossing over that can create copy number changes and duplications and deletions in tandem repeats (like those we see in the histone cluster). The alternative, gene conversion, is often associated with relatively small tracts on the order of a few hundred bases (<200 bp in yeast and <100 bp in mammalian cells [Chandramouly et al. 2013]), which is likely too short to explain the bulk of our observations, which happen on the scale of many thousands of bases. However, there is another class of gene conversion events known as long tract gene conversion (LTGC), which extend for several kilobases (e.g., >15 kb and can be 100 kb long or more [Stewart et al. 2021]). LTGC can generate amplifications (Johnson and Jasin 2000; Puget et al. 2005), which is consistent with events in the histone locus that may span one or more repeat units of ∼5 kb.

When intramolecular recombination (i.e., intramolecular crossing over or gene conversion) occurs, it is particularly likely in tandem repeats. Intramolecular crossing over results in formation of two products: an extra circular DNA containing multiples of repeat units and the DNA molecule having some units deleted. Evidence for extrachromosomal DNA consisting of tandem arrays such as histone, rDNA, *Stellate*, and *Su(Ste)* has been found in *Drosophila melanogaster* (Cohen et al. 2003). Whereas intramolecular gene conversion could, in principle, homogenize tandem repeat copies, direct observations of this are limited in *Drosophila*.

Our observations do not allow us to distinguish which particular intrachromosomal mechanism is primarily responsible for homogenizing repeat copies, although none are mutually exclusive and could all contribute. Mitotic processes driving concerted evolution such as USCE, LTGC, and intramolecular crossing-over can generate duplications and deletions and thereby participate in the rapid turnover of units and significant copy number distribution that we observe. Consequently, when considering mechanisms of concerted evolution, it is crucial to examine not only meiotic events but also mitotic processes occurring in germ cell precursors. The differences between the organization of haplotypes suggest to us that crossing over between different haplotypes during meiosis would seldom lead to the kind of duplication patterns we observe. Combined with the observation that crossing-over is rare or absent across the locus, the alternative is intrachromosomal crossing-over. If the rate of interchromosomal recombination were comparable to the intrachromosomal rate, we would predict that haplotype-specific patterns of duplication generated through intrachromosomal mechanisms would become unlinked. Although intrachromosomal mechanisms likely dominate the evolution of the tandem histone locus, in the absence of nonmutational forces (e.g., meiotic drive), interchromosomal recombination must occur at a sufficient rate to explain why repeat copies in *D. melanogaster* are more similar to one another compared to repeat units in *D. simulans* (sister taxa) (Liao 1999). If there is absolutely no genetic exchange in such loci within the population (i.e., no interchromosomal recombination), every chromosomal lineage would evolve independently, leading to large differences in repeat copies between haplotypes even within species. However, this is not what we observe in concerted evolution. The copies within species are still more similar than copies between sister taxa. Therefore, sufficient levels of genetic exchange must happen between haplotypes within a species. Demonstration of limited to no crossing-over between two chromosomes suggests that occasional gene conversion (without reciprocal exchange) between two different haplotypes may be one way through which interchromosomal recombination is mediated (Liao et al. 1997).

### Structural variation of the X-linked *Stellate* clusters

Our results show that most copy number variation for *Stellate* stems from the X-linked euchromatic cluster (*euSte*), confirming the conclusion of Palumbo et al. (1994). Indeed, if we exclude the orphan copy in 12D, we observe variation from as few as one copy in A3 up to ∼198 in A4, with iso-1 harboring 11 copies (Fig. 6B). Our estimate from iso-1 is consistent with estimates from other assemblies of the strain, including iso-1 HetEnr and iso-1 Rel6, exhibiting 12 copies. However, all versions exhibit differences in their organization (Supplemental Fig. 19). Such differences can be attributed to mutational mechanisms active in tandem arrays, although misassembly of either assembly in our comparison could also be the cause. These mutation types are special in that they may accumulate relatively rapidly and result in dramatic changes in locus organization, especially compared to our low expectations of SNP and small indel mutation rates of ∼1 × 10^−9^. Another cluster of *Stellate* genes resides in the heterochromatin (*hetSte*) of the X Chromosome. We classified two major cluster subtypes of *hetSte* based on the organization of *Stellate* repeat units: Type_1 and Type_2. Type_1 is derived from a four-unit tandem array, which acquired various indels, leaving only one or two *Stellate* copies intact. The homogenous tandem arrays of *hetSte* repeat units were designated as Type_2. Because Type_1 loci have only one or two full-length copies in the three strains we studied, they might not be the main contributors to copy number variation in *hetSte*. Type_2 loci with their tandem arrangements might be responsible for the lion's share of variation in *hetSte*. Type_2 loci (L2, L3) exhibit very few differences in arrangement between iso-1 HiFi and A3 and no difference between our iso-1 HiFi and iso-1 HetEnr assemblies. This contrasts with the euchromatic cluster, where even the same strain (iso-1) exhibits rapid turnover of these units for a similar number of repeat units. Failure to find many differences between A3 and iso-1 indicates that the tandem *hetSte* evolves slowly, which might explain the limited variability in this cluster.

Previous studies of *hetSte* have inferred independent origins of the euchromatic and the heterochromatic *Ste* clusters (Chang and Larracuente 2019; Adashev et al. 2021). We used fully resolved sequences of *hetSte* to show that all Type_1 and Type_2 *hetSte* loci can trace their origin to a single ancestral array, duplicated either through tandem inversion duplicates or by being part of larger segmental duplicates. These segmental duplicates are examples of the HOR structures we described for the rDNA distal X-linked locus. Upon further inspection, we find that even Type_1 and Type_2 have common flanking sequences derived from *BATUMI* TEs on one end, pointing to a possible single origin of the heterochromatic clusters. Similarly, the first *Stellate* orphan sits on the opposite strand with respect to the main *euSte* tandem cluster. Both loci have fragments of *DINE-1* TEs plus a very small partial *Stellate* fragment flanking one of the sides. This again suggests that the two loci might have originated from a TID event, and subsequently, one of them amplified. This is similar to the conclusions reached by Kogan et al. (2012) (although they implicate *DINE-1* as a driver of duplication). Hence, our analyses based on the detailed maps of these regions suggest that both the euchromatic and the heterochromatic clusters have originated via single independent copying events and were subsequently amplified.

#### Concerted evolution in the euchromatic Stellate cluster

The *euSte* cluster exhibited extreme variation in copy number (from one to 198 copies), prompting further investigation into concerted evolution at the *Ste* clusters. The relationship between physical and molecular distance between repeat units for A4 *euSte* shows a different pattern than the histone cluster. Adjacent copies are not more similar than separated copies (Supplemental Fig. 26A). Several obvious explanations fall short of explaining this pattern. Rapid array-wide homogenization could lead to such a uniform pattern. However, we do not see any significant differences for within- versus between-haplotype comparisons, as expected in the case of rapid homogenization (Supplemental Fig. 26B). Although the recent emergence of the *Stellate* array compared to the histone array would have afforded less time to accumulate variation between *Stellate* units than histone units, we nevertheless find similar levels of variation (median of all pairwise distances) in *Stellate* (0.0031) and histone (∼0.004) clusters. In contrast to the histone cluster, if the relative rates of intra- versus interchromosomal exchanges are similar in the *Stellate* cluster, we also might see no distance-dependent effect. Relevant factors include the size of a single unit, the total size of the entire cluster, the range of localized interactions for gene conversion/duplication events, and the median length of such events. These interdependent factors taken together can also contribute to the observed difference between the histone and *Stellate* arrays. The availability of more full-length *euSte* clusters is required to address these questions, and it is left for future work.

### Persistent limitations in mapping the dark matter of the repetitive genome

Whereas the work we report represents substantial progress, the heterochromatic regions we discussed illustrate some of the challenges that persist. Indeed, in the human telomere-to-telomere project, the rDNA is one of the only remaining frontiers where sequencing does not solve the region (Nurk et al. 2022). In our case, in addition to not assembling through the entire rDNA array, there are clear problems both adjacent to and inside the rDNA region. Specifically, the elevated sequence read coverage in that region strongly suggests that it has been collapsed in the assembly (Supplemental Fig. 8). We also recovered an additional 6.4 Mb of rDNA sequences in our iso-1 assembly; 4.26 Mb was assigned to X, 1.48 Mb to Y, and 0.67 Mb was left unassigned. We could not order and orient these sequences, so they remain spread across 29 contigs, complicating downstream variation analyses. The assembly and scaffolding of the entire Y Chromosome face similar challenges (Carvalho et al. 2025). Whereas the iso-1 assembly did largely recapitulate results from a specialized approach employing targeted enrichment of heterochromatin for reconstructing the Y Chromosome (Supplemental Fig. 6; Chang and Larracuente 2019), it was nevertheless spread across 63 contigs that cannot be scaffolded through assembly alone. Finally, these assemblies have yet to span centromeres, likely due to how they are embedded in or flanked by seas of satellite repeats (Supplemental Fig. S1 in Hoskins et al. 2015). These limitations highlight the challenges we must overcome to resolve the most recalcitrant regions of the genome. Although recent technical advances permit us to recover far more of the repetitive genome than previously, much of what we recover remains fragmented, effectively stymieing any study of structural variation. Even were we to employ the painstaking methods required to construct reasonable models of the genome structure, such progress would be incremental relative to previous results. A robust solution to these problems would entail developing approaches that can be used routinely to assemble and compare truly gapless or telomere-to-telomere genome assemblies of a population sample. The emergence of telomere-to-telomere genome assembly and pangenome approaches (Taylor et al. 2024) brings us close to routine assembly and comparison of truly gapless genome assemblies in populations. However, our preliminary analyses suggest that achieving this goal in *D. melanogaster* will require a multipronged strategy to overcome specific technical hurdles, including addressing sequencing biases against particular satellite repeats and potentially using alternative tissue sources or complementary sequencing technologies (Supplemental Note 1, Section 4). Until these challenges are solved, the repetitive dark matter of genomes will continue to limit our understanding of structural variation and genome evolution.

## Methods

### DNA extraction and sequencing

High molecular weight (HMW) DNA was extracted from ∼200 iso-1, A4, and A3 male adult flies using Qiagen Blood and Cell Culture Midi kit following Chakraborty et al. (2016). HMW DNA was sheared using gTUBE (Covaris), and HiFi libraries were prepared using the SMRTbell Express Template Prep kit 2.0 (Pacific Biosciences) following the manufacturer's recommendations. The libraries were further size-selected and sequenced on the Pacific Biosciences Sequel II platform at UC Irvine Genomics High-Throughput Facility. CCS reads (ccs.bam*)* were generated from the PacBio subreads.bam file using ccs (v6.2.0). CCS reads with QV ≥ 0.99 were labeled as HiFi reads. PCR-free Illumina libraries were generated from an independent DNA extraction from the strains used for HiFi reads. The libraries were sequenced on NovaSeq 6000 platform at Novogene to generate 150-bp paired-end (2 × 150) reads.

### Genome assembly

The HiFi reads longer than 6 kb were assembled using hifiasm v0.16.1 (Cheng et al. 2021). The primary assembly from hifiasm was designated as our primary contig-level assembly. We removed contigs mapping to the mitochondrial genome. We aligned the first 25 kb of each contig to the nr database using BLASTN to identify the contigs derived from non-*Drosophila* sources, particularly commensal microbes (Staubach et al. 2013). We then removed contigs that matched such sequences.

### Comparative and Hi-C–based scaffolding

Two lines of evidence were employed to scaffold our iso-1 decontaminated contig assembly: reference-assisted scaffolding and Hi-C scaffolding. For reference-assisted scaffolding, the contig assembly was mapped to iso-1 FlyBase Release 6.36 using minimap2 v2.24 (Li 2018). The resulting alignment PAF file was uploaded to the D-GENIES web server (Cabanettes and Klopp 2018) to visualize the order and orientation of the contigs with respect to the FlyBase chromosome arm scaffolds using a dot plot. This represents our primary scaffolding approach. We also used a complementary scaffolding approach based on a Hi-C contact map to incorporate sequences absent from the FlyBase reference and represented ambiguously in the dot plot. For Hi-C–based scaffolding, we mapped the Hi-C reads (obtained from the NCBI Sequence Read Archive [SRA; https://www.ncbi.nlm.nih.gov/sra] under accession numbers SRR5206663-65) from male embryos (Schauer et al. 2017) to our contig assembly and processed using the Juicer v1.6 pipeline (Durand et al. 2016). Subsequently, 3d-dna v201008 (Dudchenko et al. 2017) was used to perform scaffolding. Additionally, the Hi-C contact map was compared to the D-GENIES dot plot to resolve ordering discrepancies, with reference-guided ordering given priority over Hi-C. The information from both synteny and Hi-C were integrated to generate the final scaffolded reference using a custom script. The final Hi-C contact map for iso-1 can be found in Supplemental Figure 5. A4 and A3 decontaminated contig assemblies were mapped to iso-1 scaffolded HiFi assembly using minimap2 v2.24 (Li 2018). Reference-assisted scaffolding was performed using the procedure described above.

### Quality control and heterozygosity analysis

We calculated BUSCO and consensus quality (QV) to test the quality of our assembled genomes. The BUSCO scores for the decontaminated contig assemblies were calculated using compleasm v0.2.2 (Supplemental Table 5; Huang and Li 2023). Merqury v1.3 (Rhie et al. 2020) was used to obtain Phred QV score and *k*-mer completeness for the final scaffolded assemblies (Supplemental Table 5). The Illumina data generated for the three strains was used during Merqury evaluation. The QV score reported here is the average of the major chromosomal arms. Heterozygosity analysis was performed using GenomeScope 2.0 (Ranallo-Benavidez et al. 2020). We tweaked the standard approach to derive heterozygosity estimates for our data sets. Because we had sequenced the heterogametic sex (males), GenomeScope interpreted the haploid peak (from X and Y Chromosomes) as the heterozygous peak. Thus, we employed only reads derived from autosomes. HiFi reads were mapped to the scaffolded genome using minimap2 v2.25 (Li 2021), and reads mapping to autosomes were extracted. This subset of reads was further used to count *k*-mers (using Jellyfish v2.3.0 [Marçais and Kingsford 2011]) and obtain a GenomeScope profile (Supplemental Figs. 2–4).

### Identification of Y-linked contigs

We employed a modified approach from Chang and Larracuente (2019) to identify Y-linked contigs. Male and female PCR-free Illumina reads (SRA; SRR6399448-49) were mapped to the contig assemblies using BWA-MEM v0.17.7 (Li 2013). SAMtools coverage v1.16 (-q 20) was used to calculate coverage statistics for each contig in the assembly. Among the various metrics reported by SAMtools coverage (e.g., *covbases, coverage, meandepth*), we used the *coverage* metric as it provides the percentage of the contig covered by reads. We used the ratio of male to female *coverage* to determine the sex-linkage of the contigs. For autosomal contigs and X, this ratio should be ∼1, but for Y-linked contigs, the ratio should be significantly greater than 1. We classified any contig with *coverage* ratio >2 as Y-linked (excluding contigs with less than five reads mapped). Contigs in females that have 0 *coverage* but have *coverage* >0 in males were also classified as Y-linked. For contigs (such as those consisting entirely of simple sequence repeats), which have 0 *coverage* in both male and female when a filter of MAPQ > 20 is applied, we applied the procedure with no MAPQ cutoff. Such contigs with 0 *coverage* in females and nonzero *coverage* in males were classified as Y-linked.

### Synteny, repeat content, and depth analysis

Synteny analysis was performed using D-Genies (Cabanettes and Klopp 2018). The genomes were aligned using minimap2 v2.24 to obtain a PAF file. Index files for the genomes were created by a Python script provided by the D-Genies authors. The resultant PAF file and index files were uploaded to the D-Genies server (https://dgenies.toulouse.inra.fr/). The online server is an interactive way to visualize synteny using a dot plot and generate static dot plot images.

RepeatMasker v4.1.2-p1 (Smit et al. 2015) was used to identify repeats in our assemblies. The default Dfam 3.3 curated library (Storer et al. 2021) was augmented with a few sequences relevant to our study, such as *Stellate*, histone, and rDNA, to obtain a custom repeat library. This custom library was used to identify repeats in our assemblies. The extra sequences appended can be found on the GitHub page.

We used depth analysis to verify the integrity of an assembled sequence. HiFi reads were mapped to the assembly using minimap2 v2.24, and SAMtools depth v1.6 (Danecek et al. 2021) was used to calculate per-base depth for the locus. An Rscript was used to plot and visualize the depth across the locus.

### Targeted assembly of the histone cluster

HiFi reads were mapped to the entire contig assembly using minimap2 v2.24. The list of reads mapping to the histone locus +10 kb flank was obtained using a SAMtools view. seqtk v1.3-r116-dirty was used to extract the sequences in the list file. These reads were assembled using hifiasm v0.16.1 with repeat sensitive and targeted assembly parameters (-D 10 -hg-size <estimated_size_locus>). The largest contig from the primary assembly (*.p_ctg.fasta) output was considered as our histone locus. In addition, the unitig graph (*.p_utg.gfa) from both the default and targeted assemblies was visualized in Bandage v0.9.0 (Wick et al. 2015) in order to investigate the structure of the locus further (Supplemental Figs. 11, 14, 16). For A3, we also assembled the longest 40× reads, but both approaches failed to assemble the entire cluster. Thus, we decided only to consider the well-resolved parts of the 40× unitig graph for our final assembly (Supplemental Fig. 16). The nodes in the path (shown by red lines) were merged in Bandage v0.9.0, and their FASTA sequences were written to the disk. The sequences were finally scaffolded together in proper order and orientation using our scaffolding method.

### Inferring histone copy number from short-read data and dPCR

The histone locus in the iso-1 HiFi scaffolded assembly was masked. A single histone unit (5-kb type) was added as a separate contig to the assembly. GDL (Grenier et al. 2015) short-read Illumina data were mapped to the modified reference using BWA-MEM v0.7.17 (Li 2013). SAMtools depth v1.6 (-aa -J -s) was used to calculate the per-base depth for the histone contig and the 2L arm. The ratio (Histone_contig/2L) of the median per-base depth was inferred to be the histone copy number for that particular strain. The SRR IDs of the GDL strains used here are provided in Supplemental File 2. Azenta Life Sciences was contracted to perform a digital PCR (dPCR) assay. Independent DNA extractions were generated from the three strains in this study and six extreme outlier lines from the GDL data set and submitted to the company. Azenta Life Sciences designed primers based on the coding sequence (CDS) of the *His3* (target) and *RpS3* (reference) genes and conducted the dPCR experiment. Subsequently, they performed the data analysis and reported the copy number values (Supplemental File 6).

### Analysis of the histone locus using phylogenetic methods

The beginning and start sequences of a single histone unit were identified. RepeatMasker v4.1.2-p1 was used to find their locations in all the histone arrays using these sequences. The individual units from each of the histone loci were extracted using the RepeatMasker output file. The TE-containing units were removed, and all units from the two strains were merged into a single FASTA file for pairwise comparison. The combined FASTA file was imported into MEGA v11.0.13 (Tamura et al. 2021), and a multiple sequence alignment (MSA) was performed using MUSCLE (Edgar 2004). This MSA was used as input for building a phylogenetic tree. A neighbor-joining tree (500 bootstraps) was constructed using default parameters. Finally, a 50% bootstrap consensus tree was obtained. If only two units, one from each strain, clustered together in the consensus tree, they were marked as putative anchors. This procedure was followed for all pairwise comparisons. The blue boxes highlight such pairs in the pairwise phylogenetic trees (Supplemental File 3). To identify closely related repeat units (Fig. 4B), we looked at clades containing units from both strains in each pairwise comparison. We correlated results across all the pairwise comparisons to obtain an overall pattern for the three strains. The red, purple, and green boxes highlight such clades (Supplemental File 3). The colors here correspond to the ones used in Figure 4B. The molecular distances used to plot Figure 4C were obtained using MEGA using default parameters.

### Linkage disequilibrium analysis in and around the histone locus

For the linkage disequilibrium analysis, we employed a sequencing data set (DGN) derived from a highly diverse population of *D. melanogaster* from its ancestral range (Lack et al. 2015) using haploid embryos (Langley et al. 2011) to remove complications related to heterozygosity. Illumina short-reads were trimmed using TrimGalore v0.6.10 (https://github.com/FelixKrueger/TrimGalore). The trimmed reads were mapped to the iso-1 HiFi scaffolded assembly, with its histone locus masked, using BWA-MEM v0.7.17. Variants were called using octopus v0.7.4 (Cooke et al. 2021) for a ∼1-Mb region, flanking the histone cluster for each individual strain. Joint genotyping was performed using previously generated bcf files for 147 strains. A few filters were applied to the jointly called VCF using BCFtools v1.7 (Danecek et al. 2021). Genotypes that were not labeled “PASS” were converted to “no calls”; only biallelic sites were further considered; heterozygous calls were converted to “no calls” because we are dealing with haploid embryos. Finally, sites with missing genotype calls >5% or having minor allele frequency <0.05 were filtered out. This VCF file was imported into IGV (Thorvaldsdóttir et al. 2013) for further analysis. We used PLINK v1.90b7 (Chang et al. 2015) with biallelic SNPs to calculate the pairwise LD (*r*^2^) matrix. The SRR IDs of the DGN strains used here are provided in Supplemental File 2.

### Analysis of the *Stellate* locus using phylogenetic methods

The same methods used to analyze the histone locus were applied to investigate the *Stellate* locus. The blue boxes represent the anchors identified for the euchromatic and heterochromatic locus for iso-1 HiFi, A4 HiFi, and A3 HiFi (Supplemental File 4). A similar procedure was followed for comparing the euchromatic and heterochromatic *Stellate* locus from three iso-1 assemblies (Supplemental File 5).

## Data access

The raw sequencing data and genome assemblies generated in this study have been submitted to the NCBI BioProject database (https://www.ncbi.nlm.nih.gov/bioproject/) under accession number PRJNA1122219. All scripts, analysis pipelines, and relevant data files are deposited in GitHub (https://github.com/harsh-shukla/Dmel_HiFi_Asm_variation) and are also available as Supplemental Code.

## Supplemental Material

Supplement 1

Supplement 2

Supplement 3

Supplement 4

Supplement 5

Supplement 6

Supplement 7

Supplement 8

Supplement 9

Supplement 10
