# Supplementary material for: Genetic variation in recalcitrant repetitive regions of the Drosophila melanogaster genome
Source: Genome Res. 2025 Sep;35(9):2023–40. doi: 10.1101/gr.280728.125 (PMC12400953; doi:10.1101/gr.280728.125)
Supplement: Supplement 1 [file Supplemental_Figures.pdf]

Supplemental Figures For:

Genetic variation in recalcitrant repetitive regions of the *Drosophila melanogaster* genome

Harsh G. Shukla, Mahul Chakraborty, J.J. Emerson

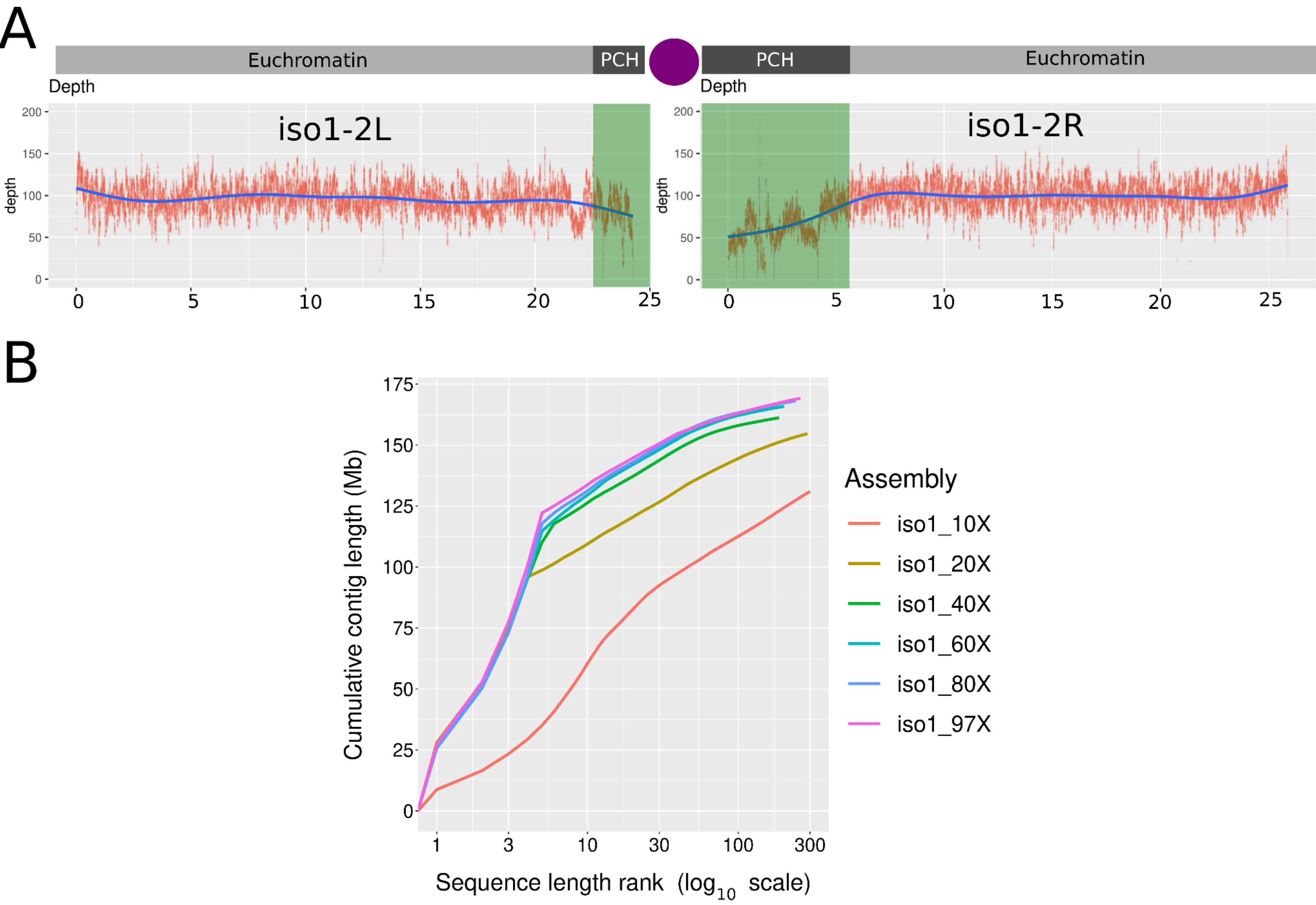

Supplemental Fig 1. (A) Depth plots for arms 2L and 2R for iso-1. The Y-axis is per base depth and X-axis is genomic location (in Mb). The blue line is the fit. The green box highlights the reduced depth in heterochromatic (PCH) regions. **(B)** Cumulative length plot for assembly contigs for various depths. We performed subsampling analysis on our iso-1 dataset (~97× coverage) to assess the impact of read depth. Assemblies with 40×-97× coverage produced comparable results for euchromatic regions. However, lower coverages led to shorter heterochromatic contigs and reduced assembly size, likely due to insufficient coverage in these repetitive regions. Heterochromatic regions show reduced coverage perhaps due to under-replication in some adult fly tissues (Fig A, green highlighted areas). This variation in coverage affects assembly quality in these regions at lower depths. For 20×, the euchromatic arms do appear to be full length except for the X Chromosome. We sequenced males, as a result the X has effectively half the genome wide coverage. But the heterochromatic sequences are quite fragmented. The 10× assembly is worse, exhibiting multiple breaks across all the arms in both euchromatin and heterochromatin.

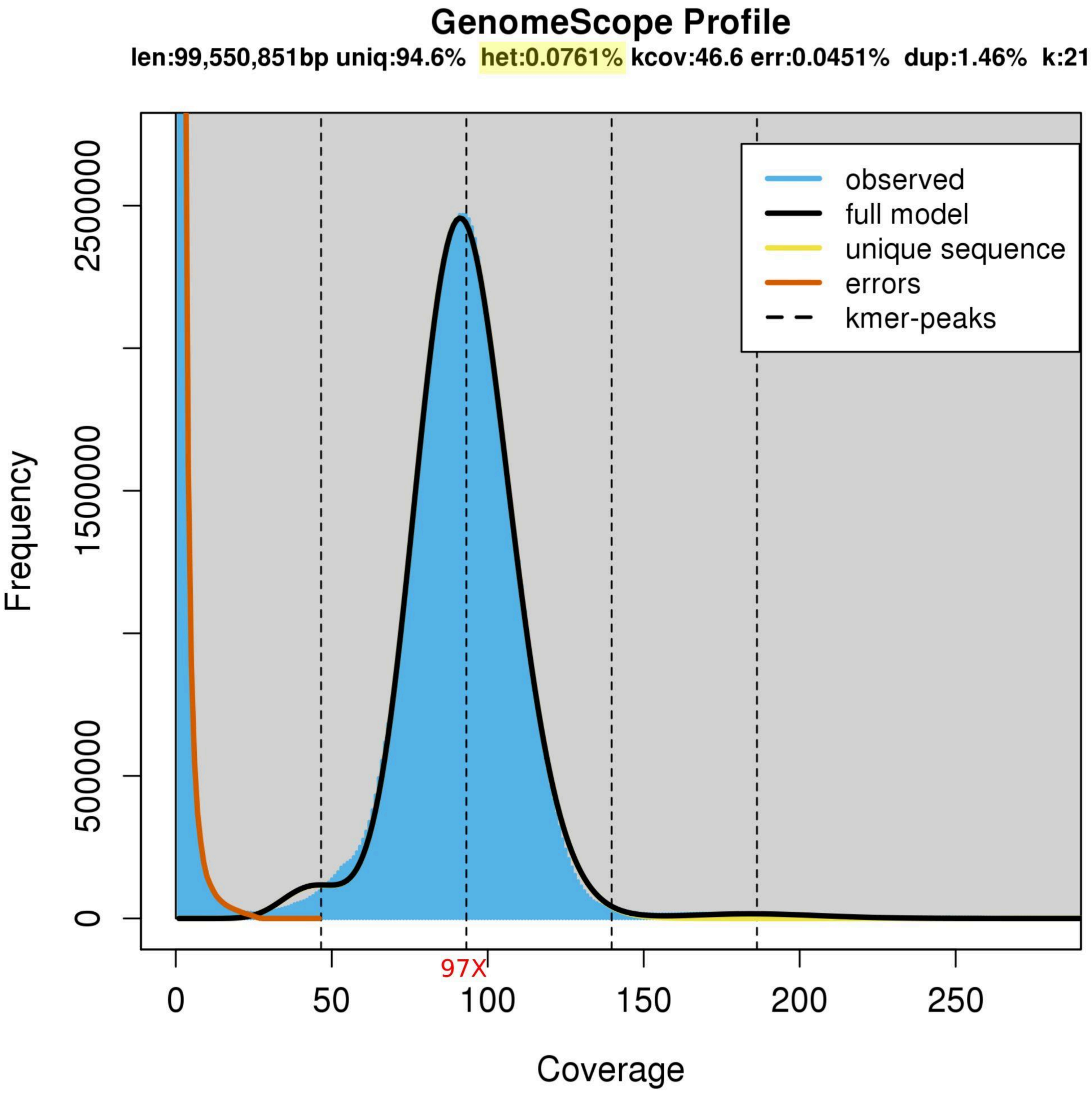

Supplemental Fig 2. Estimation of heterozygosity (yellow highlight) using genomescope - **iso-1**. Additionally, the red 97X marks the homozygous peak which corresponds to the approximate genome wide coverage. (correlate with mapping based estimates from Supp Fig 1A)

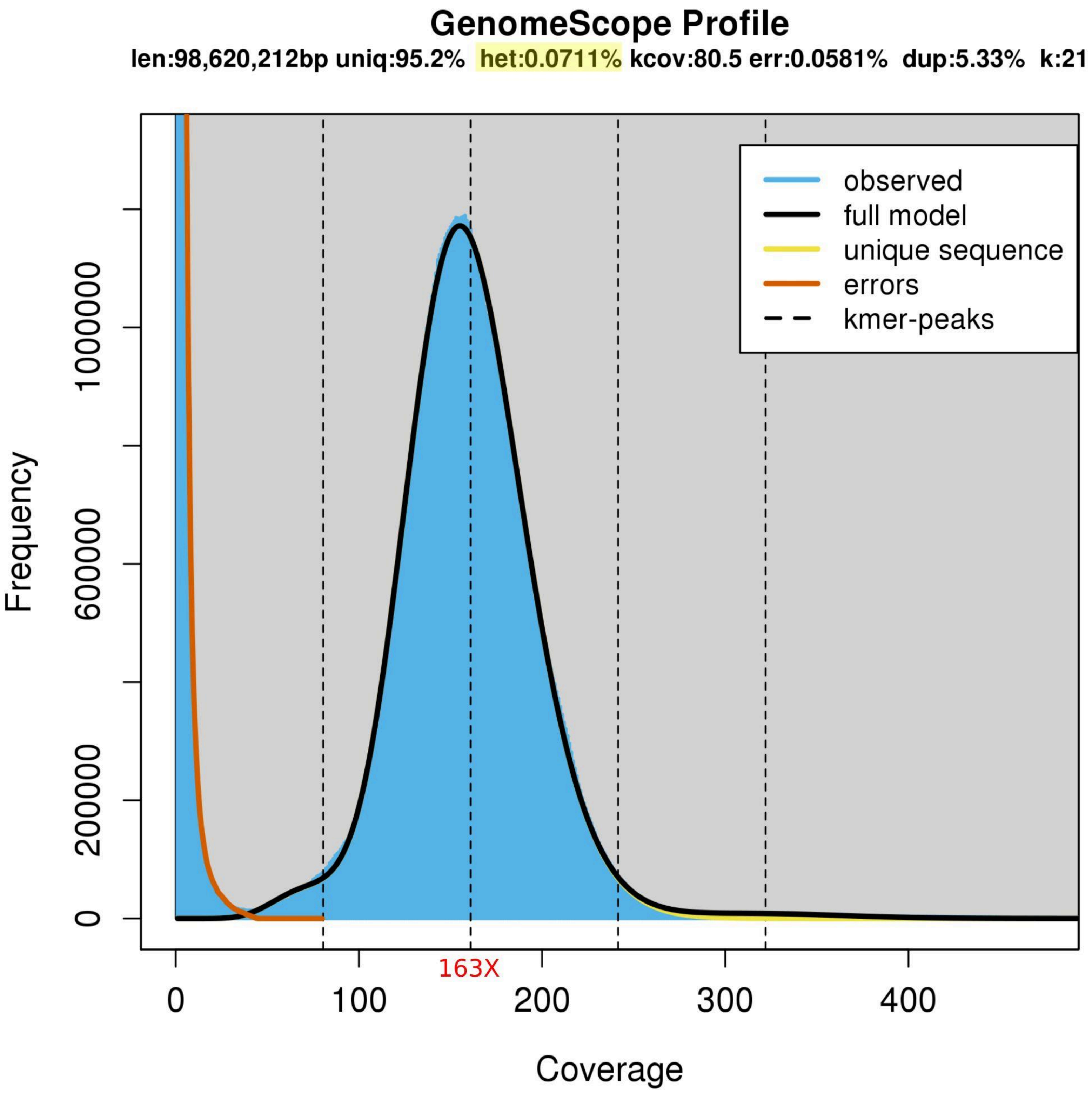

Supplemental Fig 3. Estimation of heterozygosity (yellow highlight) using genomescope - **A4**. Additionally, the red 163X marks the homozygous peak which corresponds to the approximate genome wide coverage.

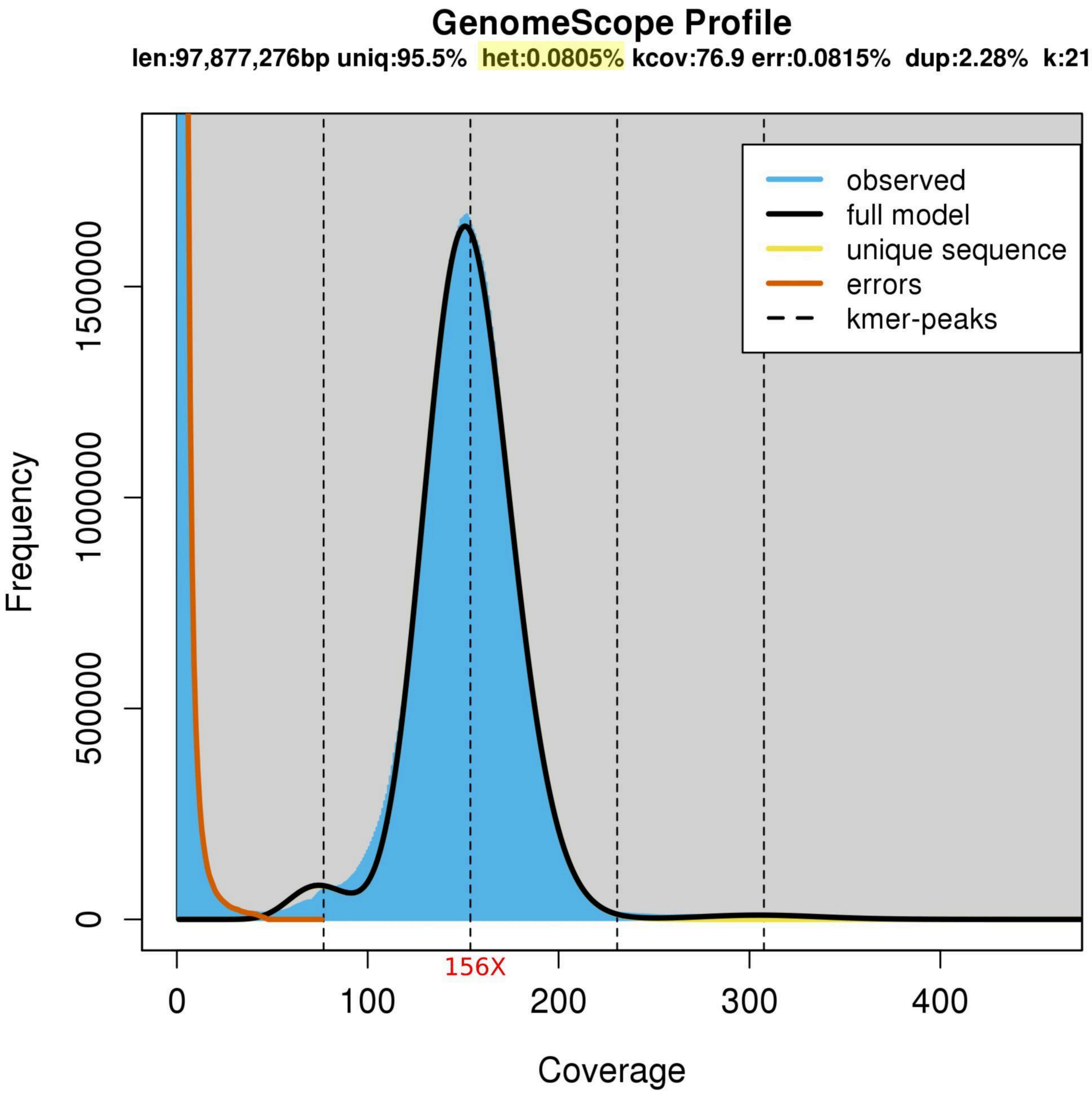

Supplemental Fig 4. Estimation of heterozygosity (yellow highlight) using genomescope - **A3**. Additionally, the red 156✕ marks the homozygous peak which corresponds to the approximate genome wide coverage.

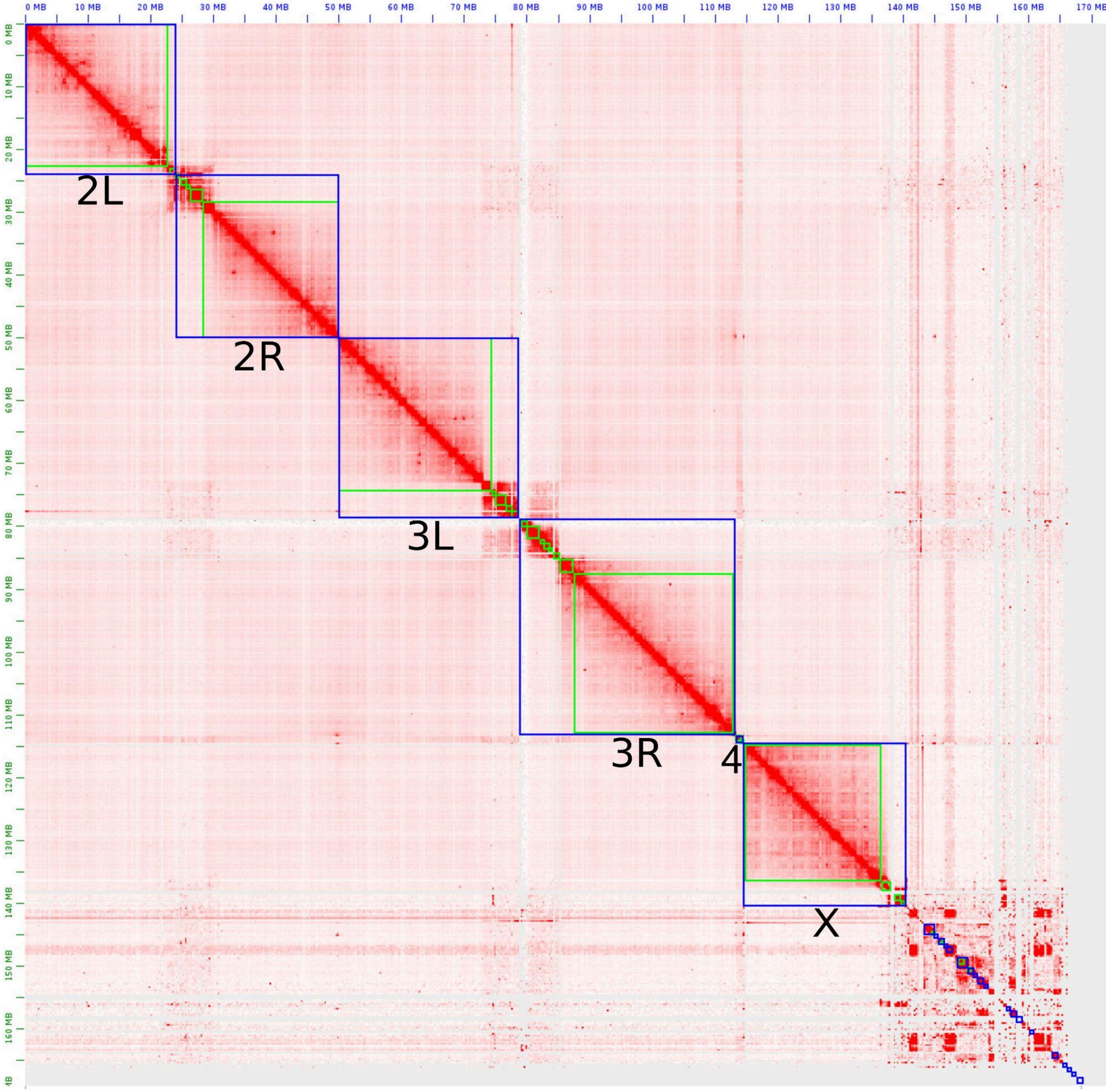

Supplemental Fig 5. HiC Scaffolding of iso-1 hifi contig assembly

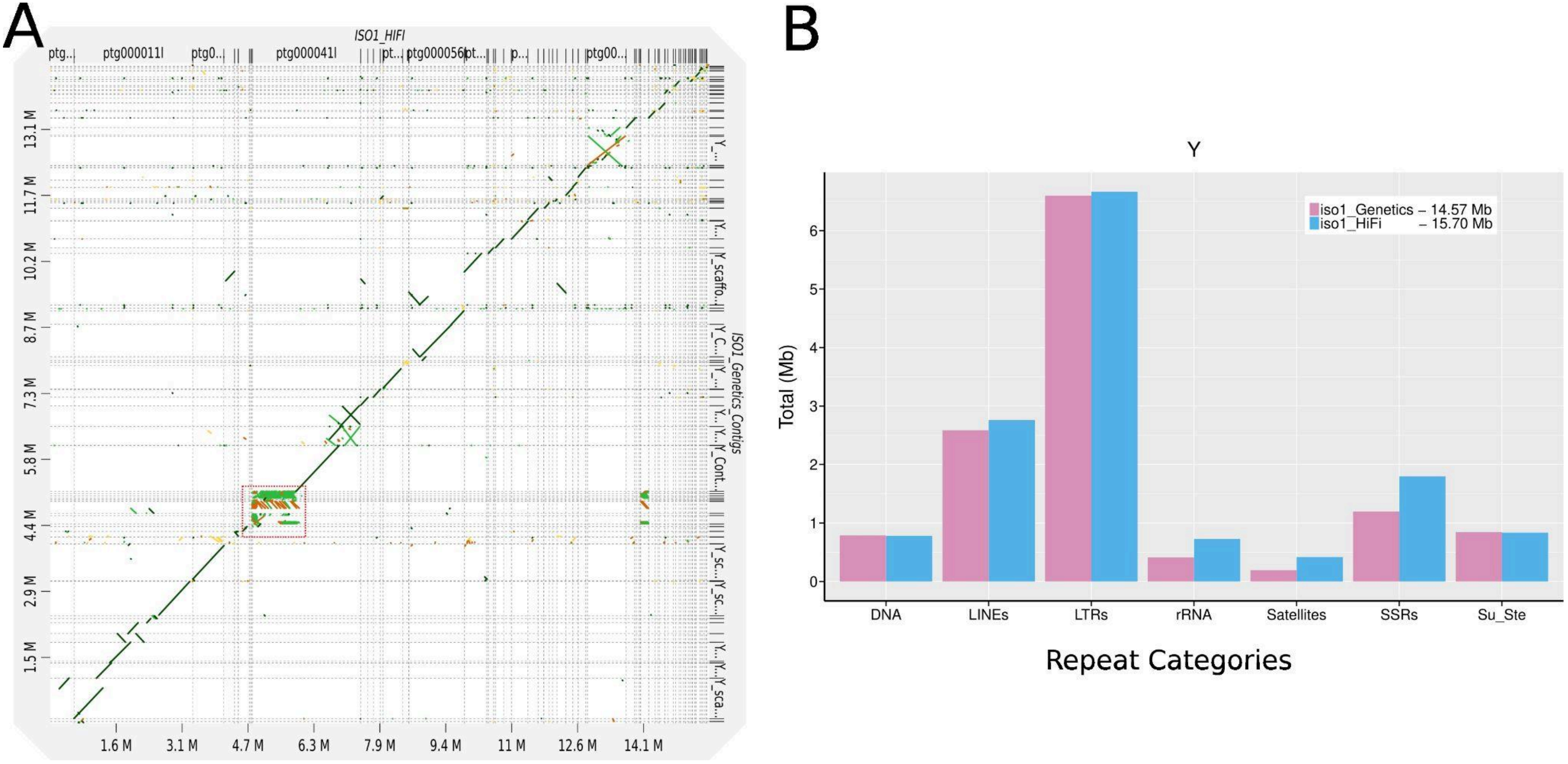

Supplemental Fig 6A. Genomic dot plot showing synteny between iso-1 HiFi Y-contigs (X-axis) vs iso-1 HetEnr Y-contigs (Y-axis).
Supplemental Fig 6B. Repeat Content comparison between the two Y assemblies. They are very similar. iso-1 Hifi assembly just has more rRNA sequences assembled and as a consequence has more rRNA, rRNA associated satellites and rRNA associated R1,R2 LINEs. The beginning of contig 041l (red box) is the rRNA assembled locus. ~80% of the Y Chromosome consists of simple tandem repeats. Our iso-1 HiFi assembly also recovers more tandem repeats (SSRs).

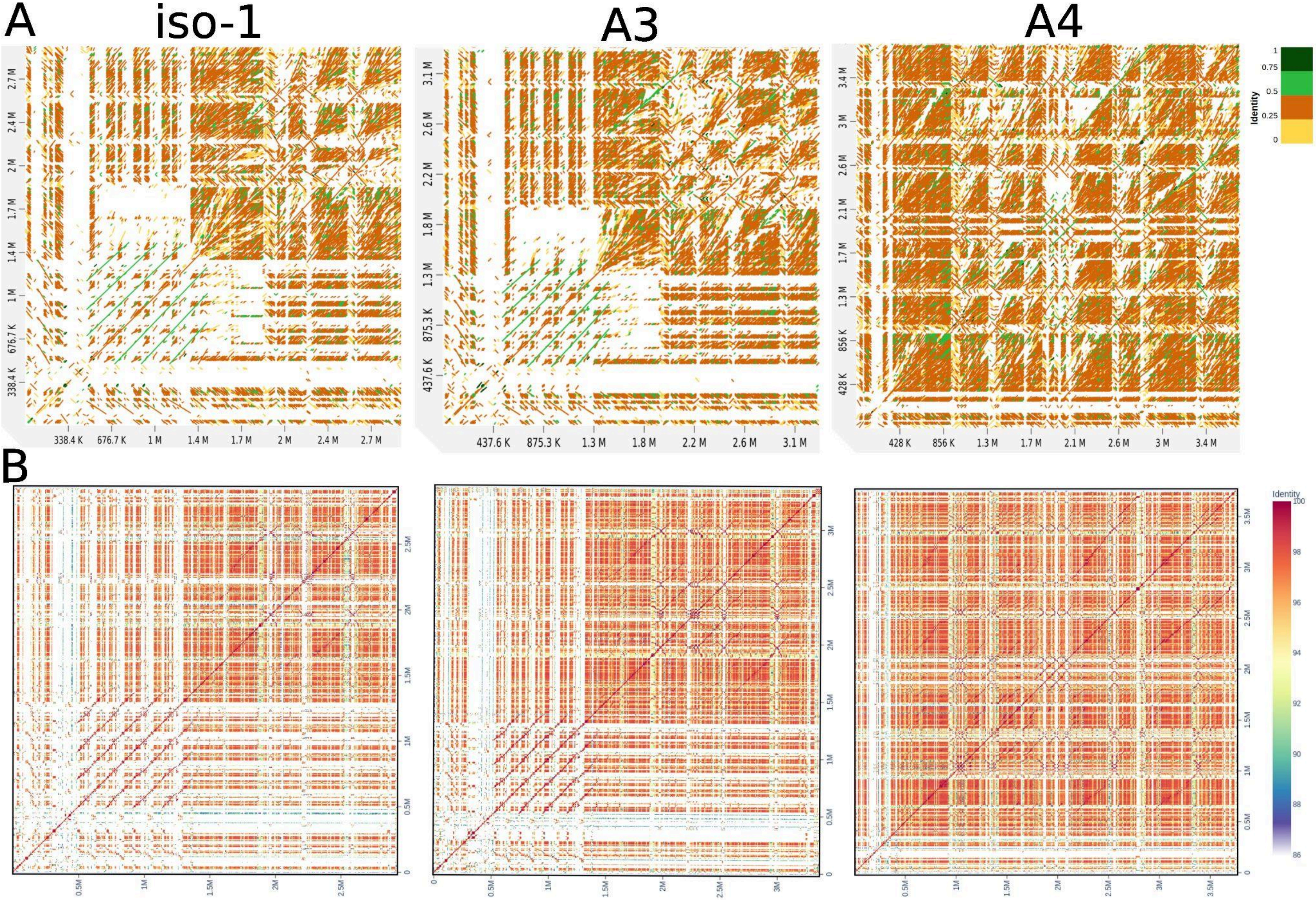

Supplemental Fig 7 A and B. Intra-strain self dot plots of newly assembled X sequences (excluding the rDNA array) in the three assembled strains. The X and Y axis for each plot are identical (the newly assembled X sequence for the corresponding strain). Plots in **A** are from *D-Genies* and plots in **B** are from *ModDotPlot.*

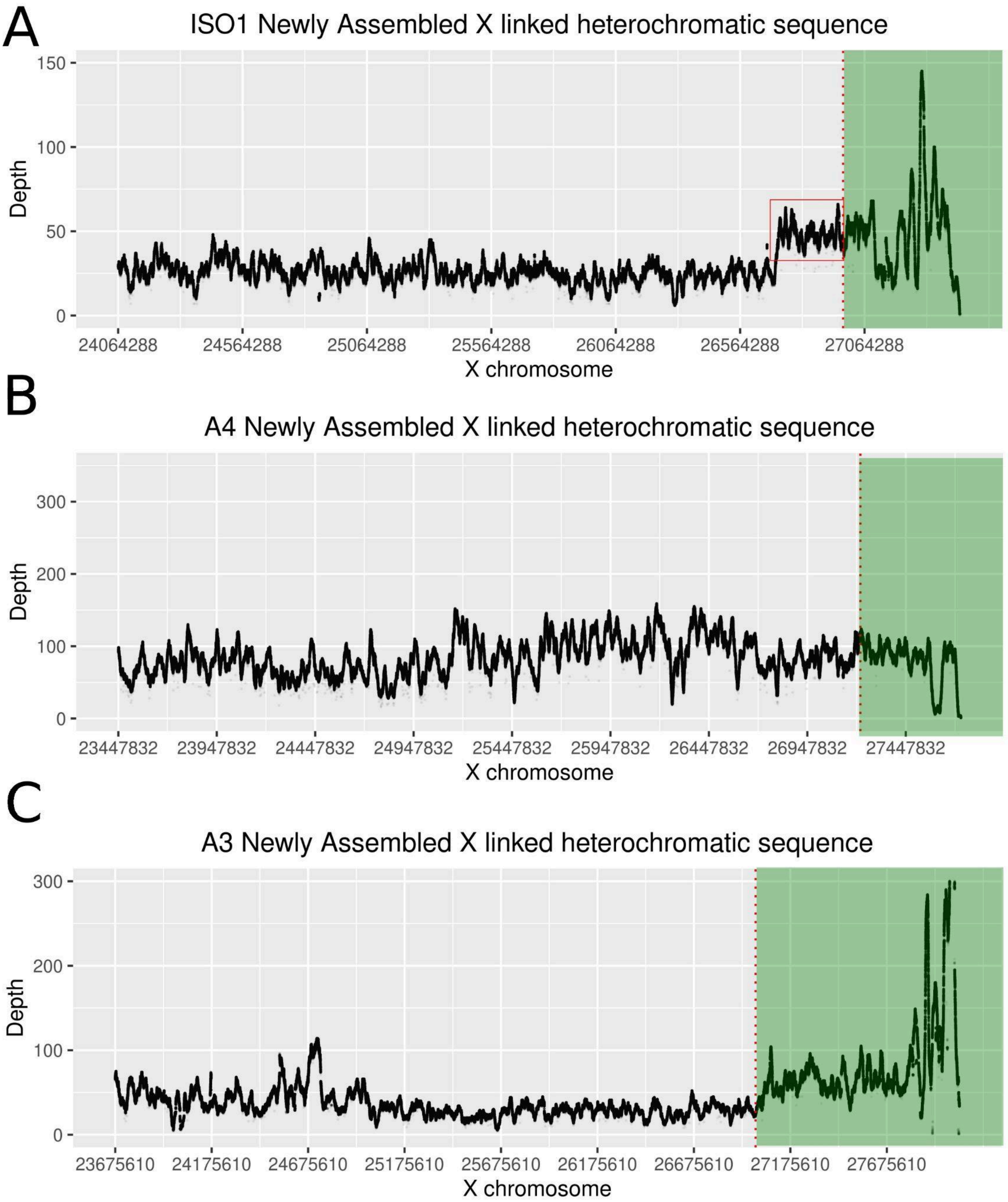

Supplemental Fig 8. The depth plots(using HiFi reads) of newly assembled X linked sequences in the 3 strains. The green box represents the rDNA cluster. The red box in iso-1 hints at a possible ~250 kb collapse in our iso-1 hifi assembly

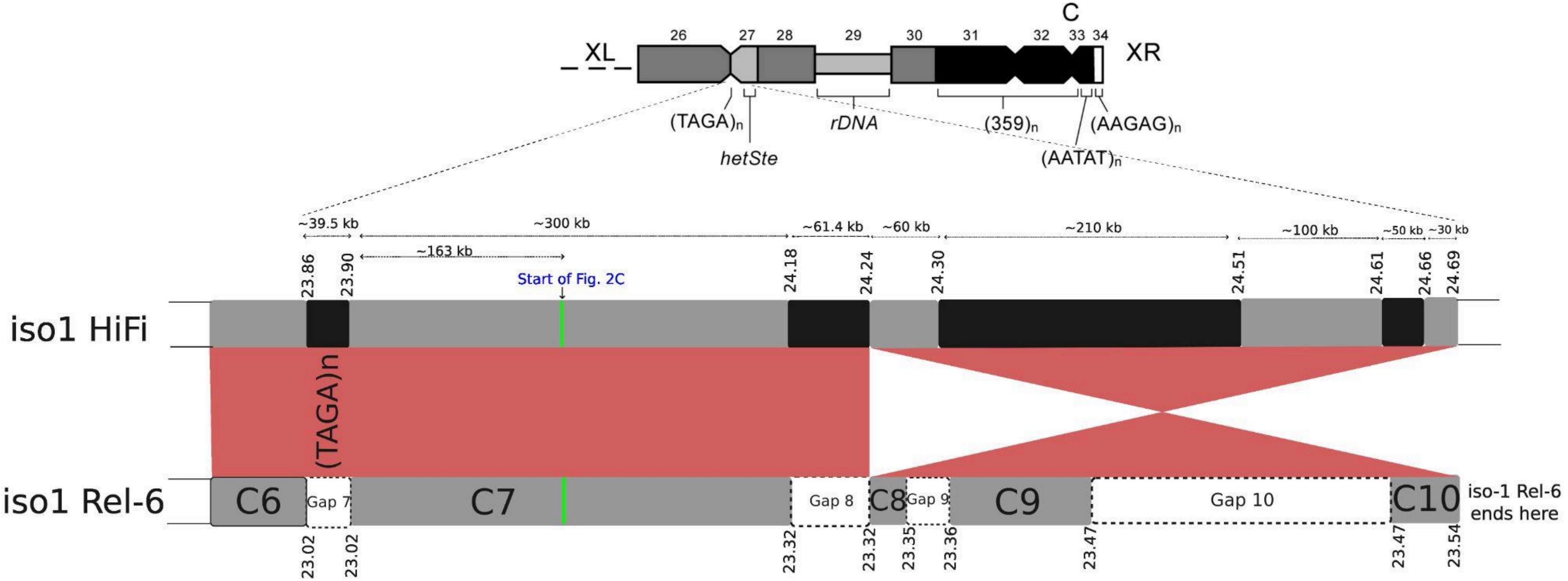

Supplemental Fig 9. This figure describes the X heterochromatin spanning the *h26-h27* junction and initial parts of *h27* and the segments corresponding to that region in the iso-1 HiFi and iso-1 Rel6 assemblies. The grey regions highlight the homologous sequences assembled in both assemblies whereas black regions highlight filled gaps. *(TAGA)n* array sits between the *h26* and *h27* bands and is present in release 6, as well as our HiFi assembly of iso-1. ~300kb of the distal flank of *h27* is present in Rel6 followed by 3 small contigs, all of which are inverted relative to our assembly. The Rel6 paper states that the proximal 2 scaffolds in X PCH lack orientation evidence (Results-The X Chromosome-last paragraph), explaining why sequences in Rel6 (i.e., contigs C8, C9, and C10) are inverted relative to our iso-1 HiFi towards the proximal end. This also explains the negative orientation of segments highlighted in main Fig 2A. The green segment labeled “Start of Fig. 2C” represents where our main Fig. 2C starts. This was our reasoning behind the choice: this position was the first occurrence of any R1 TE sequence, including fragments, in the region.

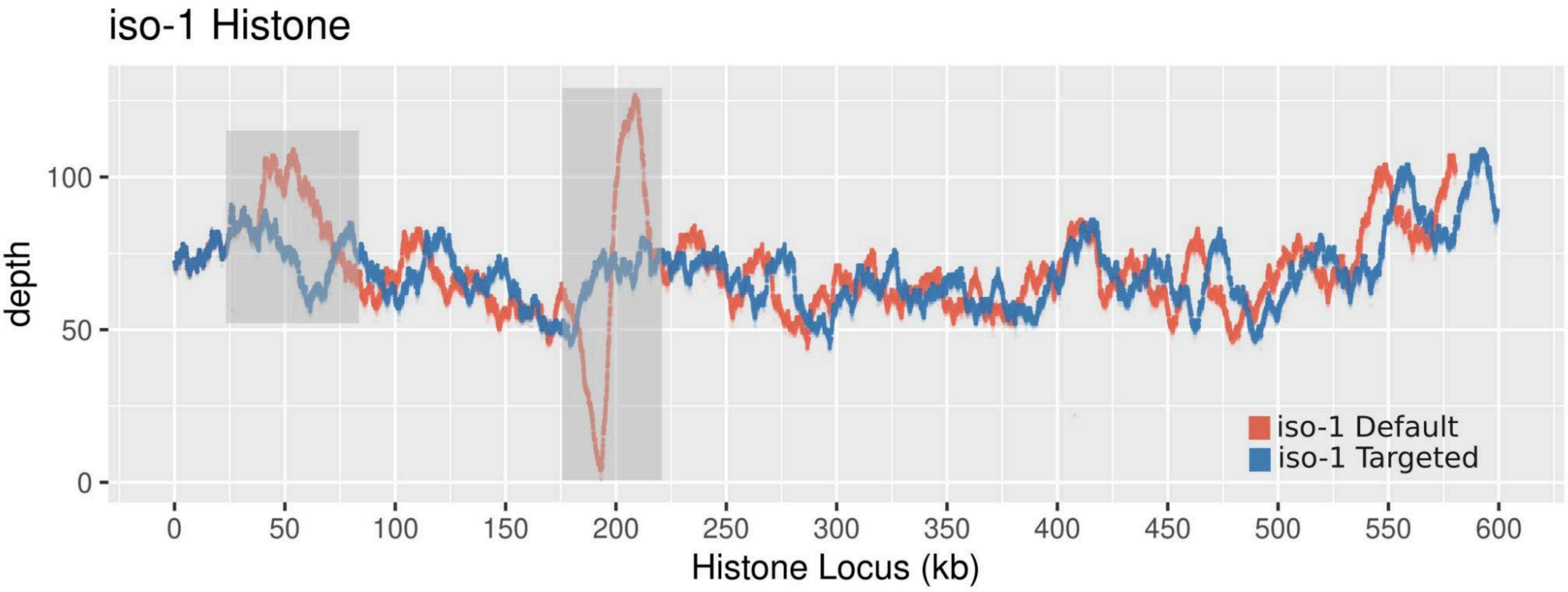

Supplemental Fig 10. The depth plots of HiFi reads mapped to the Default and Targeted histone locus for iso-1. The gray boxes highlight a possible collapse in the beginning of the cluster (~50kb) and a misassembly (~200kb).

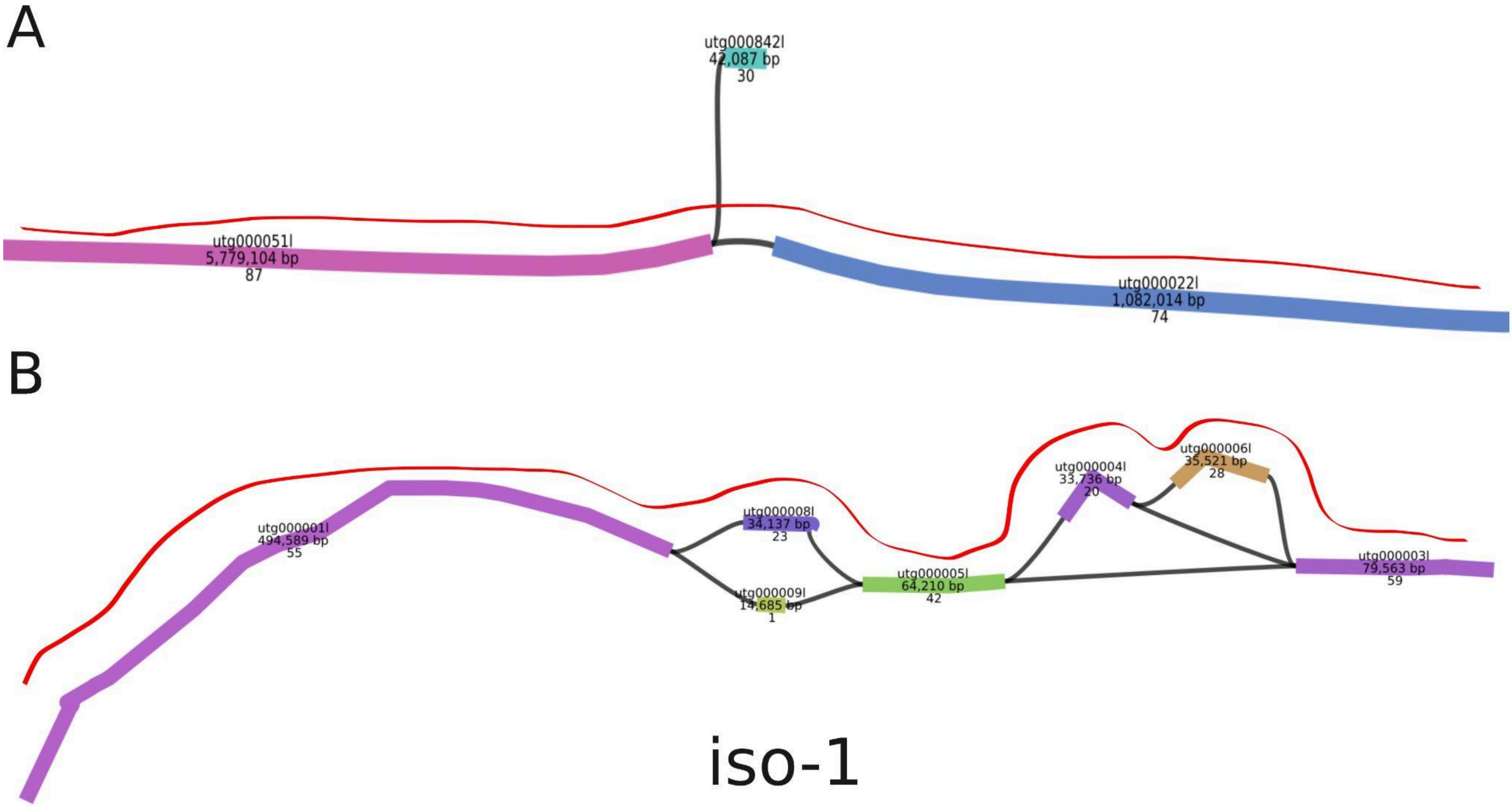

Supplemental Fig 11A. The 3 *unitigs* that contain the histone locus in the original iso-1 assembly. The 1st line of the node label is nodename. The 2nd line is the length of the node. The 3rd line is the depth (scales to number of reads covering that particular node). The red line indicates the possible path taken by the assembler to get through the locus.

Supplemental Fig 11B. The unitig graph of the targeted histone assembly for iso-1.The red line indicates the possible path taken by assembler to get through the locus.

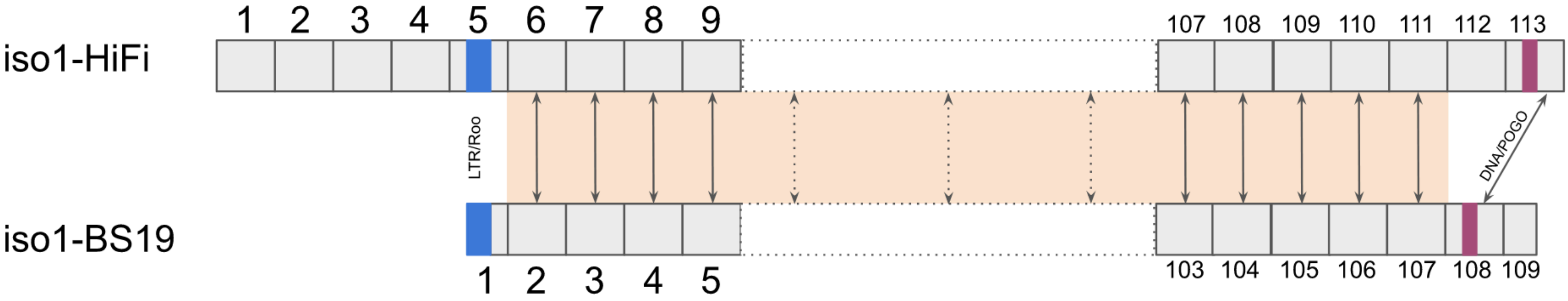

Supplemental Fig 12. A schematic representation of comparison between iso1-HiFi histone cluster vs iso-1 BS19 histone cluster. On the distal end, the iso-1-BS19 omits the first 4 units in our assembly, starting with a partially assembled unit containing a *roo* retrotransposon corresponding to the 5th copy in our assembly. For simplicity of comparison, we start with the first complete unit. On the proximal flank, there seems to be a minor difference in the structure. This might be due to assembly error in iso-1-BS19 or sub-strain differences. Bongartz et al. also briefly touch upon this in the last paragraph before the *Discussion* section of their paper. They find some segregating variation in the dataset they assembled. Even datasets derived from isogenic stocks/strains kept in a single lab can segregate for variation in such regions let alone strains spread across various academic labs and stock centers.

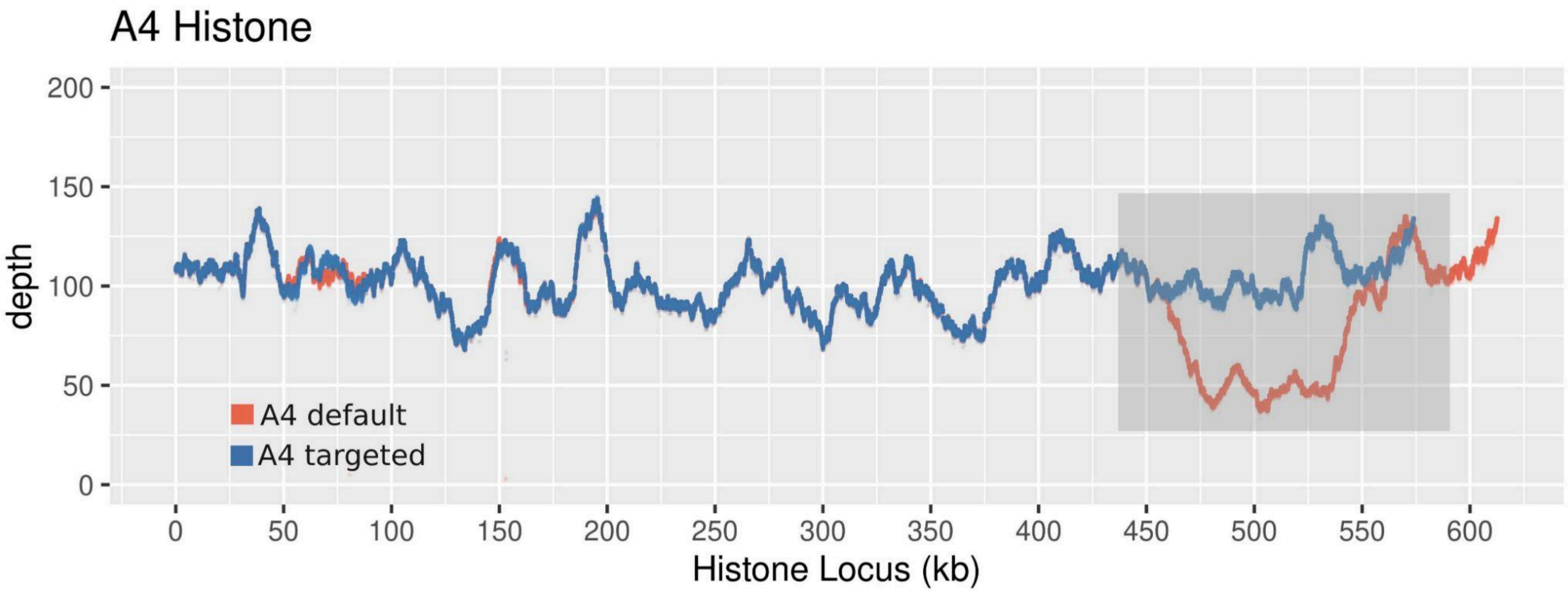

Supplemental Fig 13. The depth plots for A4 default and A4 targeted histone locus. There is a drop in coverage (~half) starting at ~425 kb to ~550 kb (gray box) in the default assembly. This tells us that a region present once in the genome is represented twice in the assembly. The depth plot for targeted assembly is relatively uniform, indicating no obvious/glaring mis-assemblies.

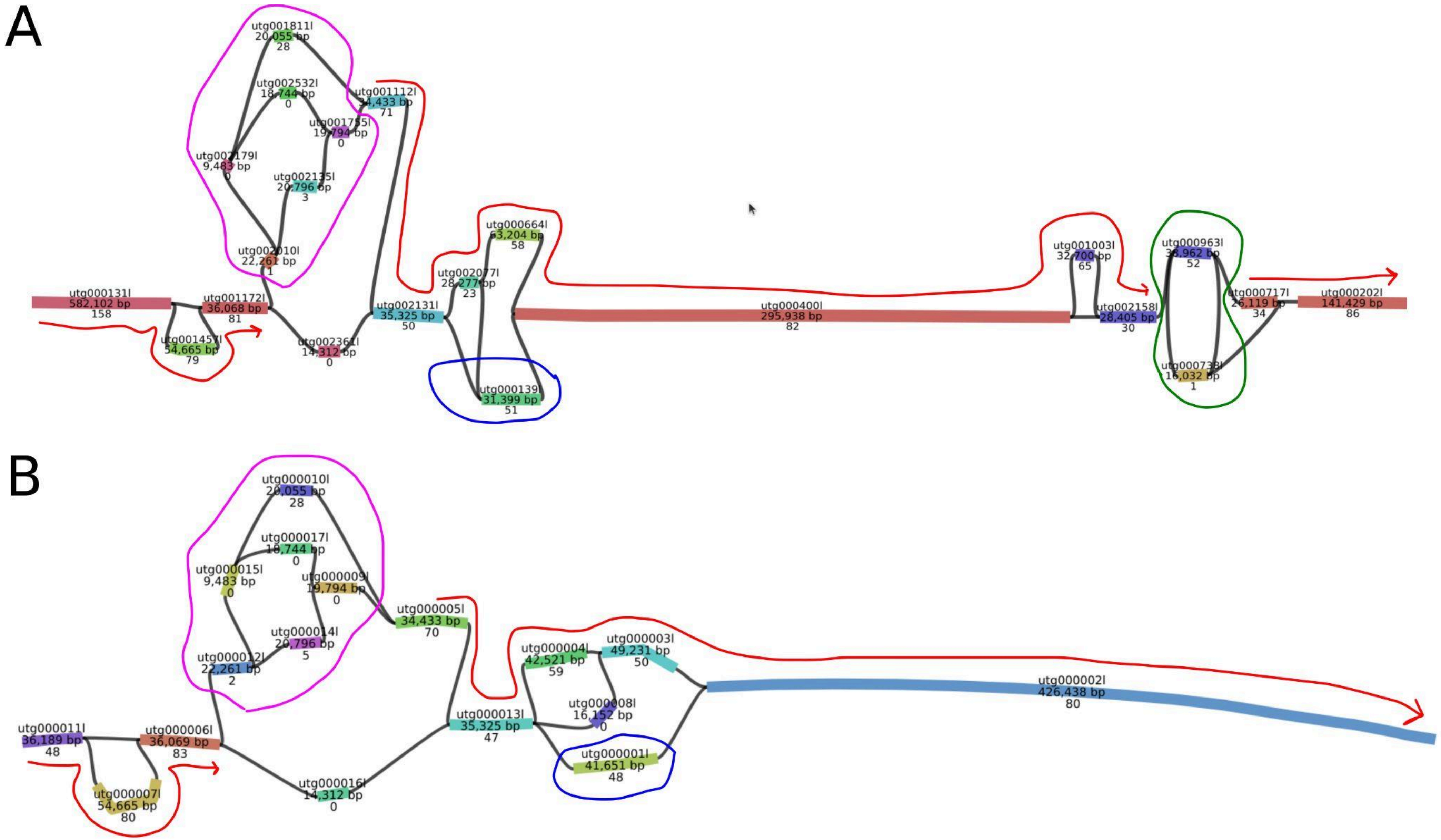

Supplemental Fig 14 A & B. The unitig graph for default (A) and targeted histone (B) assembly in A4. The 1st line of the node label is nodename. The 2nd line is the length of the node. The 3rd line is the depth. The green ~circle in A highlights the possible reason for drop in coverage seen in the depth plot (by visiting the nodes twice). The pink ~circle present in both graphs might represent a low coverage or complex region or segregating alleles . The red line indicates the possible path taken by the assembler to get through the locus. In both graphs, the high depth node highlighted by the blue circle doesn't get included in the primary contig. This might represent a high frequency segregating allele in the sequenced pool (since we sequenced ~200 diploid males).

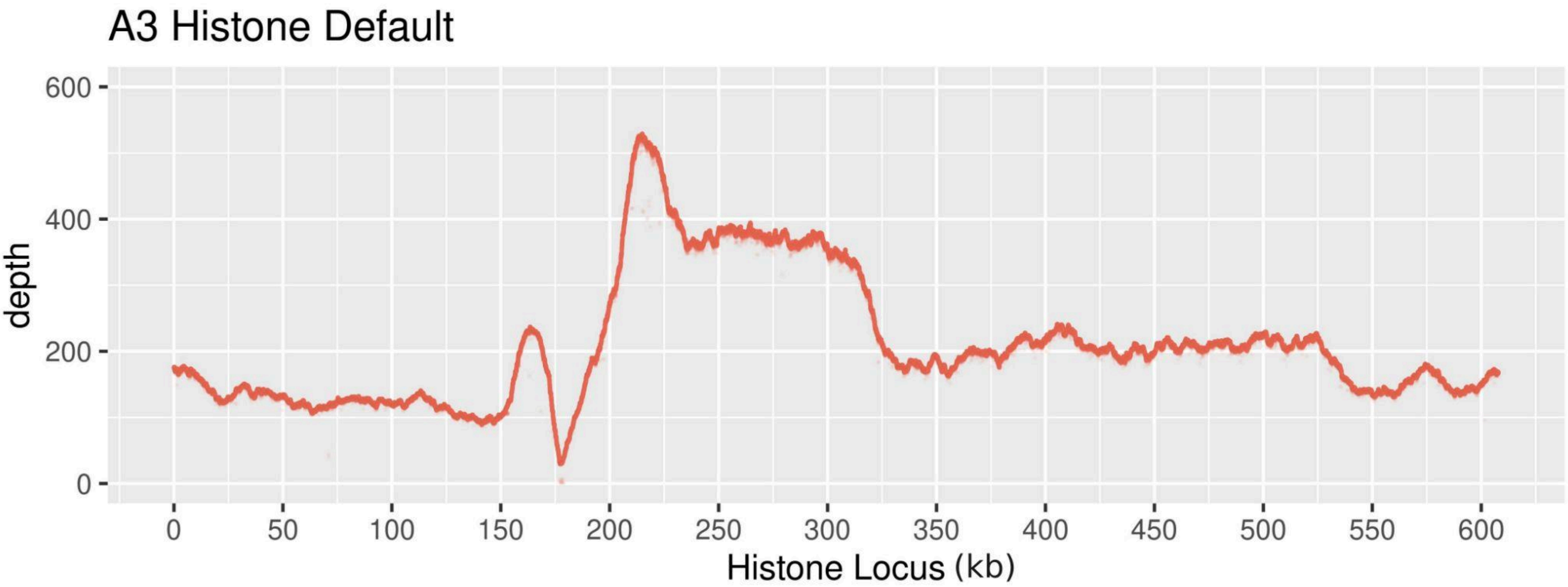

Supplemental Fig 15. The depth plot for A3 default histone locus. There are multiple issues with the assembly starting at ~150 kb.

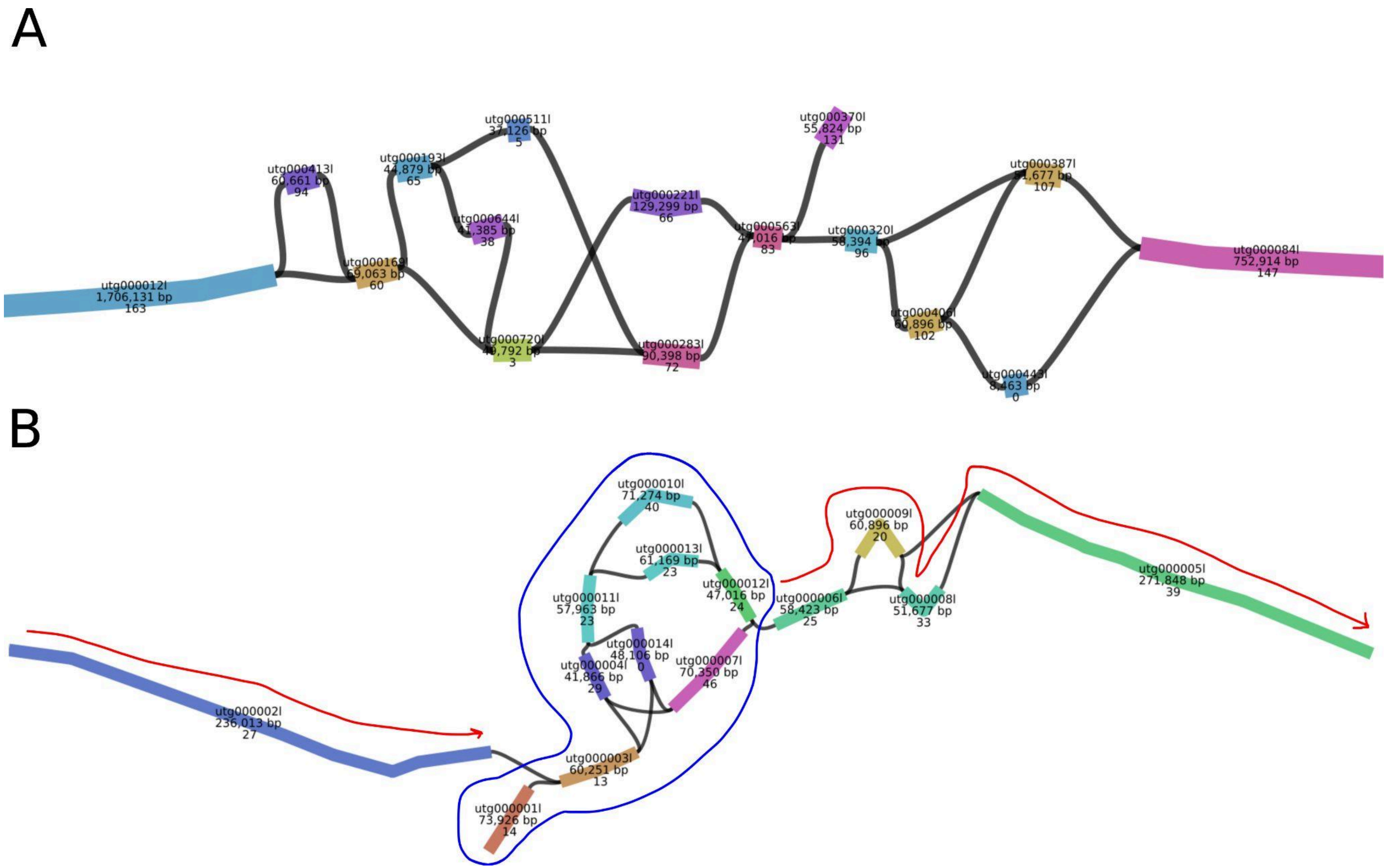

Supplemental Fig 16 A & B. The unitig graph for default (A) and longest-40X targeted (B) assemblies. The red line in B represents the well resolved parts of the graph. The blue circle represents the complex region in the middle (path through it cannot be cannot be unambiguously determined).

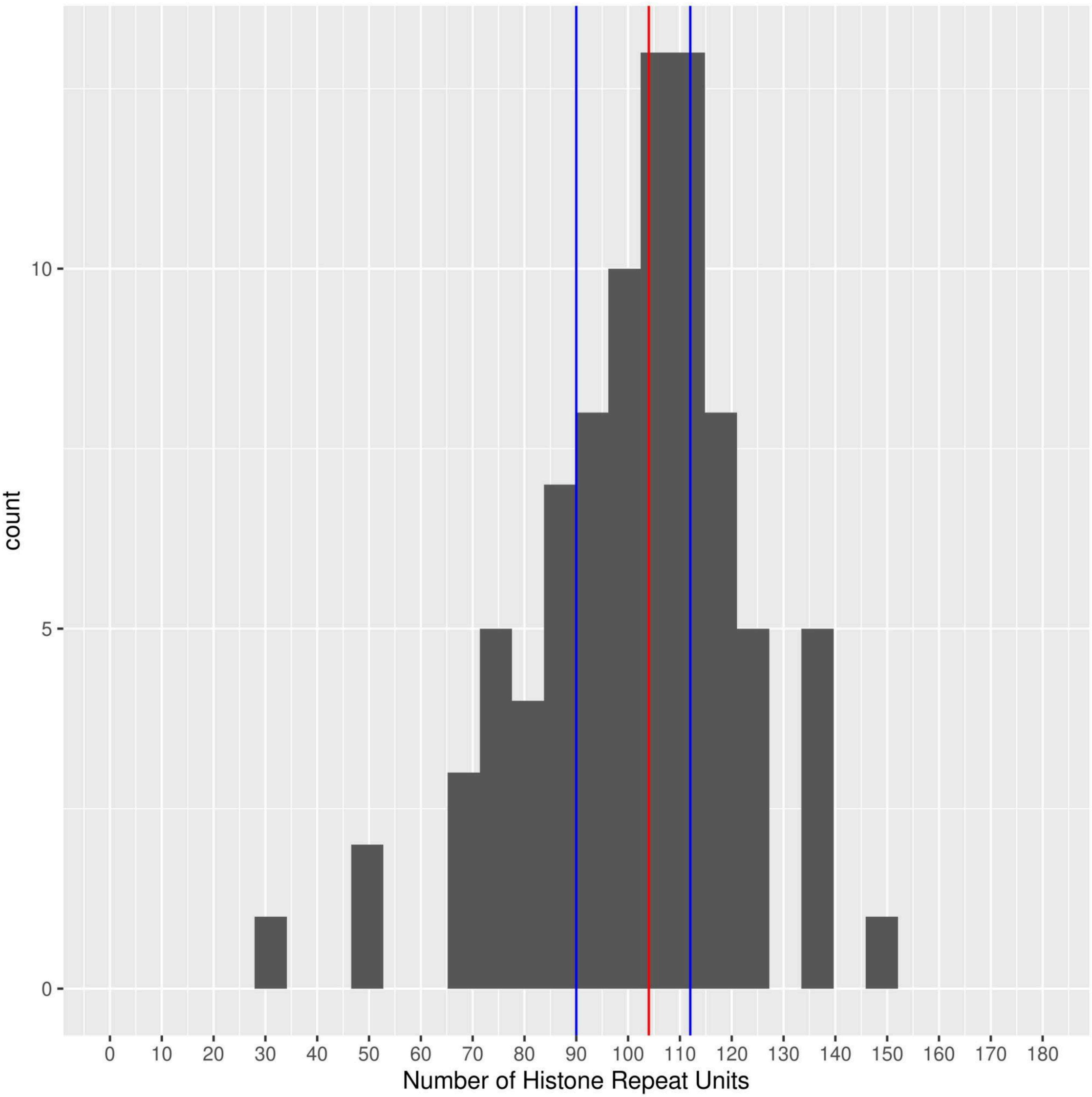

Supplemental Fig 17. Histone copy number distribution of the GDL strains. The red line indicates the median and the blue lines represent the first (Q1) and the third (Q3) quartile.

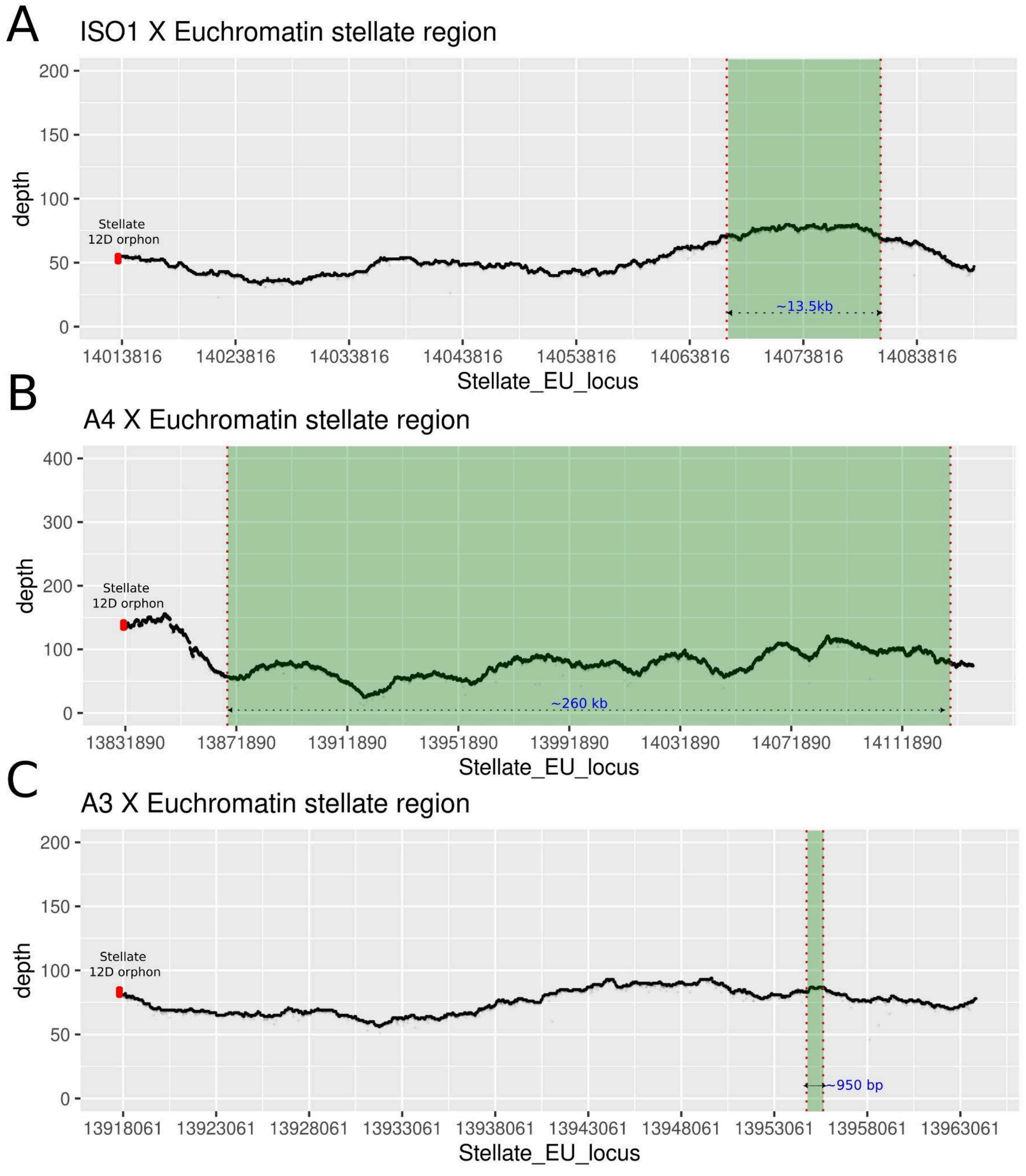

Supplemental Fig 18 A B C. HiFi depth plots for iso-1 (A), A4 (B) and A3 (C) for the euchromatin stellate locus. The red mark indicates the start of *Stellate 12D orphan copy*. The green rectangle highlights the stellate proper tandem cluster.

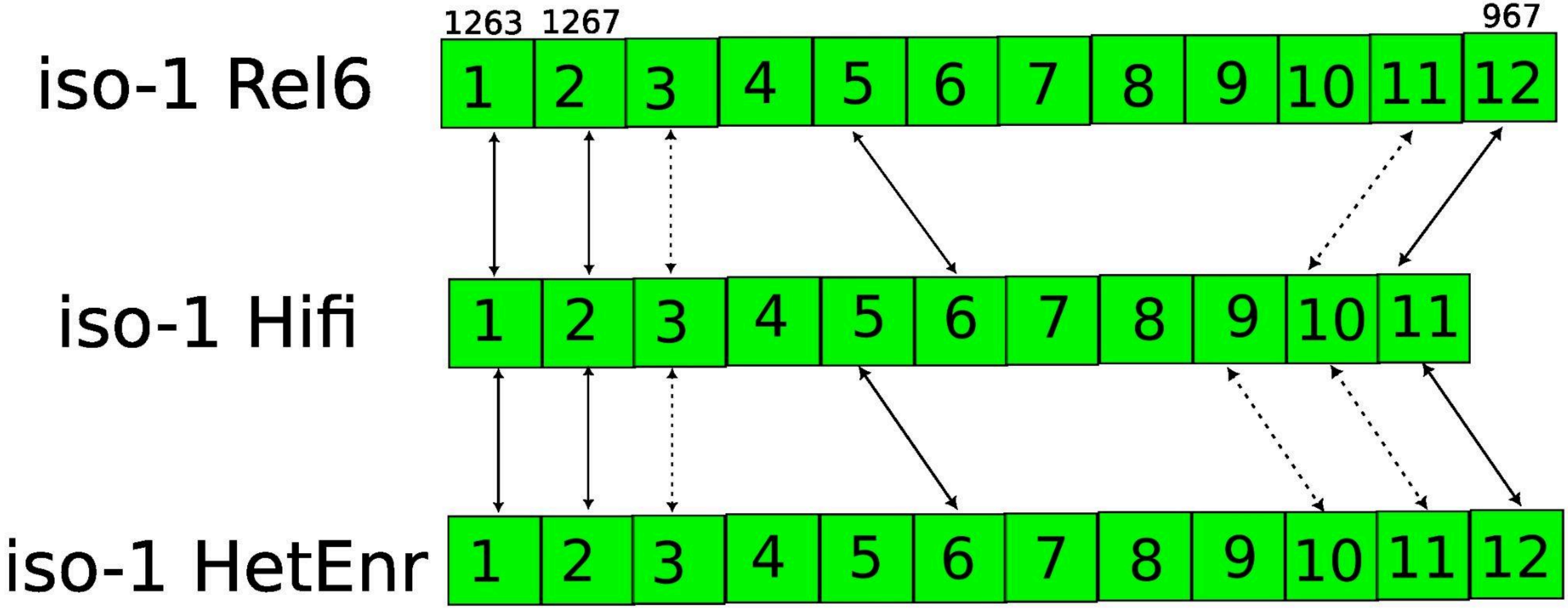

Supplemental Fig 19. A schematic representation of euchromatic stellate clusters in the 3 iso-1 assemblies and their comparison. The solid arrows represent the anchors identified using phylogenetic methods. The dashed arrow indicates possible/probable anchors marked manually using proximity (synteny) information to the phylogenetically identified anchors (solid arrows). The numbers on the top represent the lengths of the stellate units (which deviate from the canonical 1269 bp unit).

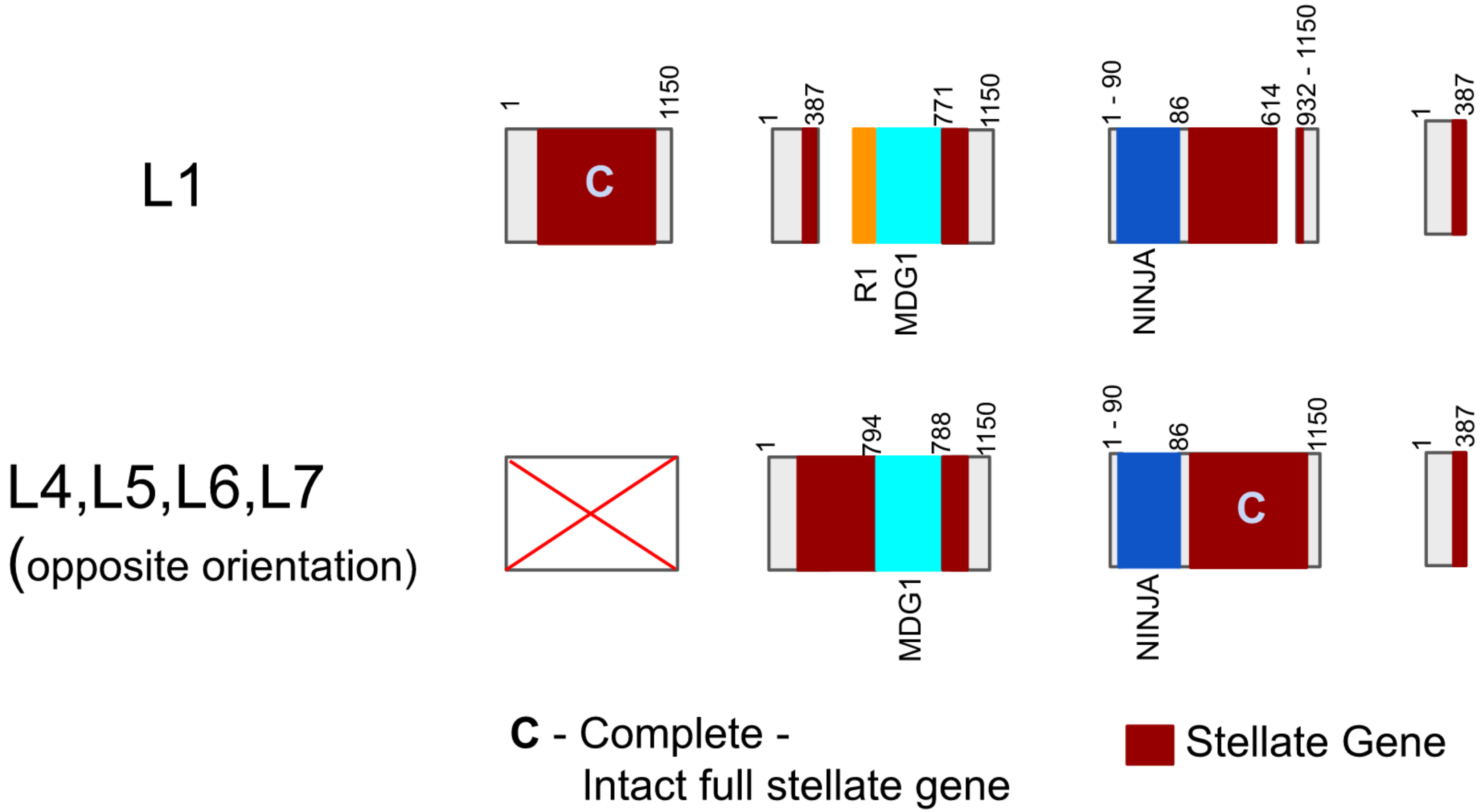

Supplemental Fig 20. A schematic representation of Type_1 locus in iso-1. The numbers on the top are coordinates corresponding to the full length (1150) repeat unit for each copy. R1, NDG1 and NINJA represent TE insertions. The red box marks the *stellate gene* embedded within the whole 1150 repeat unit. **C** indicates a full length *Stellate* repeat unit/gene.

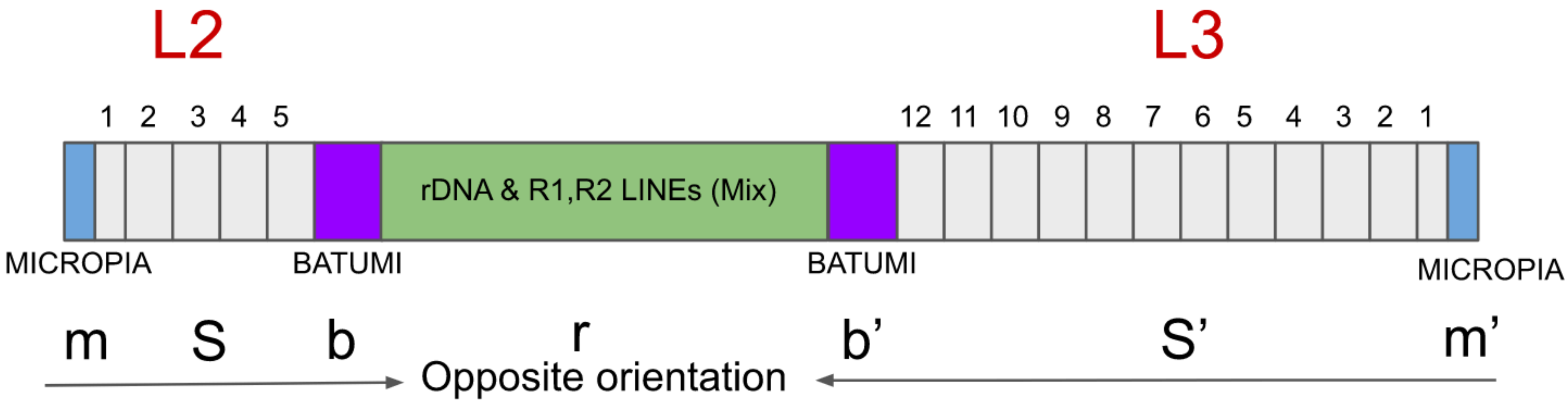

Supplemental Fig 21. A schematic representation of Type_2 loci (L2 and L3) and their placement with respect to each other. The numbers on the top correspond to a particular copy in the tandem array. MICROPIA and BATUMI are LTR TEs. m,s,b,r are shorthand used to describe m-MICROPIA, S-Stellate array, b-BATUMI and r-rDNA & R1,R2 LINEs (Mix). ‘ indicates opposite orientation. The Unit labeled as 1 has a partial deletion (highlighted by short box length).

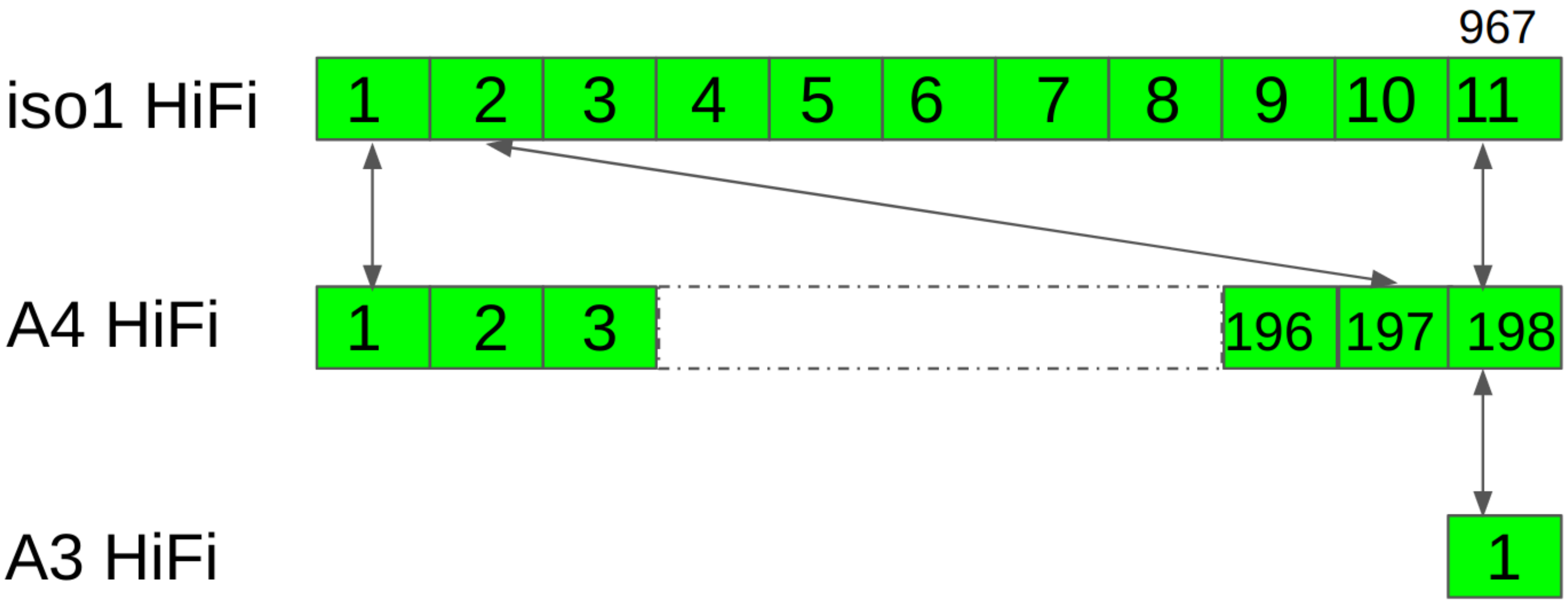

Supplemental Fig 22. A schematic representation of euchromatic stellate clusters in iso-1,A4 and A3 assemblies and their comparison. The numbers in the box designate the unit in an array. The dotted gray box in A4 represents unit 4-195. The solid arrows indicate anchors identified using phylogenetic approaches. The number (967) on top of last unit represents the most proximal unit (with 302 bp deletion)

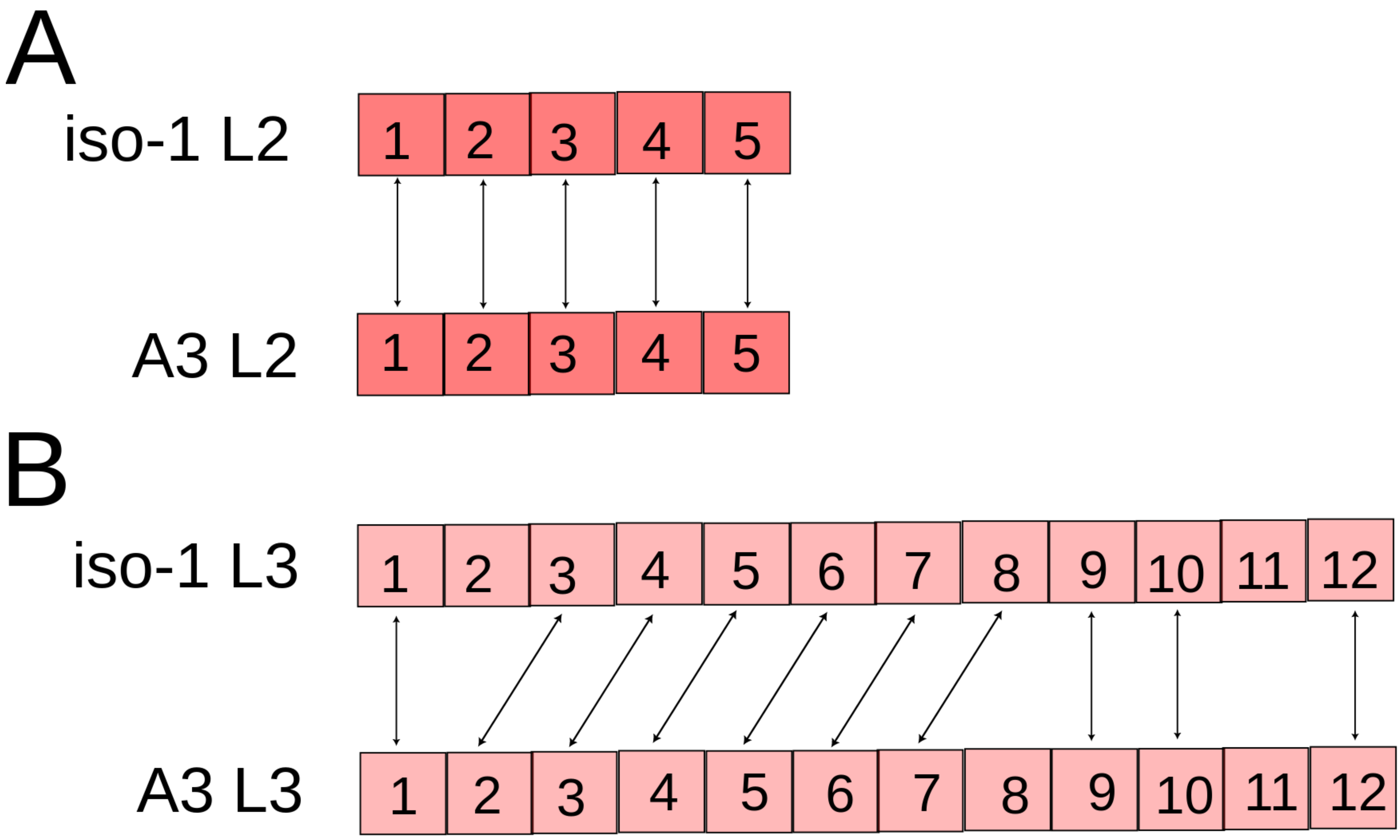

Supplemental Fig 23. A schematic representation of heterochromatic stellate clusters in iso-1 and A3 assemblies and their comparison. There is one to one correspondence for all units in L2 **(A)**. iso-1 L3 and A3 L3 are mostly similar with some minor differences **(B)**.

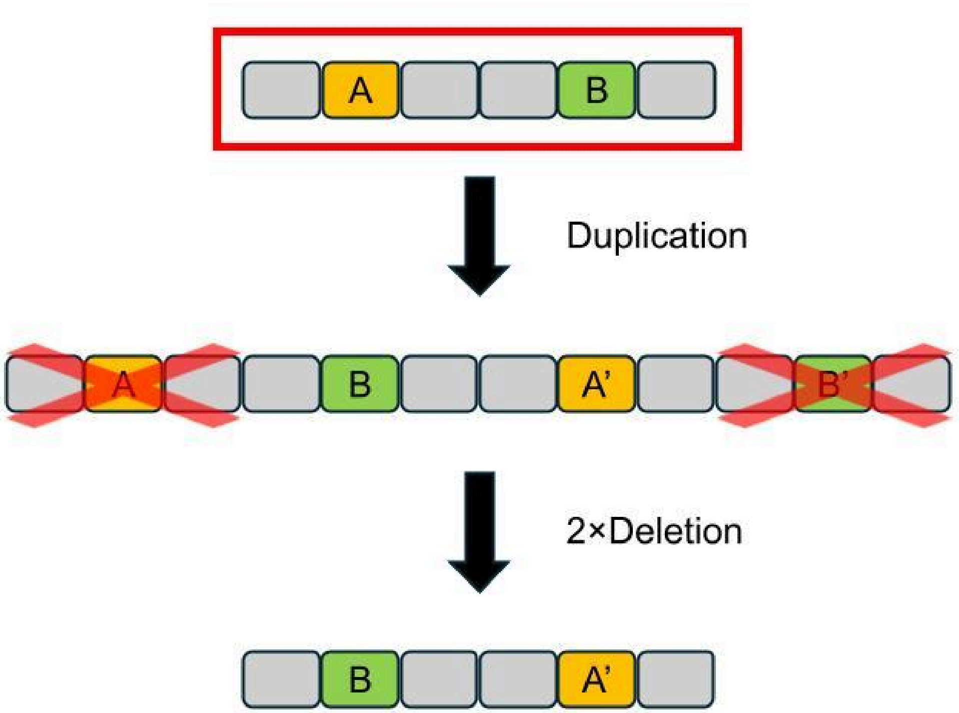

Supplemental Fig 24. Two possible scenarios demonstrating mutational steps that can result in anchor points swapping their relative order in the array.

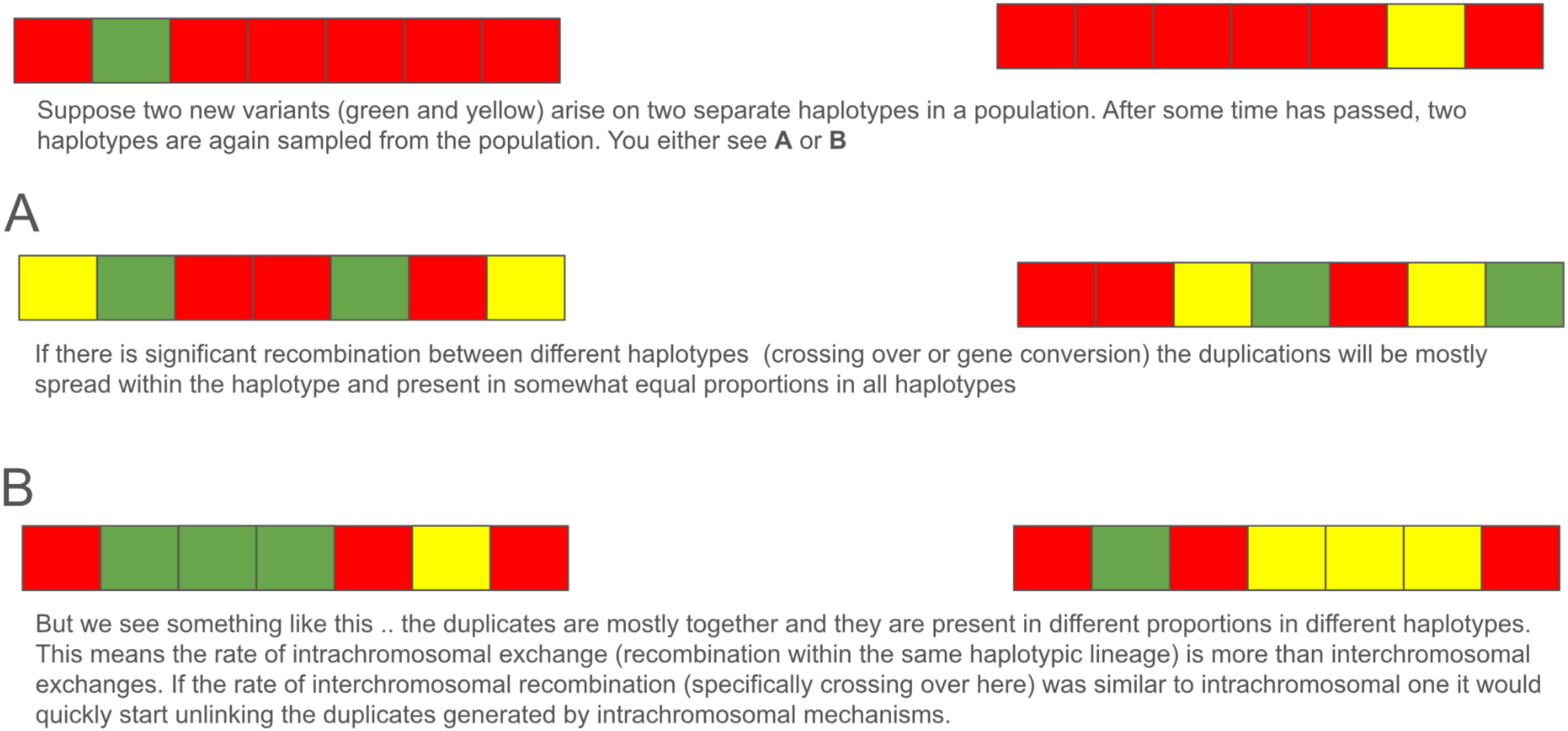

Supplemental Fig 25. Simplified diagrams demonstrating two possible outcomes of spread of newly arisen variants in a population, given some rate of inter- and intra-chromosomal exchanges. Our observations (B) suggest that the rate of intrachromosomal recombination is significantly higher than interchromosomal one.

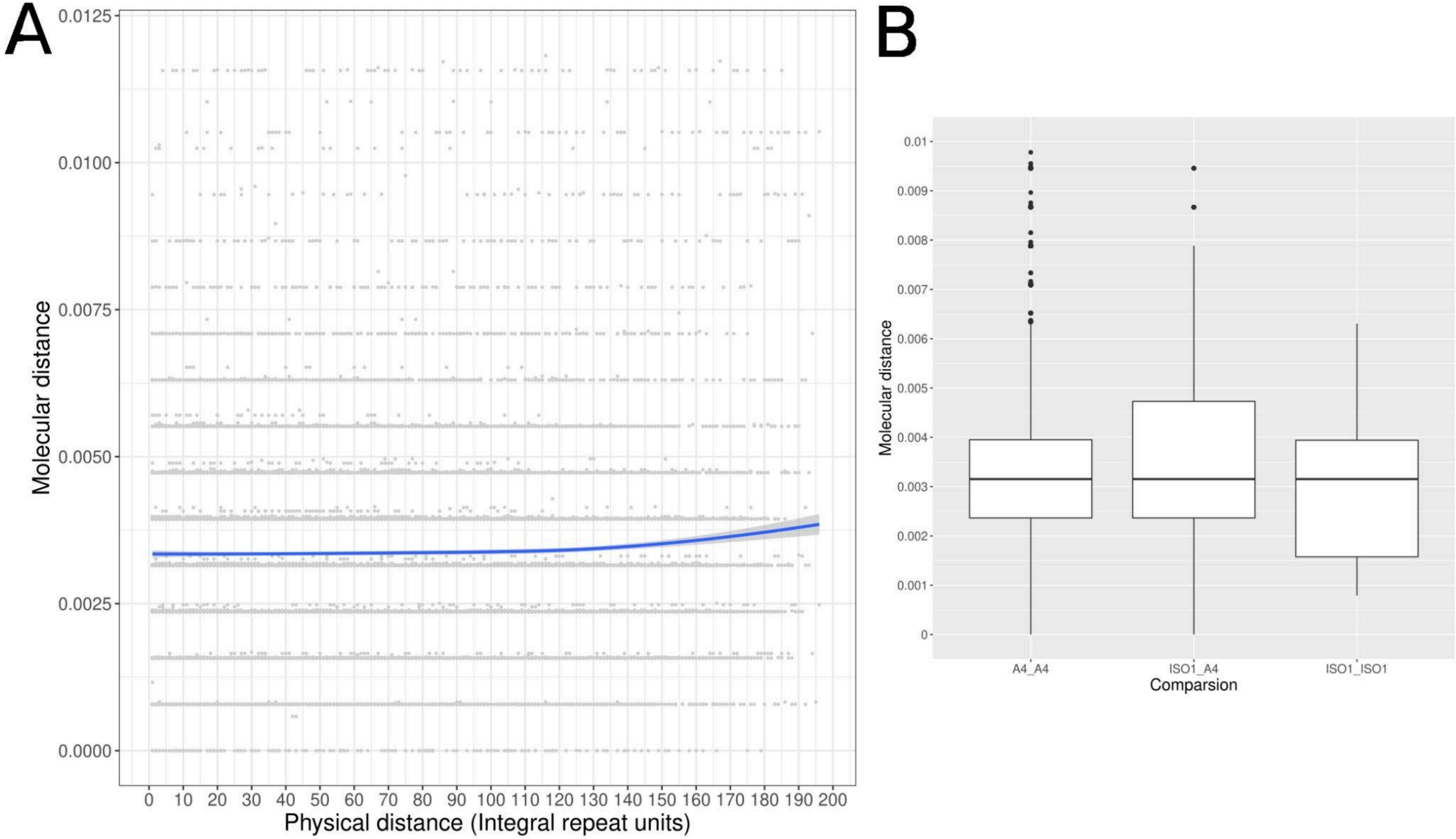

Supplemental Fig 26. (A) Physical vs molecular distance plot for A4 euchromatic stellate array. (Similar to Fig 4C) **(B)** Box plot comparing molecular distance between all pairwise units for within versus between array comparison for iso-1 and A4.
