# Supplementary material for: Genetic variation in recalcitrant repetitive regions of the Drosophila melanogaster genome
Source: Genome Res. 2025 Sep;35(9):2023–40. doi: 10.1101/gr.280728.125 (PMC12400953; doi:10.1101/gr.280728.125)
Supplement: Supplement 2 [file Supplemental_Tables.pdf]

Supplemental Tables For:

Genetic variation in recalcitrant repetitive regions of the *Drosophila melanogaster* genome

Harsh G. Shukla, Mahul Chakraborty, J.J. Emerson

Strain	Throughput (Gb)	Median	Q1	Q2	Approx. Depth
iso-1	12.40	15,026	13,250	17,363	97×
A4	21.28	13,724	12,013	15,779	163×
A3	20.75	15,679	12,920	19,663	156×

Supplemental Table 1. The statistics of HiFi datasets generated in this study

Strain	Assembly size (Mb)	Num of contigs	N50 (Mb)	N90 (Mb)	L50	L90	Largest (Mb)
iso-1	168.56	236	22.96	0.463	4	33	27.23
A4	170.31	277	21.53	0.336	4	38	28.16
A3	172.45	142	21.50	0.831	4	20	28.05

Supplemental Table 2. The statistics of contig level assemblies (after decontamination and removal of redundant mitochondrial contigs)

Strain	Assembly size (Mb)	No. of Scaffolds	N50 (Mb)	N90 (Mb)	L50	L90	Largest (Mb)	No. of Ns	No. of Gaps
iso-1	168.58	212	27.44	0.697	3	13	34.07	24,000	24
A4	170.33	259	27.72	0.368	3	21	34.41	18,000	18
A3	172.46	126	28.05	1.33	3	11	34.39	16,000	16

Supplemental Table 3. The statistics of Scaffolded assemblies

CHR	iso-1 Rel6	iso-1 HiFi	A4 HiFi	A3 HiFi
2L	23,513,712	24,266,334	24,085,515	23,885,995
2R	25,286,936	25,878,355	25,269,289	25,586,733
3L	28,110,227	28,948,966	28,897,970	28,636,850
3R	32,079,331	34,070,894	34,418,861	34,392,019
4	1,348,131	1,303,249	1,416,745	1,393,402
X	23,542,271	27,447,821	27,727,683	28,052,005
Y	3,667,352	15,707,245	16,486,182	17,032,472

Supplemental Table 4. The length of major chromosome of Scaffolded HiFi assemblies compared to iso-1 Release 6

Assembly	Complete - Single Copy	Complete - Duplicated	Fragmented	Missing	QV (arms)	Kmer-completeness
iso-1 Rel6	3265 (99.39%)	10 (0.30%)	8 (0.24%)	2 (0.06%)	-	-
iso-1 HiFi	3266 (99.42%)	9 (0.27%)	8 (0.24%)	2 (0.06%)	49.9	99.2574 %
A4 HiFi	3261 (99.27%)	14 (0.43%)	8 (0.24%)	2 (0.06%)	56.1	99.3543 %
A3 HiFi	3266 (99.42%)	9 (0.27%)	8 (0.24%)	2 (0.06%)	55.3	99.33 %

Supplemental Table 5. The BUSCO score of the HiFi assemblies and iso-1 Release 6 (from *compleasm*) . Phred QV scores and kmer-completeness for the hifi assemblies. The QV score is average QV of major chromosomal arms (2L, 2R, 3L, 3R, X)
