# Supplementary material for: Genetic variation in recalcitrant repetitive regions of the Drosophila melanogaster genome
Source: Genome Res. 2025 Sep;35(9):2023–40. doi: 10.1101/gr.280728.125 (PMC12400953; doi:10.1101/gr.280728.125)
Supplement: Supplement 3 [file Supplemental_Note_1.pdf]

Supplemental Note 1

Genetic variation in recalcitrant repetitive regions of the *Drosophila melanogaster* genome

Harsh G. Shukla, Mahul Chakraborty, J.J. Emerson

Section 1 : Characterization of R1 Elements in the rDNA Flanking Region

The main text highlights a high density of LINE R1 elements in the newly assembled sequence distal to the X-linked rDNA array (see Main Text, Fig 2B-C). Given the known preference of R1 elements for the 28S rDNA gene, we performed a detailed structural analysis to determine if the R1 elements in this flanking region retain the features of the canonical sequences found within the rDNA array itself.

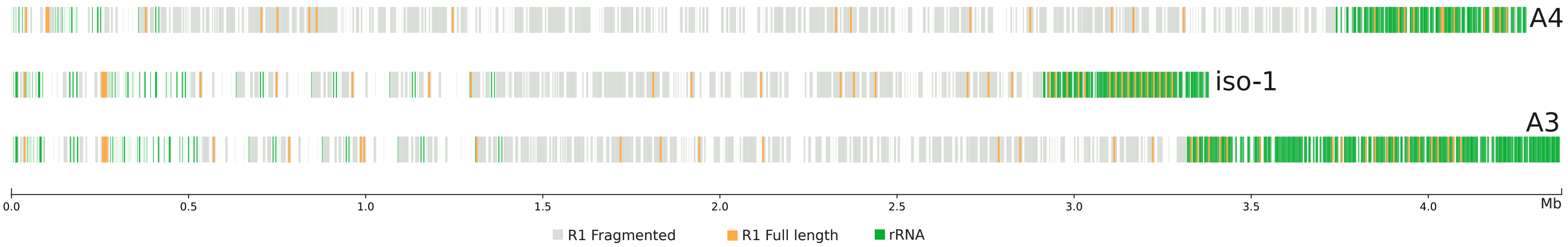

Figure 1.1: A re-colored version of the main Fig 2C. The fragmented R1 are marked by grey color whereas the “full” length R1 are highlighted by orange color. The rDNA and rDNA associated satellites are marked with green color.

To highlight the distribution of R1 elements in more detail, the region shown in the main manuscript's Fig. 2C is re-colored in Figure 1.1. In this figure, only R1 and rDNA & rDNA associated satellites are highlighted. For this analysis, a full length canonical R1 sequence of 5,356 bp was used to scan each strain with RepeatMasker. If the entire query (5356 bp minus a 2 bp offset/buffer) was covered in a particular alignment, it was considered a full length R1 sequence; otherwise, it was designated as fragmented. The light gray color marks the identified partial fragments of R1 sequences, whereas the orange marks the full length (though, as will be shown, some full length R1s possess internal indels). The rDNA & associated sequences are also marked green to highlight the partially assembled rDNA array.

The results show that all the R1 sequences in the rDNA array are full length, whereas most of the R1s in the adjacent distal region are partial fragments. Though several full-length sequences exist in this distally adjacent region, these elements almost always possess deletions ranging from 21-241 bp, with only one exception in iso-1 and one in A3. The insertions in the rDNA array proper are truly full length, with alignment covering the entire sequence with a maximum 10 bp deletion (most of them 1-3 bp deletions). This suggests that the “full” length R1 sequences in the distal region are much older. Most of these R1 insertions occur in tandem clusters, possibly tracing their origin to the initial tandem expansion (see Discussion and Discussion of (McGurk and Barbash 2018)). The identified “full” length R1 sequences might be the “surviving” ones that did not have their structure significantly altered through multiple rounds of deletions, other TE insertions and recombination.

Section 2 : Comparison of Pacbio HiFi Assemblers for the Histone Locus

The choice of assembler is a critical step for resolving highly repetitive regions. The initial hifiasm-based approach, for instance, failed to assemble the entire histone cluster in the A3 strain, and some ambiguity remained in other cases where multiple assembly paths were possible. To address this, the performance of other long-read assemblers was evaluated. The results confirmed that our targeted hifiasm assemblies provided the most contiguous and complete models of the histone locus for the strains in this study.

Our comparative analysis focused primarily on hifiasm (Cheng et al. 2021) versus verkko (Rautiainen et al. 2023), as verkko is widely adopted and considered a reliable, user-friendly alternative. A third assembler, LJA (Bankevich et al. 2022), was also evaluated.

Background

Ambiguity in assembly may be due to rare variation. Because the protocols employed here require a large amount of starting material, we sequence flies in bulk (~200 in this study). Formally speaking, there are up to ~400 nearly identical haplotypes segregating in the sequenced pool. The strains employed are thoroughly isogenized through genetic means (iso-1) or through intensive sibling mating of a strain that has already been propagated for decades at a very small population (A3 and A4). However, in highly mutable regions, variation may nevertheless accumulate quickly. Consequently, different versions of a “strain” in different labs may exhibit different variants and variation can even segregate within vials of the same strain kept in a single lab (Solares et al. 2018). This effect will be more pronounced in regions which have high mutation rates such as the ones we are currently studying. For example, our original hifiasm iso-1 alternate assembly had ~4% single-copy BUSCO genes within 10Mb of small haplotigs (N50 37kb). This represents the segregating variation in our fly dataset.

Without sufficient information, verkko is by default conservative. Consequently, it may not perform optimally on datasets that do not play to its strengths. verkko is based on the multiplex de Bruijn graph approach. It is designed to work with both HiFi (Long Accurate - LA) and Ultra-long nanopore reads (UL). It initially builds a graph based on LA reads and relies on UL reads to resolve & locally phase tangles in the graph. It can also use other orthogonal datasets such as HiC to get phasing across entire chromosomes. If there is no other information (like UL reads) given, it will not try to resolve regions that are ambiguous in the LA/HiFi graph and will report sequences of all unitigs. In that sense it is very conservative since it was designed to take advantage of additional long range information in a later stage of the pipeline. In our experience hifiasm can be aggressive in resolving tangles (the default histone assembly in the main manuscript) and assemblies across such regions would benefit from further QC. This is a tradeoff between contiguity and misassembly and the behavior of a particular assembler depends on such design tradeoffs.

Approach

We employed the latest version of verkko (v2.2) on our datasets. We observed similar contiguity metrics between hifiasm and verrko (Fig 2.1A, note the Y-axis scale), though hifiasm is slightly more complete and contiguous. We believe this is because, though our samples are virtually haploid, any small amount of segregating variation in the pool could cause multiple paths through the graph. Without additional information, verkko will not force resolution through such regions. Fig 3.1B presents some evidence for this reasoning. We take BUSCO duplicates as a proxy for alternate “haplotigs” in the final assembly. We run verkko using all the data versus longest 60X subsampled data for all three strains. As seen in figure 2.1B, for all three strains the duplication rates decrease for the “all vs long 60X” comparison. The “hifiasm all” stats are reported here to provide the “baseline” level of duplicates in the assembly.

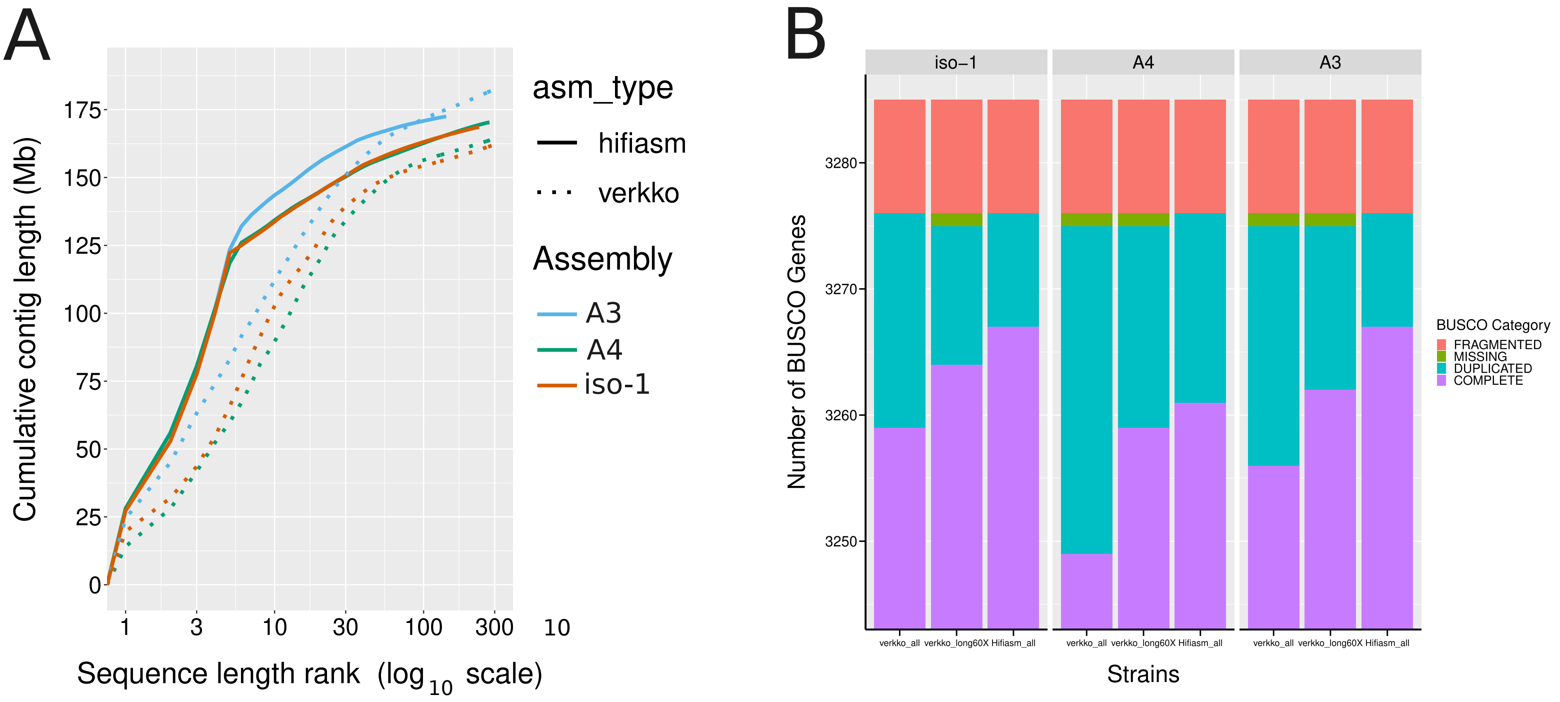

Figure 2.1 (A) Cumulative length plots comparing hifiasm and verkko assemblies **(B)** Zoomed in portions of the top of the BUSCO chart comparing verkko assemblies from “all vs longest 60X data”. The “hifiasm all” stats are provided here as baseline. Note the hifiasm stats are slightly different from ones presented in Supplemental table 5 since we are using an updated and improved version of the software (compleasm)

We initially intended to also use LJA in the above comparison, but due to its failure to run to completion for A3 and A4, its lower adoption compared to hifiasm and verkko, and the fact that it is no longer actively maintained (its most recent updates was Jan 2022), the following comparisons involving LJA are supplemental only. We attempted to assemble the longest 40-fold histone locus identified reads (i.e., 40-fold targeted dataset) for verkko and LJA. This subsampling is intended to minimize the impact of low frequency segregating variants. Despite our best efforts, targeted approaches did not run to completion for verkko. As a result, we used

the histone locus in assemblies from the longest 60-fold (i.e., 60-fold genome-wide dataset) for subsequent comparisons. For context, the longest 60-fold roughly translates to the longest 40-45-fold specifically at the histone locus since the histone locus has slightly lower coverage (see Section 3).

Contig comparisons from iso-1

Figure 2.2 Panels A, B, and C show results for the iso-1 histone assembly. Fig 2.2A shows the IGV tracks of alignments between our original targeted assembly as reference and LJA_all (first track) & verkko_60-fold (second track). The targeted 40-fold assembly for LJA did not yield a contiguous assembly, but the one with all the data assembled the locus into one single contig. We used that assembly as the LJA representative for iso-1 going forward. It assembles the locus with 113 copies and its structure matches exactly to our original assembly (Fig 2.2A first track). We identified a few indels in the LJA assembly relative to our hifiasm assembly. These indels are of very simple sequences with motif TAAA being part of 7 out of 8 deletions. This likely indicates that LJA suffers from consensus errors in simple sequences such as homopolymers, di/tri-nucleotide repeats etc. in the histone locus. The verkko_long60× (Fig 2.2A second track) assembly matches to the base pair with our original reference assembly except for a 2,366 bp insertion. Upon further examination we find that this is a tandem duplication. By mapping the reads to the verkko_long60× assembly (Fig 2.2C) we find that the majority of reads do bear a ~2,366 bp deletion in this region indicating that our original and LJA assembly faithfully reconstructed the “major” haplotype. We do find a single read spanning that lacks the deletion in the region, possibly explaining the presence of the tandem duplication in the verkko assembly as rare segregating or somatic variation. Fig 2.2B represents the homopolymer compressed (HPC) graph from verkko_long60× assembly. There is only a single unitig that incorporates the histone locus. The verkko_all data assembly also shows the same result. This indicates that iso-1 haplotype of the histone locus is relatively “easy” to assemble thereby explaining the success of 3 assemblers to reconstruct it.

Contig comparisons from A4

Figure 2.2 Panels D and E show results for the A4 histone assembly. For A4, the discussion is less straightforward, but we think it is instructive. In addition to the relatively minor structural changes that you might expect in such regions with variation segregating at low frequency (e.g., discussion of iso-1 above) that don’t disrupt consensus between the three assemblers, we also see all three assemblers coming to slightly different models of the region in the most contiguous haplotigs. Moreover, 2 of the 3 assemblers present fragmented models of the region whereas only hifiasm presents a single contig. Importantly, the flanks do present a unified consensus. And the differences in the middle are both relatively small and consistent with alternate graphs and/or alternate paths in similar graphs common in such complex regions. Nevertheless, the high level structure of the locus for A4, even when examining the unitigs directly, is broadly consistent with our discussion in the manuscript.

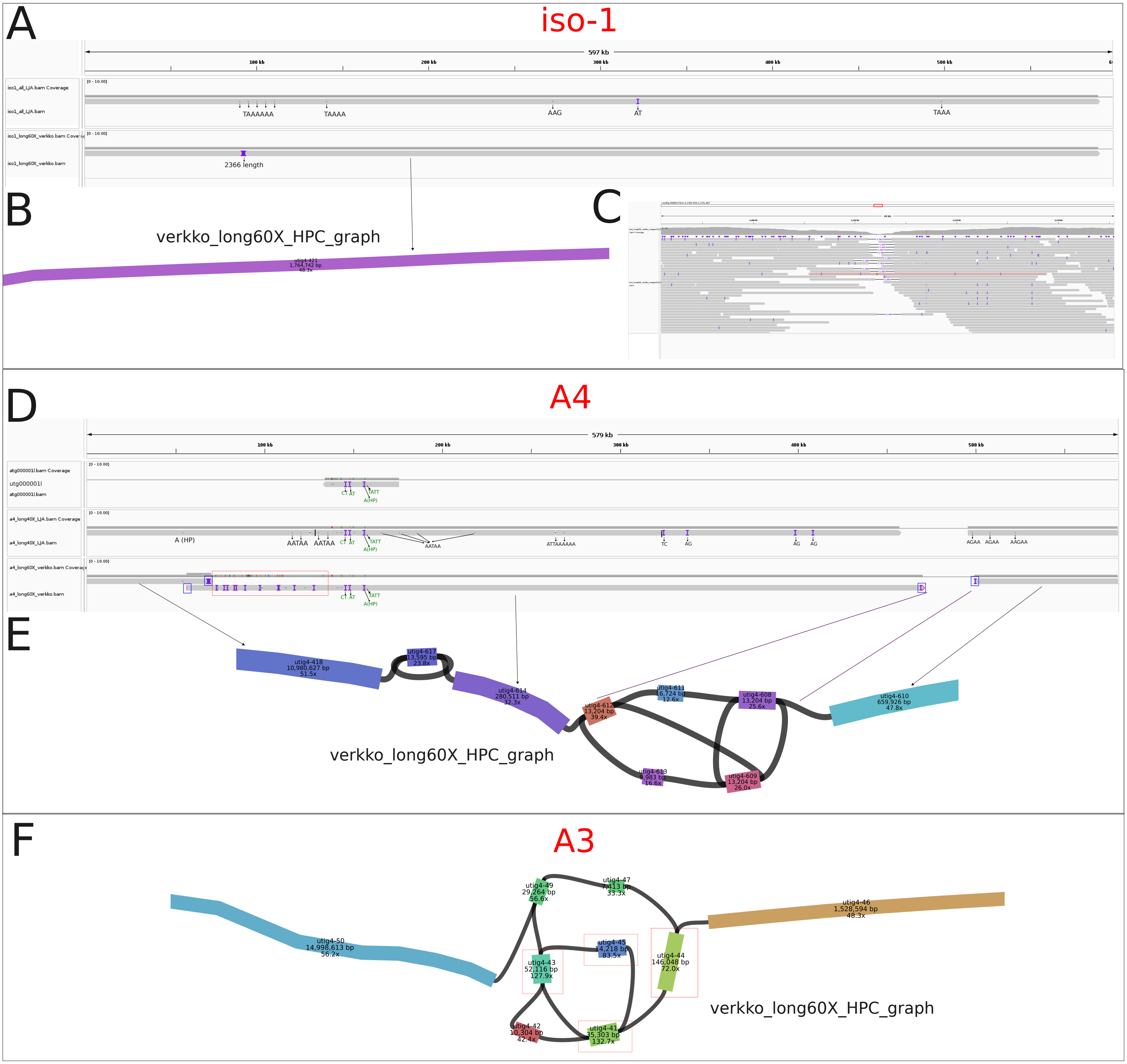

Figure 2.2 (A) The iso-1 (all) LJA and iso-1 (longest 60X) verkko histone assemblies were mapped to our original (described in the manuscript) histone assembly. The IGV plot shows the alignment; the indels were annotated. **(B)** A bandage plot displaying verkko's (longest 60X) homopolymer compressed (HPC) graph around the histone locus for iso-1. The first line in the label is the node name, the second is the length of the node and the third line is the depth **(C)** IGV alignment plot showing the mapping of reads

near the ~2366 bp duplication. The single read supporting the duplication is highlighted by red transparent box **(D)** The alternate A4 assemblies were mapped to our original histone assembly. The IGV plot shows the alignment; the indels were annotated. The first track shows the alignment of an alternate haplotig from our original hifiasm assembly. This haplotig/unitig was highlighted by a blue circle in the unitig graph in Supplemental Fig 14. This track is presented here for comparison since verkko and LJA assemble this alternative haplotype. The second track represents the mapping of 4 major contigs from *LJA* assembly. The black lines delineate the mapped contigs when it is not visibly apparent. Few smaller indels are annotated, likely representing consensus errors. The third track represents the mapping of the 3 major contigs from verkko assembly. The red box highlights a region that differs from our original and LJA assembly **(E)** A bandage plot displaying verkko's (longest 60X) homopolymer compressed (HPC) graph around the histone locus for A4. **(F)** A bandage plot displaying verkko's (longest 60X) homopolymer compressed (HPC) graph around the histone locus for A3. Nodes having depth significantly higher than (~40-50X) are highlighted in red. These nodes possibly reflect very recent and large duplications.

Contig comparisons from A3

For all three assemblers, the locus is fragmented, and thus we already rely less on assembly based conclusions. For A3, LJA (longest 40✕) assembled the locus into 16 contigs (~145 histone units). Ignoring <15kb contigs we get a total of 9 contigs (~138 units). Fig 1.3F shows the unitig graph for verkko (longest 60✕) for the histone cluster. It reports sequences of all the 9 unitigs and amongst them there are ~163 histone units present. As previously seen in the graph of our original hifiasm assembly (Supplemental Fig16B), there is a complex region in the middle in the verkko graph (Fig 2.2F) which is difficult to resolve. Upon further examination we also see some of the unitigs having 2 to 3 times the estimated coverage of ~40-45✕. These are highlighted in a red dotted box. They likely correspond to very recent large duplications and recapitulate the discussion in our main manuscript. Very large duplications that happened recently, especially ones in repetitive regions, create repetitive long homogeneous repeat structures that cannot be solved by HiFi data alone.

Broadly, these analyses support our original conclusions and show that the study's findings would not have been substantially different had an alternate assembler been used. We do acknowledge that our assemblies, like all assemblies, are merely models. As with any model, they are likely imperfect and subject to revision.

Section 3 : Orthogonal Validation of Histone Locus Copy Number Estimates

To validate the histone copy number estimates derived from genome assembly and Illumina read depth, a dPCR assay was conducted on recent nucleic acid extractions from our strains and six extreme outliers from the GDL datasets. The dPCR results confirmed a large magnitude of copy number variation across these strains (Supplemental File 6). While the dPCR estimates for strains iso-1 (113 copies) and A3 (204 copies) aligned well with other methods, the estimate for A4 (161 copies) was markedly higher than the assembly and Illumina results. This difference for A4 could potentially be due to sampling a different, more recent histone copy number variant with dPCR than the allele sequenced with HiFi and illumina reads. To rule out assembly error contributing to histone copy number in the assemblies, we further examined the Histone copy number in A3, A4, and iso-1 using an independent informatics approach using kmers, which also supports the Histone copy number estimates we reported in our main manuscript.

To briefly summarize our kmer-based work: The size of any given locus can be calculated using the total amount of base pairs (N) belonging to the locus and the genome wide coverage (C-fold). The ratio (N/C) will yield the locus size in a haploid (1-fold) genome. If we identify all reads belonging to the Histone locus and sum up lengths, we get N. From that, we can divide genome wide coverage C to get an estimate of the histone locus size. We can derive putative copy numbers by dividing the histone locus size by 5,045 (size of a canonical histone unit). For the strains (iso-1, A4) for which we have an assembly, we observe that the histone locus has lower long-read coverage than the genome wide coverage (see Fig. 3.1A, B). Thus, we couldn't use the genome wide coverage as a proxy for coverage in the histone locus. So we employed a k-mer based approach to estimate coverage in the histone locus. This approach (UCD Bioinformatics Core Workshop) is based on the same idea we previously used to derive the genome wide read coverage (see Supplemental Fig 2,3,4) . This approach is independent of mapping based coverage estimates and can be used to derive approximate coverage for a dataset when the genome/locus size is not assembled/known.

The reads belonging to the histone locus were identified de novo following commands in srf (Zhang et al. 2023) *(https://github.com/lh3/srf)*. While reads were previously identified by mapping to a default assembly, that approach potentially suffers from mapping biases, prompting the use of this *de novo* method. The length of all identified reads was summed to get an estimate of total base pairs (N) belonging to the histone locus. These reads were then used to derive the kmer frequency spectrum using Genomescope2 (Ranallo-Benavidez et al. 2020) (http://genomescope.org/genomescope2.0/). The homozygous kmer-peak derived from GenomeScope results was used to estimate the coverage (C) in the histone locus. The histone locus size was then estimated by taking the N/C ratio, and the putative copy number was calculated by dividing the histone locus size by 5,045.

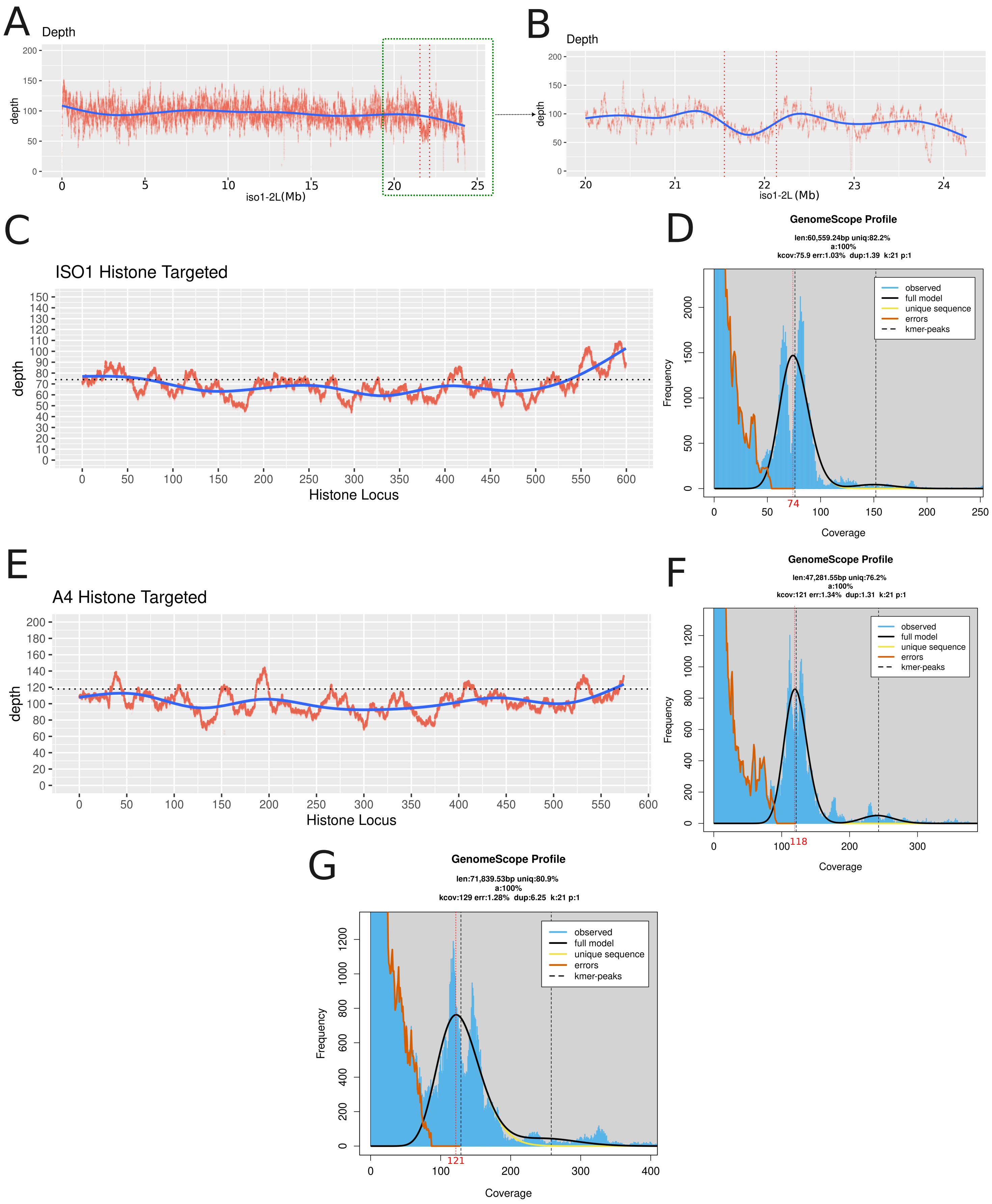

Figure 3.1: (A,B) The plots showing drop in coverage in the histone locus. The Y-axis is depth whereas the X-axis is the chromosome location for the 2L arm. The vertical red dashed lines indicate the start and

end of the histone locus. The blue line is the fit curve. Fig A is the entire 2L and Fig B is zoomed in for the last ~4 Mb. **(C,D)** Fig C is the depth plot for targeted histone locus for iso-1. It was also presented here (Supplemental Fig 10). The blue line is a fit curve and the black dotted horizontal line is the kmer-peak represented by the red dotted line in Fig D. Fig D is the genome scope kmer-spectra derived from identified histone locus reads in iso-1. The red-dotted vertical line (74) is the manually adjusted kmer-peak that represents/estimates read coverage in the histone locus. **(E,F)** Fig. E is the depth plot for targeted histone locus for A4. It was also presented here (Supplemental Fig. 13). The blue line is a fit curve and the black dotted horizontal line is the kmer-peak represented by the red dotted line in Fig. F. Fig. F is the genome scope kmer-spectra derived from identified histone locus reads in A4. The red-dotted vertical line (118) is the manually adjusted kmer-peak that represents/estimates read coverage in the histone locus. **(G)** The kmer spectra derived from identified histone locus reads in A3. The read-dotted vertical line (121) is the manually adjusted kmer-peak that represents/estimates read coverage in the A3 histone locus. Depth and coverage are used interchangeably here in all the figures.

Genomescope analysis of iso-1 histone reads (Fig 3.1D) initially estimated a raw k-mer modal peak (C) at 75.9, which was manually adjusted to 74 (red dotted line) for a better fit (this relatively minor tweak is not very significant, but does capture the central tendency of the distribution better than the specific parameterization of *Genomescope2*, which might be dragged up by tail). Fig. 3.1C shows the depth plot for targeted assembly for the histone locus in iso-1 (also presented in Supplemental Fig. 10). The black horizontal line (at 74) highlights the coverage estimates from the k-mer method. This k-mer coverage is slightly higher than mapping-based estimates for iso-1. For A4, the default k-mer peak of 121 was similarly adjusted to 118 (Fig. 3.1F), resulting in a k-mer coverage (118) significantly higher than mapping-based estimates (Fig. 3.1E). This difference was much more pronounced than in iso-1. For A3, the adjusted k-mer peak was 121 (default 129) (Fig. 3.1G).

The numbers for the current analysis are tabulated in Table below. We estimate the A3 histone locus size to be approximately 1.092 Mb, corresponding to roughly 216 copies. The k-mer based estimate for the iso-1 locus size corroborated findings from genome assembly. Our A4 estimates from assembly, Illumina read mapping, and k-mer counting were generally concordant, and critically, all were significantly lower than the A4 copy number estimated via dPCR.

Strain	Reads belonging to histone locus	Total base pairs in the reads (N)	Histone locus coverage (C) (kmer peak)	Estimated histone locus size (N/C)	Assembled size	Estimated histone CN (Size/5045)	Assem -bled histone CN	Illumina based CN	dPCR copy number
iso-1	2768	43637741	74	589.7 Kbp	~587 kbp	~117	113	102	113
A4	4385	62482683	118	529.5 Kbp	~571 kbp	~105	111	102	161
A3	7669	132120921	121	1.092 Mbp	-	~216	-	197	204

Section 4 : Considerations for a Complete Telomere-to-Telomere Assembly of *D. melanogaster*

The main manuscript's discussion, 'Persistent limitations in mapping the dark matter of the repetitive genome,' concludes that despite significant progress, resolving a complete, telomere-to-telomere (T2T) assembly of *D. melanogaster* remains a formidable challenge. This supplementary note expands on that conclusion by providing a more detailed perspective on the specific technical hurdles that must be overcome. Here, we explore the nature of the remaining gaps, which are almost exclusively large blocks of heterochromatic satellite DNA, and discuss how a combination of factors—from tissue-specific DNA properties to technology-specific sequencing biases—contributes to these unresolved regions. Finally, we outline why a multi-pronged strategy will likely be required to produce a truly complete and gapless assembly.

The first step in addressing these assembly challenges is to characterize the missing sequence. While the underlying causes are likely too complex to attribute to one factor, the amount and nature of the un-assembled regions can be estimated. We have assigned roughly 142 Mbp of the Drosophila melanogaster genome assembly to Muller elements (i.e., the autosomal arms + X Chromosome, excluding the Y Chromosome), which is approximately 80% of its genome size estimate of ~175 Mbp. This means we're missing about 33 Mbp of sequence, give or take. Among the ~142 Mbp we've assigned, we've recovered essentially 100% of the 117 Mbp of euchromatin (Hoskins et al. 2002) and an additional 25 Mbp of heterochromatin. The ~33 Mbp that we have yet to recover and assign is almost exclusively heterochromatin. Most of this is either large blocks of satellite sequence or islands buried in that satellite sequence. Why we as a community are not recovering and placing this sequence has not been rigorously established, though there are some very good hypotheses. A very recent preprint (Carvalho et al. 2025) explores this idea and, combined with our own hypotheses, several potential explanations emerge (including library biases, homogeneity of repeats, under replication of heterochromatin, physical properties of satellite regions, DNA extraction challenges such as repeat fragility, sequencing biases, base calling issues, and assembly difficulties). However, we find it hard to know which combination of these factors is truly explanatory. Moreover, they need not be mutually exclusive. More importantly, we've yet to identify a definitive approach to resolve these remaining gaps. Continued progress will be dependent on diagnosing the nature of the gaps that remain. Doing this properly will require a systematic exploration of the potential biases from the various sources mentioned earlier. (Carvalho et al. 2025) discuss these issues as well. That study, despite not proposing a definitive fix, serves to highlight important impediments to progress.

To put these potential challenges into a concrete context, we can examine the structure of currently assembled heterochromatin. Approximately 33.5% (59/176 Mb) of D. melanogaster Muller elements are heterochromatic, with the Y Chromosome (~41 Mb) being entirely heterochromatic (Fig. 4.1A). Heterochromatin adjacent to euchromatin contains transposable elements and unique sequences, which are well-assembled in the Rel6 reference. Towards the centromeres, highly repetitive satellite DNA blocks are encountered, hindering assembly and

consequently are absent in Rel6. These satellites include both simple and complex types. Preliminary analysis of our iso-1 HiFi assembly, compared to Rel6 scaffolds, suggests limited extension into centromeric regions, with the exception of 3R. Most improvements are in the form of gap-filling and contig extensions. Large satellite blocks are often absent or poorly represented in the iso-1 HiFi assembly, with some satellites found in isolated contigs within unscaffolded portions.

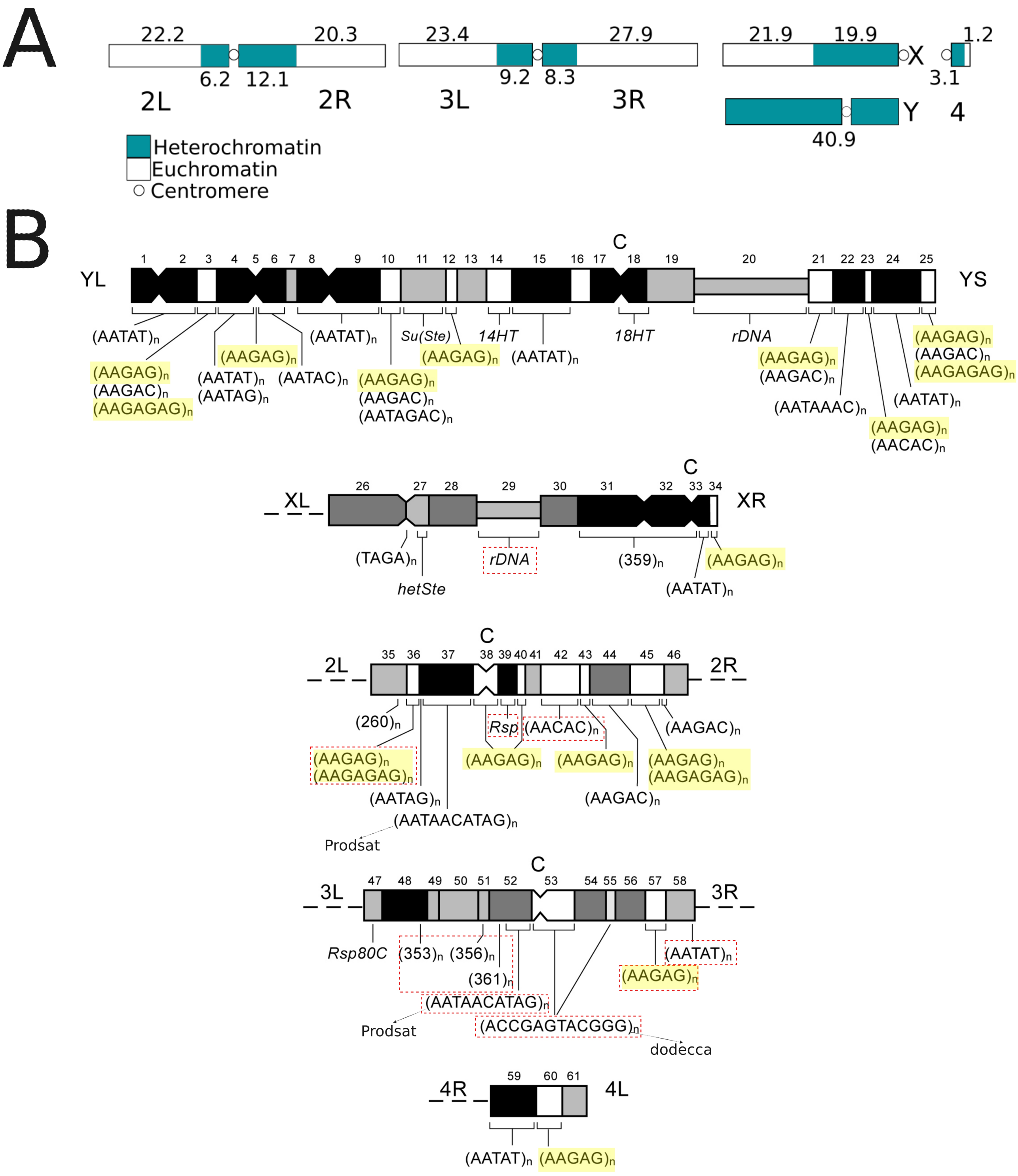

Figure 4.1 (A) The distribution of heterochromatin and euchromatin in chromosomes of *Drosophila melanogaster*. The numbers represent the estimated size. This figure is taken as is from (Hoskins et al. 2002) **(B)** Cytogenetic map of *Drosophila melanogaster* heterochromatin. This image is taken from (Hoskins et al. 2015) and modified. The red dotted box highlights some satellites which we can identify as possible causes of break / arm ends in our iso-1 HiFi assembly. Yellow boxes highlight the location of the most abundant satellites (AAGAG)n & (AAGAGAG)n in various arms.

Figure 4.1B illustrates heterochromatic bands and major satellite annotations in *D. melanogaster.* An inspection of our iso-1 HiFi assembly revealed that the majority of assembly gaps and all chromosome arm ends correspond to satellite regions. As the first step in our investigation, we assessed potential sequencing biases in the PacBio HiFi data by comparing its satellite DNA profiles against those from an Illumina PCR-free dataset. Both datasets were derived from iso-1 adult males, matched for developmental stage and sex. Table 4.1 below presents the proportion of known simple satellites in both datasets.

Satellite	Pacbio Hifi (%)	Illumina PCR-free (%)	Rank - HiFi (by abundance)	Rank - illumina (by abundance)
AAGAG	~0 %	0.622 %	-	1
AAGAC/AAAGAC	0.355 %	0.214%	1	3
AAGAGAG	~0 %	0.185 %	-	4
AACAC	0.219%	0.170 %	3	5
dodecca (11/12-mer)	0.246 %	0.314 %	2	2
AATAACATAG (prodsat) - 10mer	0.076 %	0.047 %	4	8
AATAT / AAAATAT	0.013 %	0.015 %	-	-

Table 4.1. Comparison of satellite abundance profiles from PacBio HiFi and Illumina PCR-free raw sequencing data derived from iso-1 adult males.

Table 4.2 presents the estimated abundance of major satellite sequences across the various chromosome arms of *D. melanogaster*. Two specific satellites, (AAGAG)n and its variant (AAGAGAG)n, comprise approximately 7.5% of the total genome (Lohe et al. 1993). In datasets generated with Illumina sequencing, these satellites rank among the four most plentiful (Table 4.1). In contrast, their representation in HiFi datasets is negligible. A known bias of the PacBio platform against low-complexity, AG-rich sequences has been documented previously (Nurk et al. 2020). Because of the near-total lack of reads for these satellites in the HiFi data, we determined that it is not feasible to assemble these major genomic components using HiFi technology alone. Conversely, our analysis showed that other major satellite types were found in broadly similar proportions in both the HiFi and Illumina datasets (Table 4.1). However, it is

important to note that many of these satellites appear under-represented relative to their expected genomic percentages in the DNA extracted from adult males, which was the source for both sequencing types.

Satellite	X	Y	II	III	IV
$(AATAT)_n$	600	5800	10	630	2700
$(AATAG)_n$	8.1	310	200	30	78
$(AATAC)_n$	0	3500	0	0	0
$(AAAAC)_n$	0	400	0	0	0
$(AAGAC)_n$	81	8500	1800	110	0
$(AAGAG)_n$	1200	7200	5500	1100	170
$(AATAAAC)_n$	0	1600	0	0	0
$(AATAGAC)_n$	0	1600	0	0	0
$(AAGAGAG)_n$	270	1800	1700	140	100
$(AATAACATAG)_n$/prod	0	0	1900	1600	0
359 bp	11000	0	0	0	0

Table 4.2. Estimated amounts (in kilobases, unless otherwise specified) of major satellite sequences in each chromosome in Drosophila melanogaster. This table was taken as is from (Jagannathan et al. 2017) (Table 4 in the manuscript). This table in turn was derived from estimates by (Lohe et al. 1993)

Achieving a telomere-to-telomere (T2T) genome assembly will likely depend on both the incorporation of new sequencing technologies and modifications to the DNA source material, such as using early embryos to address the under-replication of heterochromatin in adult tissues. HiFi sequences alone are insufficient due to their bias against (AAGAG)n repeats. Oxford Nanopore Technology (ONT) R10 data can be explored as a complement to HiFi for building the initial base graph. Furthermore, Ultra Long ONT reads may be crucial for resolving large satellite arrays and verifying assembly integrity in such regions. Complete assembly of the extremely repetitive Y Chromosome would likely present the most significant challenge of all.

References

UCD Bioinformatics Core Workshop. Available from: https://ucdavis-bioinformatics-training.github.io/2020-Genome_Assembly_Workshop/kmers/kmers

Bankevich A, Bzikadze AV, Kolmogorov M, Antipov D, Pevzner PA. 2022. Multiplex de Bruijn graphs enable genome assembly from long, high-fidelity reads. *Nat. Biotechnol.* 40:1075–1081.

Carvalho AB, Kim BY, Uno F. 2025. Strong sequencing bias in Nanopore and PacBio prevents assembly of Drosophila melanogaster Y-linked genes. *bioRxiv* [Internet]. Available from: http://dx.doi.org/10.1101/2025.02.23.639762

Cheng H, Concepcion GT, Feng X, Zhang H, Li H. 2021. Haplotype-resolved de novo assembly using phased assembly graphs with hifiasm. *Nat. Methods* 18:170–175.

Hoskins RA, Smith CD, Carlson JW, Carvalho AB, Halpern A, Kaminker JS, Kennedy C, Mungall CJ, Sullivan BA, Sutton GG, et al. 2002. Heterochromatic sequences in a Drosophila whole-genome shotgun assembly. *Genome Biol.* 3:RESEARCH0085.

Jagannathan M, Warsinger-Pepe N, Watase GJ, Yamashita YM. 2017. Comparative Analysis of Satellite DNA in the Drosophila melanogaster Species Complex. *G3* 7:693–704.

Lohe AR, Hilliker AJ, Roberts PA. 1993. Mapping simple repeated DNA sequences in heterochromatin of Drosophila melanogaster. *Genetics* 134:1149–1174.

McGurk MP, Barbash DA. 2018. Double insertion of transposable elements provides a substrate for the evolution of satellite DNA. *Genome Res.* 28:714–725.

Nurk S, Walenz BP, Rhie A, Vollger MR, Logsdon GA, Grothe R, Miga KH, Eichler EE, Phillippy AM, Koren S. 2020. HiCanu: accurate assembly of segmental duplications, satellites, and allelic variants from high-fidelity long reads. *Genome Res.* 30:1291–1305.

Ranallo-Benavidez TR, Jaron KS, Schatz MC. 2020. GenomeScope 2.0 and Smudgeplot for reference-free profiling of polyploid genomes. *Nat. Commun.* 11:1432.

Rautiainen M, Nurk S, Walenz BP, Logsdon GA, Porubsky D, Rhie A, Eichler EE, Phillippy AM, Koren S. 2023. Telomere-to-telomere assembly of diploid chromosomes with Verkko. *Nat. Biotechnol.* 41:1474–1482.

Solares EA, Chakraborty M, Miller DE, Kalsow S, Hall K, Perera AG, Emerson JJ, Hawley RS. 2018. Rapid Low-Cost Assembly of the Drosophila melanogaster Reference Genome Using Low-Coverage, Long-Read Sequencing. *G3* 8:3143–3154.

Zhang Y, Chu J, Cheng H, Li H. 2023. De novo reconstruction of satellite repeat units from sequence data. *Genome Res.* 33:1994–2001.
