# Supplementary material for: Genetic variation in recalcitrant repetitive regions of the Drosophila melanogaster genome
Source: Genome Res. 2025 Sep;35(9):2023–40. doi: 10.1101/gr.280728.125 (PMC12400953; doi:10.1101/gr.280728.125)

Supplemental File for

Genetic variation in recalcitrant repetitive regions of the *Drosophila melanogaster* genome

Harsh G. Shukla, Mahul Chakraborty, J.J. Emerson

Supplemental File 3: Pairwise phylogenetic trees for identifying putative homology in the histone locus. Blues boxes highlight the putative anchors identified in pairwise comparison. The red, purple and green boxes mark clades highlighting closely related repeat units. The colors correspond to ones used in main Fig. 4B.

T1. ISO1 HiFi – ISO1 Rel6. A phylogenetic tree build using all individual histone units from iso1 HiFi and iso1 Rel6 histone locus assemblies.

T2. ISO1 HiFi – A4 HiFi. A phylogenetic tree build using all individual histone units from iso1 HiFi and A4 HiFi histone locus assemblies.

T3. ISO1 HiFi – A3 HiFi. A phylogenetic tree build using all individual histone units from iso1 HiFi and A3 HiFi histone locus assemblies.

T4. A4 HiFi – A3 HiFi. A phylogenetic tree build using all individual histone units from A4 HiFi and A3 HiFi histone locus assemblies.

The naming convention of units is as follows:

STRAIN:	Strain name that a unit belongs to
LOCUS_NAME:	Name of the locus under investigation
UNIT NO:	The identity for a unit. The first unit in the array is labeled 1 and so on.
START:	The start coordinate of the unit in the array.
END:	The end coordinate of the unit in the array.
LENGTH:	The last column is the length of that unit.

T1. ISO1 HiFI - ISO1 Rel6

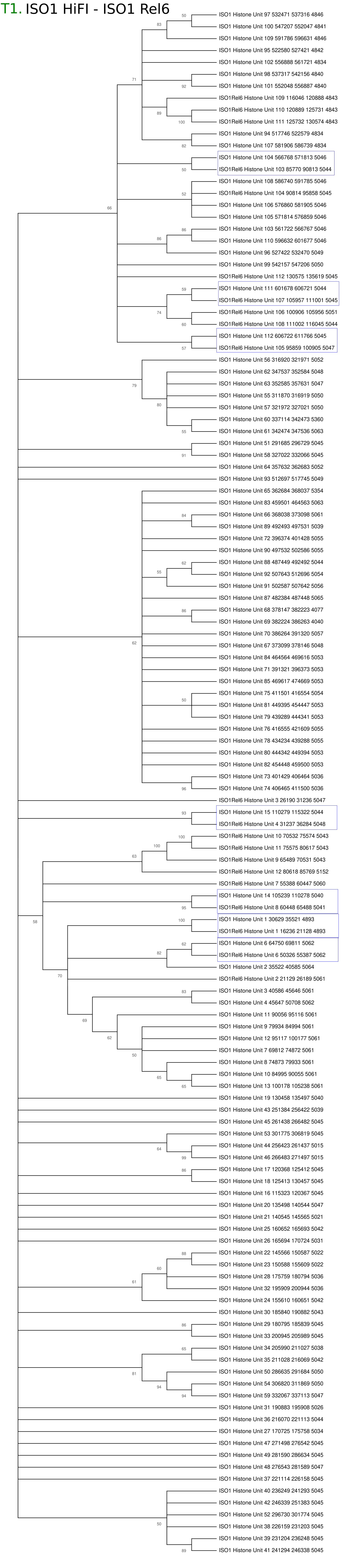

T2. ISO1 HiFi - A4 HiFi

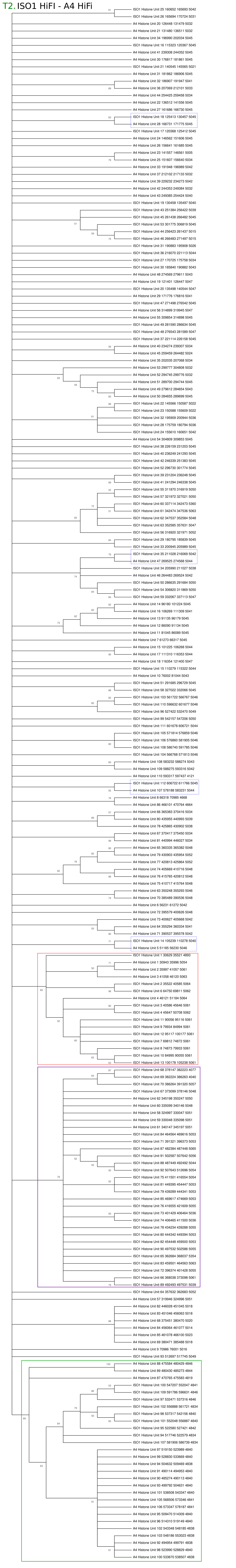

T3. ISO1 HiFi - A3 HiFi

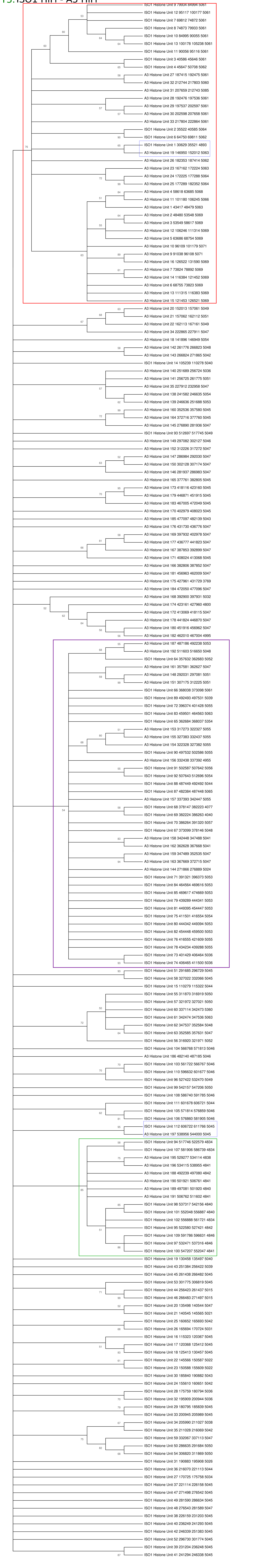

T4. A4 HiFi - A3 HiFi

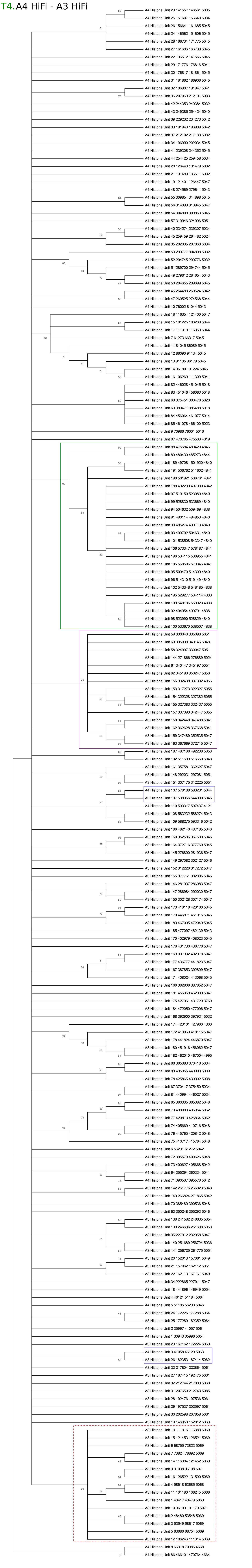

Supplement: Supplement 6 [file Supplemental_File_3.pdf]
