# Supplementary material for: Genetic variation in recalcitrant repetitive regions of the Drosophila melanogaster genome
Source: Genome Res. 2025 Sep;35(9):2023–40. doi: 10.1101/gr.280728.125 (PMC12400953; doi:10.1101/gr.280728.125)

Supplemental File for

Genetic variation in recalcitrant repetitive regions of the *Drosophila melanogaster* genome

Harsh G. Shukla, Mahul Chakraborty, J.J. Emerson

Supplemental File 4: Phylogenetic trees for analysis of PacBio HiFi assemblies in the *Stellate* locus. Blue boxes highlight the putative anchors identified in the comparisons.

T1. ISO1 A4 A3 Euchromatic Stellate locus. A phylogenetic tree build using all individual Stellate units from iso1 HiFi, A4 HiFi and A3 HiFi euchromatic Stellate locus assemblies.

T2. ISO1 A3 Het. L2 Stellate locus. A phylogenetic tree build using all individual stellate units from iso1 HiFi and A3 HiFi heterochromatic L2 Stellate locus assemblies.

T3. ISO1 A3 Het. L3 Stellate locus. A phylogenetic tree build using all individual stellate units from iso1 HiFi and A3 HiFi heterochromatic L3 Stellate locus assemblies.

The naming convention of units is as follows:

STRAIN:	Strain name that a unit belongs to
LOCUS_NAME:	Name of the locus under investigation
UNIT NO:	The identity for a unit. The first unit in the array is labeled 1 and so on.
START:	The start coordinate of the unit in the array.
END:	The end coordinate of the unit in the array.
LENGTH:	The last column is the length of that unit.

T1. ISO1 A4 A3 Euchroamtic Stellate Locus

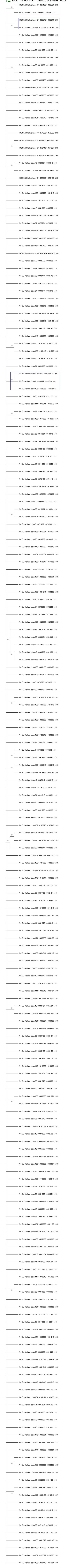

T2. ISO1 A3 Het. L2 Stellate Locus

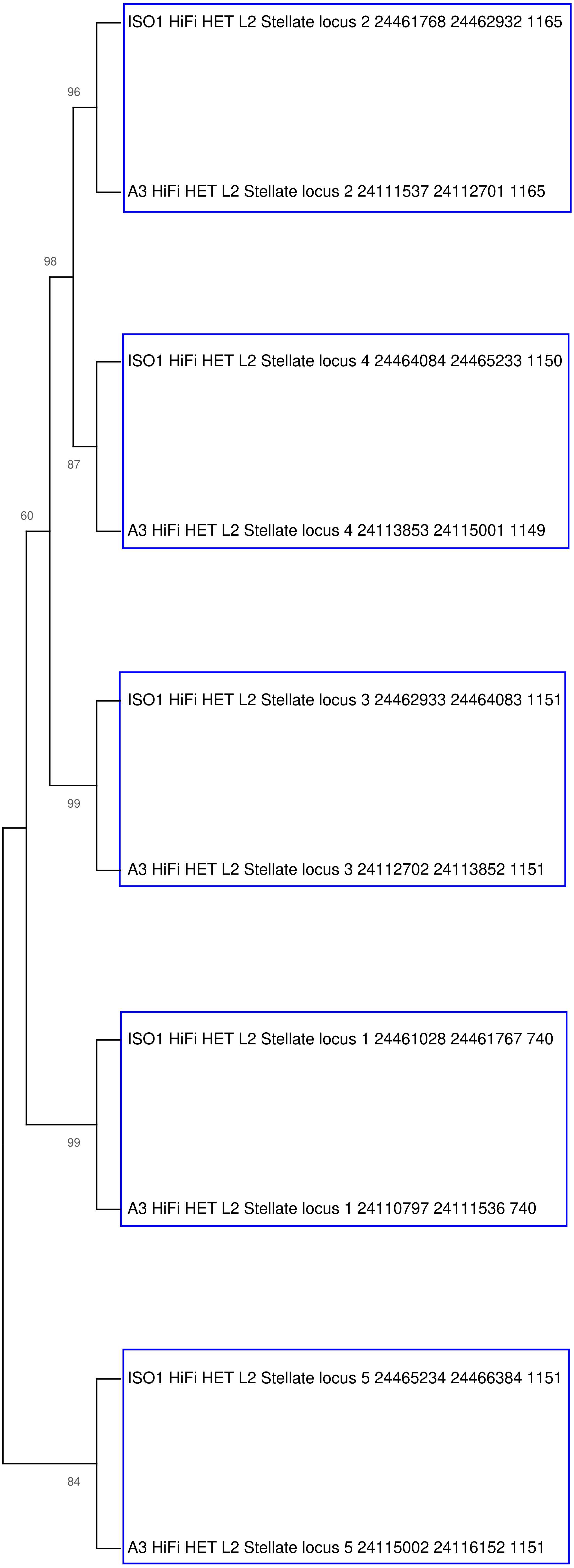

T3. ISO1 A3 Het. L3 Stellate Locus

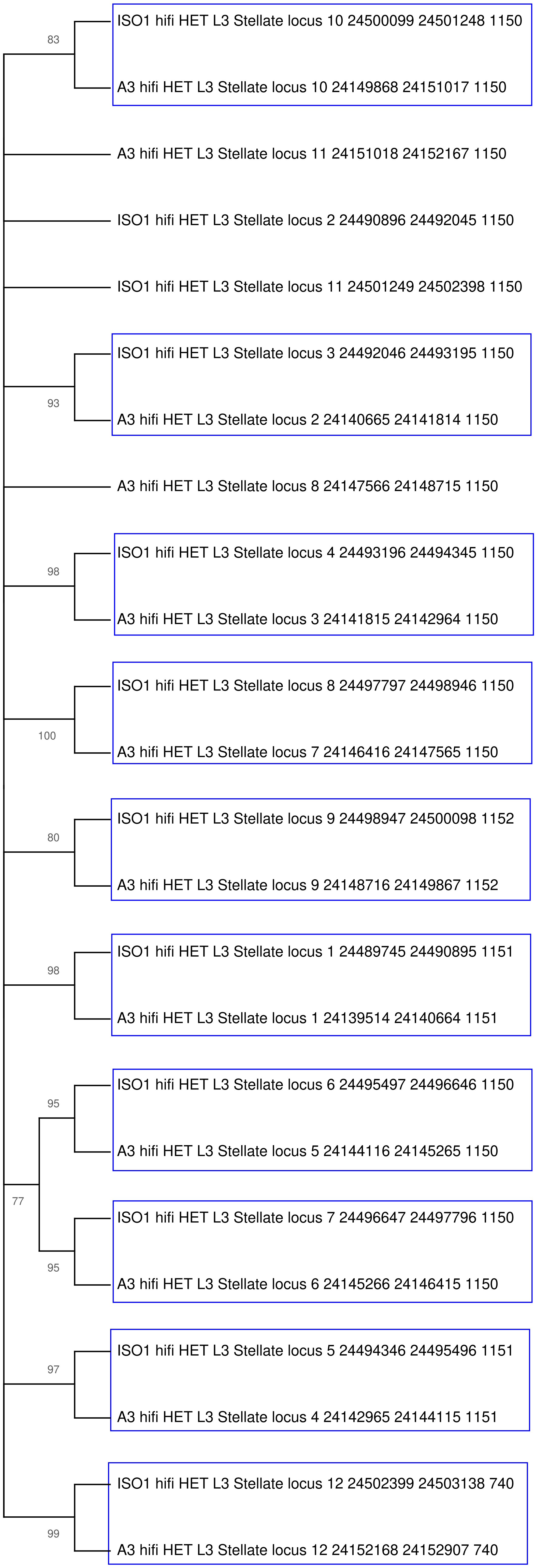

Supplement: Supplement 7 [file Supplemental_File_4.pdf]
