# Supplementary material for: Genetic variation in recalcitrant repetitive regions of the Drosophila melanogaster genome
Source: Genome Res. 2025 Sep;35(9):2023–40. doi: 10.1101/gr.280728.125 (PMC12400953; doi:10.1101/gr.280728.125)

Supplemental File for

Genetic variation in recalcitrant repetitive regions of the *Drosophila melanogaster* genome

Harsh G. Shukla, Mahul Chakraborty, J.J. Emerson

Supplemental File 5: Phylogenetic trees for analysis of all iso-1 assemblies in the Stellate locus (iso1 HiFi, iso1 Release 6, iso1 HetEnr). The blues boxes highlight the putative anchors identified in the comparisons.

T1. ISO1 HiFi and Rel6 Euchromatin Stellate locus. A phylogenetic tree build using all individual stellate units from iso1 HiFi and iso1 Rel6 euchromatic Stellate locus assemblies.

T2. ISO1 HiFi and HetEnr Stellate locus. A phylogenetic tree build using all individual stellate units from iso1 HiFi and iso1 HetEnr euchromatic Stellate locus assemblies.

T3. ISO1 HetEnr and Rel6 Stellate locus. A phylogenetic tree build using all individual stellate units from iso1 HetEnr and iso1 Rel6 euchromatic Stellate locus assemblies.

T4. ISO1 HiFi and HetEnr Het. L2 Stellate locus: A phylogenetic tree build using all individual stellate units from iso1 HiFi and iso1 HetEnr heterochromatic L2 Stellate locus assemblies.

T5. ISO1 HiFi and HetEnr Het. L3 Stellate locus: A phylogenetic tree build using all individual stellate units from iso1 HiFi and iso1 HetEnr heterochromatic L3 Stellate locus assemblies.

The naming convention of units is as follows:

STRAIN:	Strain/Assembly name that a unit belongs to
LOCUS_NAME:	Name of the locus under investigation
UNIT NO:	The identity for a unit. The first unit in the array is labeled 1 and so on.
START:	The start coordinate of the unit in the array.
END:	The end coordinate of the unit in the array.
LENGTH:	The last column is the length of that unit.

T1. ISO1 HiFi and Rel6 Euchromatin Stellate locus

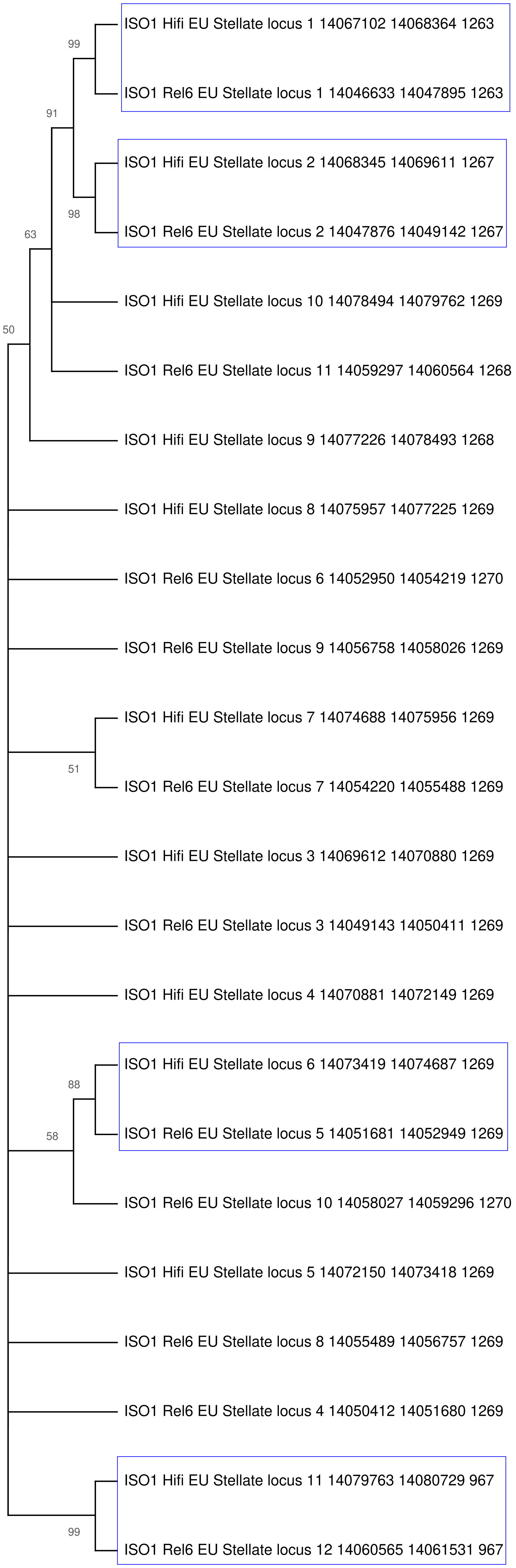

T2. ISO1 HiFi and HetEnr Euchromatin Stellate locus

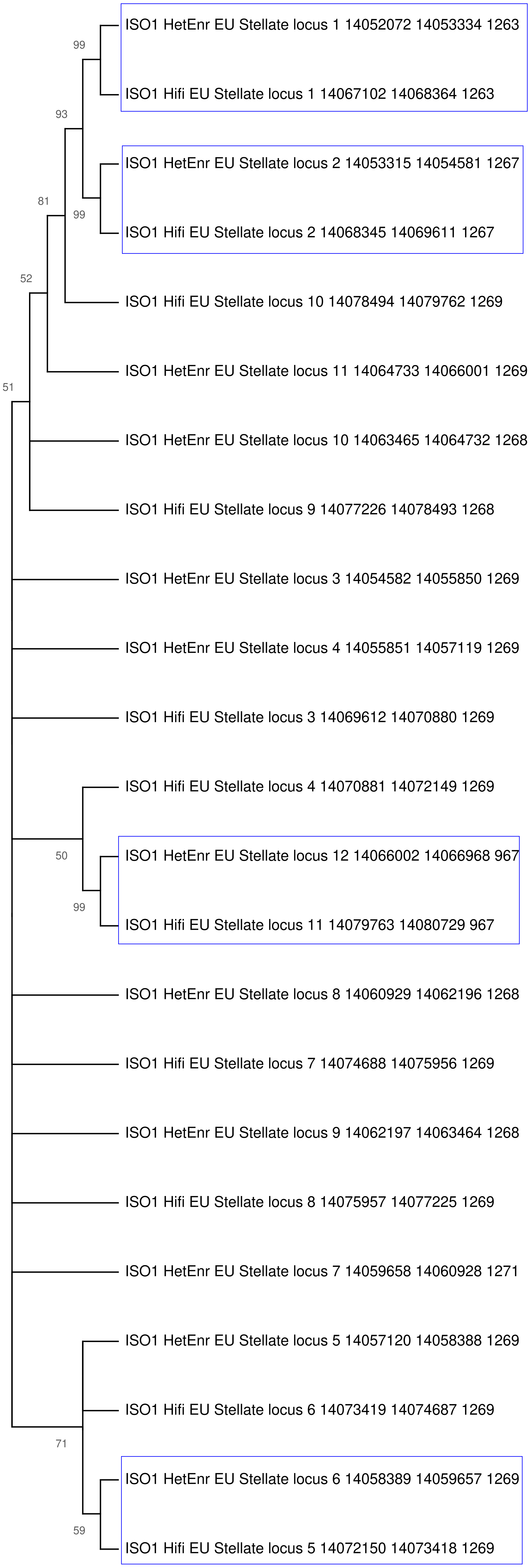

T3. ISO1 HetEnr and Rel6 Euchromatin Stellate locus

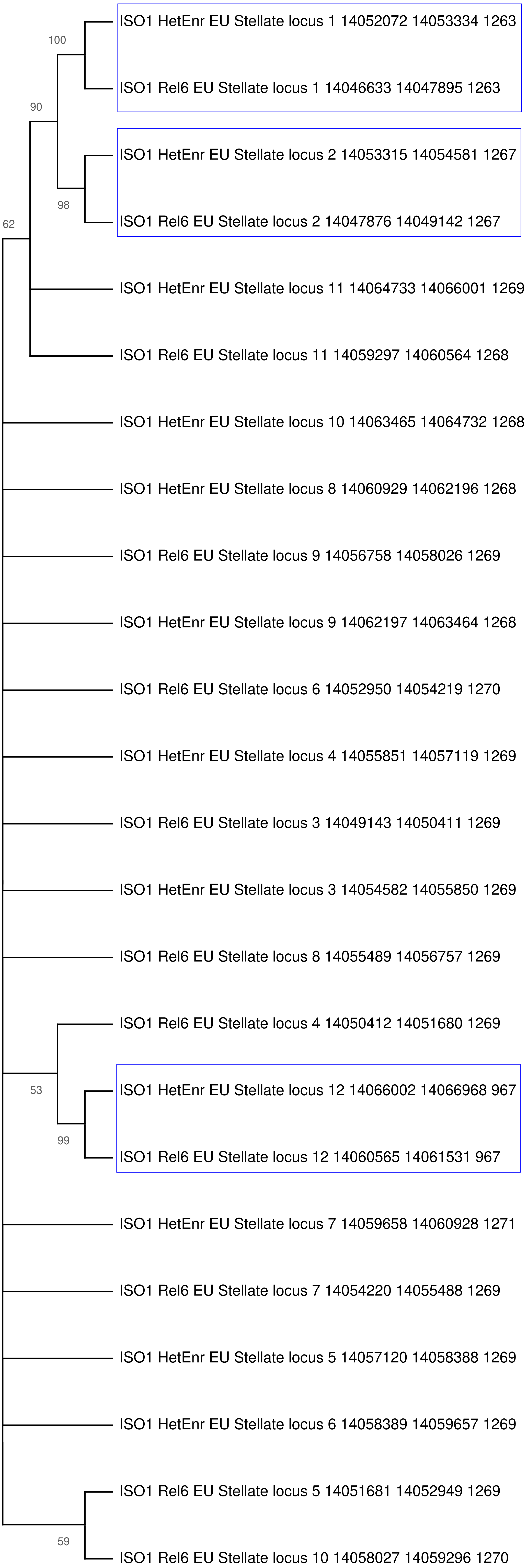

T4. ISO1 HiFi and HetEnr Het. L2 Stellate locus

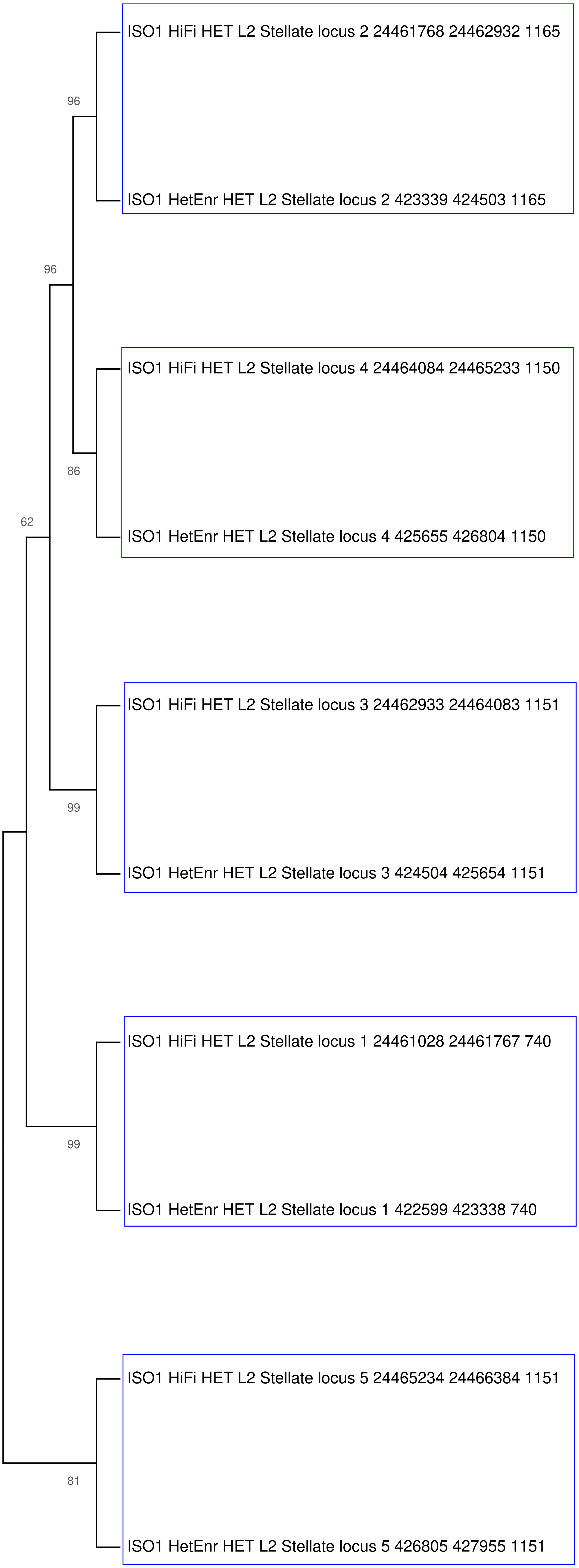

T5. ISO1 HiFi and HetEnr Het. L3 Stellate locus

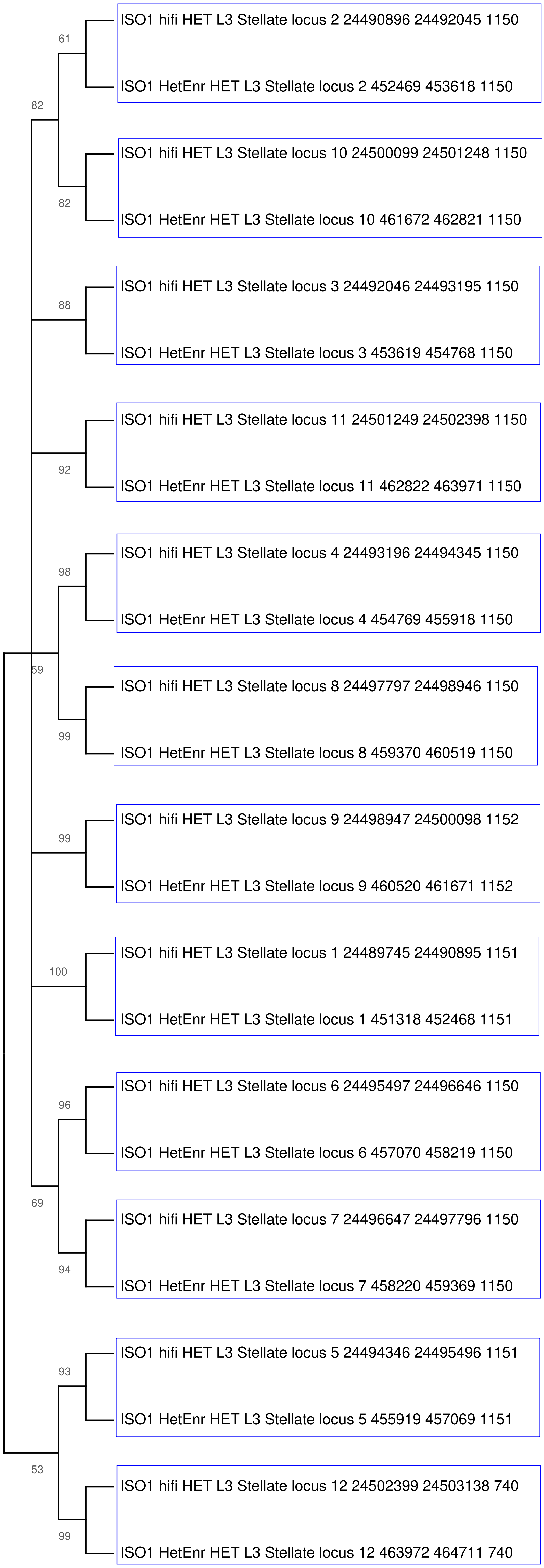

Supplement: Supplement 8 [file Supplemental_File_5.pdf]
