# Supplementary figures and images for: Genetic variation in recalcitrant repetitive regions of the Drosophila melanogaster genome
Source: Genome Res. 2025 Sep;35(9):2023–40. doi: 10.1101/gr.280728.125 (PMC12400953; doi:10.1101/gr.280728.125)

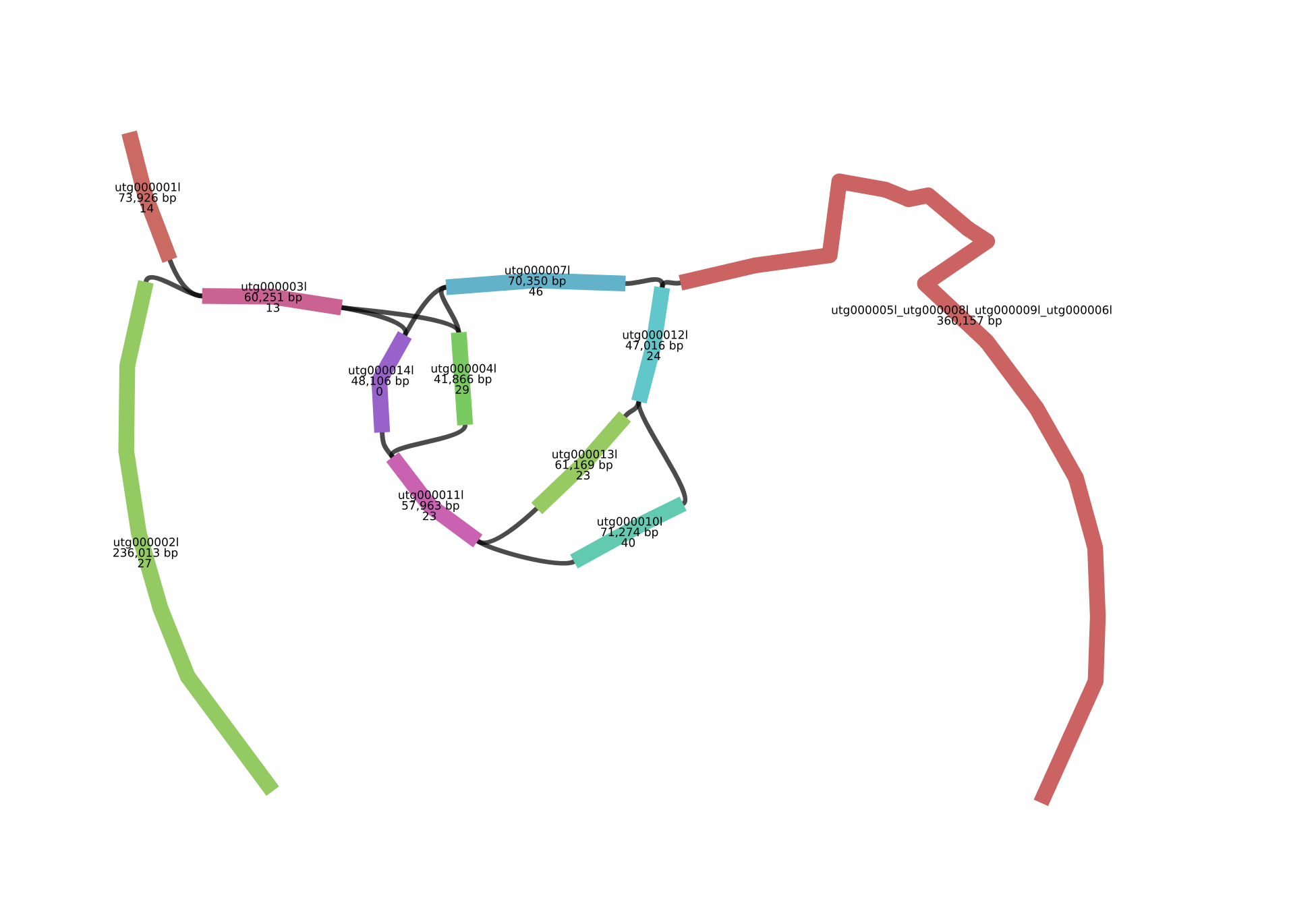

Supplement: Supplement 10 [file Supplemental_Code.zip › Dmel_HiFi_Asm_variation/Histone_Locus_Analysis/Targeted_genome_Assembly/A3_Well_resolved_ASM/After.png]

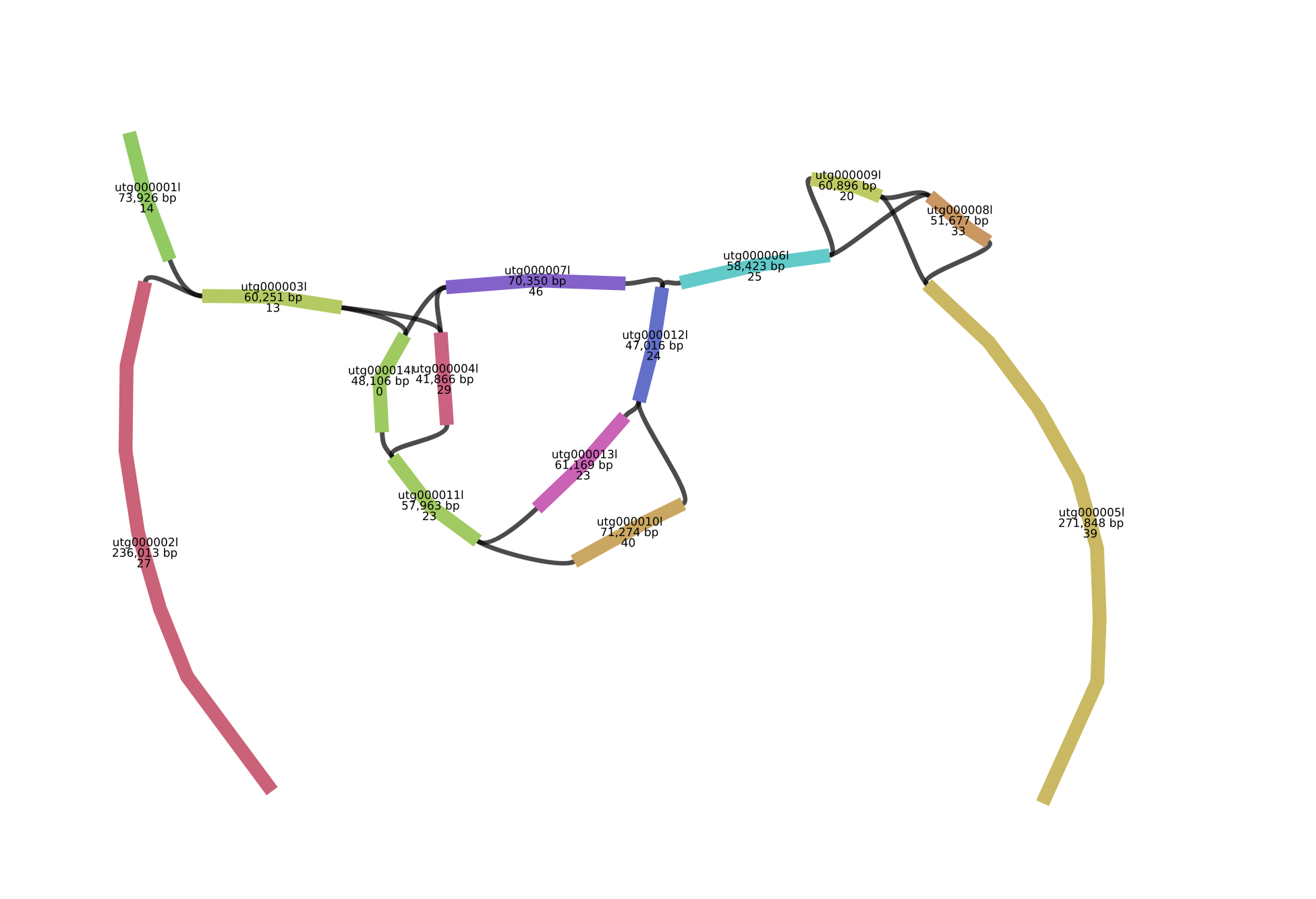

Supplement: Supplement 10 [file Supplemental_Code.zip › Dmel_HiFi_Asm_variation/Histone_Locus_Analysis/Targeted_genome_Assembly/A3_Well_resolved_ASM/Before.png]
